# What is the Difference between Conventional Drinking Water, Potable Reuse Water, and Nonpotable Reuse Water? A Microbiome Perspective

**DOI:** 10.1021/acs.est.4c04679

**Published:** 2024-09-11

**Authors:** Matthew F. Blair, Emily Garner, Pan Ji, Amy Pruden

**Affiliations:** † Via Department of Civil and Environmental Engineering, Virginia Tech, Blacksburg, Virginia 24061, United States; ‡ Wadsworth Department of Civil and Environmental Engineering, West Virginia University, Morgantown, West Virginia 26506, United States

**Keywords:** 16S rRNA gene amplicon sequencing, water reuse, drinking water treatment, wastewater treatment, molecular classification of water quality, next generation sequencing, core and discriminatory microbiota

## Abstract

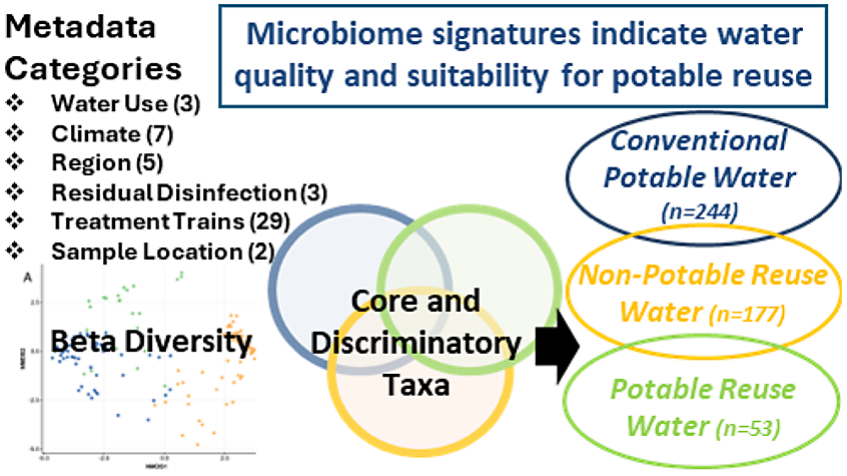

As water reuse applications expand, there is a need for more comprehensive means to assess water quality. Microbiome analysis could provide the ability to supplement fecal indicators and pathogen profiling toward defining a “healthy” drinking water microbiota while also providing insight into the impact of treatment and distribution. Here, we utilized 16S rRNA gene amplicon sequencing to identify signature features in the composition of microbiota across a wide spectrum of water types (potable conventional, potable reuse, and nonpotable reuse). A clear distinction was found in the composition of microbiota as a function of intended water use (e.g., potable vs nonpotable) across a very broad range of U.S. water systems at both the point of compliance (Betadisper *p* > 0.01; ANOSIM *p* < 0.01, *r*-stat = 0.71) and point of use (Betadisper *p* > 0.01; ANOSIM *p* < 0.01, *r*-stat = 0.41). Core and discriminatory analysis further served in identifying distinct differences between potable and nonpotable water microbiomes. Taxa were identified at both the phylum (Desulfobacterota, Patescibacteria, and Myxococcota) and genus (*Aeromonas* and *NS11.12_marine_group*) levels that effectively discriminated between potable and nonpotable waters, with the most discriminatory taxa being core/abundant in nonpotable waters (with few exceptions, such as *Ralstonia* being abundant in potable conventional waters). The approach and findings open the door to the possibility of microbial community signature profiling as a water quality monitoring approach for assessing efficacy of treatments and suitability of water for intended use/reuse application.

## Abstract 2

This study demonstrates the potential for profiling microbial community composition via 16S rRNA gene amplicon sequencing as a high-resolution tool for classifying water quality and its corresponding suitability for various water use and reuse applications.

## Introduction

Drinking water and wastewater systems provide rich environments that support taxonomically and functionally diverse microbial populations.^1−4^ In the wastewater sector, biological treatment is essential for the removal of biochemical oxygen demand (BOD), nitrogen,^5−7^ and phosphorus.^8−12^ Microbes in wastewater also contribute to degradation of contaminants of emerging concern.^13−17^ In the drinking water sector, microorganisms can also contribute to carbon and nutrient reduction,^18,19^ as well as microbial-induced corrosion^20,21^ and waterborne disease.^22,23^ Pathogenic microorganisms are of high concern in any water system. Regardless of where on the “one water” continuum^24^ a given treatment or water application may fall, microbial community composition is a key driver of the realization of water quality goals.^24^ As the water and wastewater industries move toward a fit for (re)use framework, systematic profiling of microbial communities could provide a comprehensive and high-resolution approach to water quality monitoring.

Advances in high throughput next generation DNA sequencing (NGS) have inspired an increasing number of studies aimed at characterizing the microbial communities, herein referred to as microbiomes, inhabiting various water environments. NGS has now widely been applied to profile microbes involved in drinking water and wastewater treatment processes,^4,25−28^ including assessing the impacts of operational conditions, such as: treatment technology employed,^29^ distribution system characteristics (e.g., pipe materials, water age, and water chemistry),^30^ disinfectant types and concentrations,^31^ and seasonal and source water variation.^32,33^ Others have examined the microbial communities that colonize nonpotable reuse water systems,^34,35^ various strategies for potable reuse (such as blending),^36^ membrane filtration,^37^ and carbon-based biofiltration,^29^ as well as more conventional drinking water treatment plants and distribution systems.^38^ However, such studies have largely been conducted in isolation, without direct comparison to identify shared versus distinct features represented across respective microbiomes. Such knowledge could help to define microbiome “signatures” as a high-resolution means of characterizing different water types that could supplement existing water quality testing, such as fecal indicator monitoring, heterotrophic plate counts, adenosine triphosphate (ATP) measurements, and flow cytometry.

A core microbiome consists of organisms that are found in common across a given set of microbiomes and are hypothesized to play a key role within the ecosystem of interest.^39^ The discriminatory microbiome, on the other hand, is made up of organisms that distinguish multiple ecosystems of interest.^39^ Previously, core microbiome analysis has been applied toward comprehensively characterizing and comparing human microbiomes, specifically through the human microbiome project^40,41^ and focused on important human niches, such as: oral,^42^ fecal,^43^ and gut^44,45^ microbiomes. To a lesser extent, core microbiome analysis has been applied to understand the microbial dynamics and composition occurring within the water treatment microbiome^38,46^ and wastewater microbiome.^47,48^ As NGS is beginning to become more widely applied in the water sector,^49^ there is a need to establish methodology for characterizing drinking water quality^50^ and further to support systematic comparisons across waters of different intended end uses. For example, microbiome analysis could provide high resolution analysis across the spectrum of nonpotable and potable reuse waters. Furthermore, microbiome analysis could take a key step toward supplementing fecal indicator and pathogen profiling and defining a “healthy” drinking water microbiota. Although recent studies have begun to reveal the vast diversity of potable water microbiomes,^51,52^ to our knowledge, no prior study has compared microbiomes across the spectrum of different water use/reuse categories.

Here we utilized 16S rRNA gene amplicon sequencing to identify signature features in the microbiomes associated with a spectrum of water types [potable conventional (bottled water, conventional drinking water), potable reuse (direct potable reuse (DPR), indirect potable reuse (IPR)), and nonpotable reuse]. Specifically, the objectives of this study were to: 1) identify the occurrence of differential microbial “signatures” related to intended water use, 2) utilize core and discriminatory analysis to identify taxa and/or operational taxonomic units (OTUs) that are specific to the various water types and assess the corresponding variance observed for each water type, and 3) assess shifts in key taxa as a function of various treatments, stages of treatment, disinfection techniques, stage of conveyance (point of compliance (POC) versus point of use (POU)), climate, and other variables. The approach and findings of this study open the door to the possibility of microbial community signature profiling as a water quality monitoring approach for assessing efficacy of treatments and suitability of water for intended use.

## Methods

### Site Description, Sample Collection, and Preservation

This study took advantage of a large archive of in-house samples and data sets collected across several prior studies representing a wide range of water types (e.g., conventional potable, potable reuse, and nonpotable reuse), treatment technologies, climates and regional locations,^36,38,53^ to provide a robust comparison across waters of various use classifications generated from a wide-variety of treatment approaches (Supporting Information Table 1). Additional samples were collected for this study from various brands of off-the-shelf bottled water and from a pilot-scale carbon-based IPR train over a period of 23 months. The IPR train took secondary effluent from a nearby treatment plant and treated it with coagulation-flocculation-sedimentation, ozone advanced oxidation (O_3_), biologically active carbon filtration (BAC), granular activated carbon filtration (GAC), and UV disinfection. Influent and effluent samples were collected to represent nonpotable and IPR water, respectively. Samples were classified as being representative of either the POC, i.e., directly after final treatment and prior to distribution, or the POU, i.e., after being conveyed through a distribution system, simulated distribution system and/or simulated building plumbing. In total, 474 samples and 30 blanks were analyzed with classification by intended water use and sample location provided in Supporting Information Table 2. Detailed metadata can be found in the Supporting Information Tables 3–6 and includes information related to climate, treatment technology employed, disinfection method and residual, and intended application.

All samples were collected with the same QA/QC standards, i.e., in sterile 1-L polypropylene bottles, transported to the laboratory at Virginia Tech on ice and processed within 24 h of collection. Samples were concentrated onto 0.22-μm mixed cellulose esters membrane filters (Millipore, Billerica, MA) before being fragmented with sterilized tweezers and stored at −20 °C. Filters were subject to extraction using either a FastDNA SPIN Kit or FastDNA SPIN Kit for Soil (MP Biomedicals, Solon, OH) according to the manufacturer’s instructions. DNA extracts were stored at −20 °C (short-term), or −80 °C (long-term) prior to downstream molecular analysis. No DNA extraction kit was used exclusively for all potable or nonpotable samples, thus diminishing the possibility of extraction method bias being the driver of differences observed as a function of water types.

### 16S rRNA Gene Amplicon Sequencing

Given that this was a study of archival data, there were some differences in the sequencing methodologies applied across the samples. Approximately three-quarters of the samples,^36,38,53^ (i.e., the conventional potable water, direct potable reuse water, and nonpotable reuse water) were subjected to bar-coded PCR amplification using the universal bacterial/archaeal primer set 515f/806r targeting the V4 region of the 16S rRNA gene.^54^ The remaining quarter of the samples, (i.e., the bottled water, indirect potable reuse water, and additional nonpotable reuse waters) were subjected to bar-coded PCR amplification using the modified universal bacterial/archaeal primer set 515f/926r targeting the V4-V5 region of the 16S rRNA gene.^55^ No individual set of primers were applied for sequencing all samples of a given water use category. Positive PCR products with corresponding negative control PCR reactions passing QA/QC (QA/QC was administered in multiple independent steps using gel imaging and Qubit quantification – procedures are further detailed in the Supporting Information) were Qubit quantified using the dsDNA HS-assay (Invitrogen Qubit 3 Fluorometer, Waltham, MA), before being composited with other barcoded samples at 200 to 240 ng of DNA mass per sample. Each pool of approximately 150 samples were then purified using a QIAquick PCR Purification Kit (Qiagen, Valencia, CA) before being submitted for sequencing. Sequencing was conducted at the Fralin Life Sciences Institute Genomic Sequencing Center at Virginia Tech on an Illumina MiSeq using a 250-cycle paired end protocol. Field blanks (sterilized water exposed to environmental conditions during sampling and associated sample handling), trip blanks (sterilized water exposed to only associated sample handling), filtration blanks, and DNA extraction blanks were included on each lane of sequencing and pooled using a maximum volume protocol instead of by mass.

Reads were processed using the QIIME2 pipeline (version 2020.06)^56^ and annotated using the Silva^57^ database (version 138.1). Within QIIME2, libraries were demultiplexed, trimmed to remove low quality reads, and then processed on a per lane basis using DADA2 for merging of paired end reads, denoising, chimera removal, and error correction. Generated amplicon sequence variants (ASVs) from DADA2 processing were merged from each lane of sequencing, clustered at 99%, and classified as OTUs using the Silva database. Classified OTUs were collapsed into a single OTU if they contained the same classification at the corresponding taxonomic resolution. Nontarget sequences (e.g., sequences related to mitochondria and chloroplast) were removed.

Amplicons generated by the 515f/926r primer set were trimmed to the 806r primer site after DADA2 processing, to achieve an equivalent length for data processing as the 515f/806r primer set. To achieve this, the 515f/926r ASVs generated from DADA2 were exported from the QIIME2 pipeline and processed using the Cutadapt tool.^58^ First the Cutadapt tool was run using the 806r primer with default settings, trimming ASVs from the 515f/926r set down to the 515f/806r length. To account for potential base mismatches over the 20 bp primer produced during either the DADA2 error correction or sequencing base calls, the remaining untrimmed ASVs were run through the Cutadapt tool again with a max error rate (MER) set to 0.2. After trimming, the ASVs were imported back into QIIME2 and merged with the untrimmed reads from the 515f/806r primers before continuing with the pipeline outlined above. See Supporting Information Figures 1 and 2 and Supporting Information Tables 7–10 for further details. Prior to analysis, OTUs were processed via the “decontam” package^59^ (prevalence method) to identify and remove OTUs associated with contamination found in blank samples. 1285 OTUs (out of 43 951 total OTUs, Supporting Information Spreadsheet) were identified by the “decontam” packaged and removed prior to all downstream analysis.

## Data Analysis

### Beta Diversity and Ordination

Beta-diversity metrics were generated in R and R Studio version 4.0.2^60^ (Rstudio Team, 2020) using the Phyloseq,^61^ vegan,^62^ and QIIME2r^63^ packages with outputs from QIIME2. While multiple dissimilarities and ordination methods were tested and produced highly similar trends, Bray–Curtis dissimilarities and nonmetric multidimensional scaling (NMDS) ordination are presented. Supporting Information Table 11 provides associated stress for each generated NMDS at various dimensions (*k*), with *k* = 3 selected for all plots. Visualization of plots was conducted using the ggplot2 package version 3.3.2 in R-Studio version 4.0.2.

### Core and Discriminatory Microbiome Analysis

Clustered, classified, collapsed, and filtered sequences were imported into R version 4.0.2 using QIIME2r to identify core and discriminatory taxa between different water types. Identification of core and discriminatory taxa utilized a classification-based approach, which allowed for collapsed OTUs with the same taxonomic classification before calculating an average frequency of detection using presence/absence data. Subsequent analysis primarily focused on presence/absence data, which better accounts for rare taxa.^64^ This method was conducted on each classification level from phylum to genus, with phylum and genus level analysis presented.

For the core and discriminatory analysis, the presence/absence of classified OTUs were utilized to calculate an average frequency of detection within the comparisons of interest (e.g., potable vs nonpotable waters). This average frequency of detection (i.e., percentage of samples from any given water use wherein any individual taxon was detected) was determined for each taxon within all samples from a specific water use was used to identify core and discriminatory taxa.^65,66^ Core taxa were defined as any taxonomic assignments that were detected in greater than 80% of all samples from a specific water use, while discriminatory taxa were required to meet two specific criteria: 1) any taxa that were found to be core in one water use and 2) at a frequency of detection less than 20% in the contrasting water use or if the difference in frequency of detection was greater than 60% between the two contrasted water uses. Both analyses were conducted using a modified sample set that removed atypical systems identified using the beta diversity analysis and the understanding of the systems in question. Specifically, potable conventional samples receiving a mixed source water with limited treatment (i.e., surface and groundwater) were removed to better represent more conventional drinking water systems.

Differentially abundant taxa were identified using the analysis of composition of microbiomes (ANCOM) test^67^ and utilized gene counts as input and an unmodified data set, rather than frequency of detection. ANCOM was run to compare potable (conventional and reuse) and nonpotable waters at the POC, POU, and with all samples combined.

### Statistical Analysis

All statistical analyses were conducted in R version 4.0.2 using the vegan package.^68^ Statistical differences between generated sequences, including assessing the impact of trimming, were tested using analysis of variance (ANOVA). Bray–Curtis distance matrices were tested using the analysis of similarities (ANOSIM), and permutational multivariate analysis of variance using distance matrices (PERMANOVA using Adonis2 implementation) tests (permutations = 1000). Betadisper was utilized to test for multivariate homogeneity of within group dispersions (variances) prior to ANOSIM or Adonis2 reporting. ANOSIM results were only reported after a confirmation of homogeneity within group dispersions. Adonis2 was utilized to compare the cumulative impact of multiple variables that passed the Betadisper comparison and ANOSIM testing. In this way, ANOSIM was used to test whether microbial communities were significantly different between comparisons, while Adonis2 testing was used to assess how much variation each significant variable accounted for in a combined model. *r*-stats (ANOSIM) and *R*^2^ values (ADONIS) were reported to indicate strength of the associations for statistically significant relationships following a confirmation of homogeneity, unless otherwise noted in the text.

Differential abundant taxa were identified using the analysis of composition of microbiomes (ANCOM). Alpha diversity metrics (richness, Shannon, and Simpson) were calculated for all samples. Differences of alpha diversities between groups were determined using Wilcoxon tests with *p*-value adjustment for specific comparisons of interest. A significance cutoff of *p* < 0.01 was applied for all statistical tests, with *p*-value for multiple comparisons adjusted using the conservative “BY” correction.^69^ Full statistical analyses are available in Supporting Information Tables 12 through 21.

## Results and Discussion

### Microbiomes Vary by Intended Water Use

474 samples from 5 regions of the United States, representing 7 climates, and 3 intended water uses (conventional potable [*n* = 244], potable reuse [*n* = 53], and nonpotable reuse [*n* = 177]) were collected for a study on 16S rRNA gene amplicon sequencing. Results were compiled across 3 distinct sampling campaigns to assess factors influencing microbial community dynamics. A clear distinction was found in the composition of the microbiomes as a function of the category of intended water use. Remarkably, this was the case across a very broad range of U.S. water systems, regardless of the impact imparted via various treatment trains or distribution systems, climates, or regional differences.

**Figure 1:**
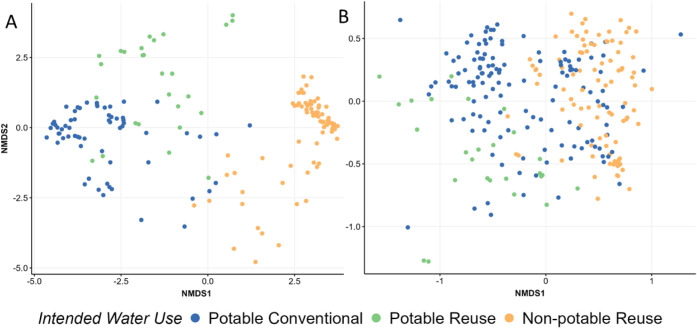
Bray–Curtis NMDS beta diversity plot for all bulk water samples at the (A) POC (*k* = 3, stress = 0.136; Betadisper *p* > 0.01; ANOSIM *p* < 0.01, *r*-stat = 0.71) and (B) POU (*k* = 3, stress = 0.159; Betadisper *p* > 0.01; ANOSIM *p* < 0.01, *r*-stat = 0.41), identified via their intended water use with overlaid correspondence analysis for phylum with an adjusted *p*-value cutoff of 0.01. Spectrum of tested water uses include potable (i.e., bottled water and conventional drinking water), potable reuse (e.g., direct potable reuse and indirect potable reuse), and nonpotable reuse (i.e., wastewater secondary effluent with various degree of tertiary treatment),^36,38,53^.

NMDS of Bray–Curtis distances, grouped by water use, are presented in Figure 1a (POC) and Figure 1b (POU), with additional NMDS plots presented in the Supporting Information for comparisons of different factors. Among all tested factors (i.e., water use classifications, climate, region, POC vs POU, residual disinfectant, sequencing primer-set, and DNA extraction kit) and when considering all samples, only the distinction between potable vs nonpotable use (Betadisper *p* > 0.01; ANOSIM *p* < 0.01, *r*-stat = 0.34) and intended water use (e.g., potable conventional, potable reuse, and nonpotable reuse) (*r*-stat = 0.38) resulted in homogeneous dispersions that were significantly associated with the composition of the microbiomes. Similarly, water use classification was the only factor that was equally effective at explaining variation for all samples from all water uses at the POU (potability (*r*-stat = 0.33) and intended water use (*r*-stat = 0.41)) and POC (intended water use (*r*-stat = 0.71)). Alpha diversity metrics for richness, Shannon diversity, and Simpson diversity were also found to be significantly different between potable (both conventional and reuse) and nonpotable waters (Wilcoxon, *p*-value < 0.01, Supporting Information Figures 3 and4). Nonpotable waters displayed higher degrees of richness, and Shannon diversity with lower Simpson diversity.

A clear difference in the microbial community composition was also identifiable at the POC as a function of intended water use category, even with a wide range of potable conventional, potable reuse and nonpotable water systems represented (ANOSIM, *r*-stat = 0.71; Figure 1a). Furthermore, the bacterial composition of potable reuse water, including both IPR and DPR, overlapped substantially with that of conventional drinking water systems. There was slightly less separation of identified microbiota according to the intended water use category at the POU (ANOSIM, *r*-stat = 0.41; Figure 1b), with much more notable overlap between some conventional potable systems and nonpotable reuse systems. This suggests that distribution systems themselves shape microbiomes in a way that leads to convergence in the composition of potable and nonpotable water microbiomes.

Various distinctions were also noted at the POC versus POU and as a function of treatments. Changes in water quality and microbial composition from POC to POU are well-documented in potable water distribution systems.^30,70−74^ Such shifts were also apparent in this study when each water use category was examined individually, especially the nonpotable reuse and minimally treated potable systems (i.e., those employing a singular process coupled with disinfection or disinfection alone, as elaborated upon in the next section) treating a mixed source water. Few prior studies have assessed shifts in microbial communities in water reuse distribution systems.^36,53,75,76^ Remarkably, the potable conventional, potable reuse, and nonpotable reuse microbiota compositions appeared to converge and become more similar at the POU relative to the POC (Supporting Information Figures 6 and 1a,b).

Several other factors were found to be statistically significant via ANOSIM when evaluating the comprehensive data set and samples subcategorized by POC or POU. However, these factors were found to be heterogeneously dispersed (Betadisper *p* < 0.01) because of either their unbalanced representation between water use (e.g., climate, region, residual disinfectant) or lack of clear clustering irrespective of water use classification (e.g., distinction between POC and POU, sequencing primers, DNA extraction kits). These visualizations (Supporting Information Figures 5–13) and statistics (Supporting Information Tables 12–14) were applied in further analyses as cofactors, but not examined further when assessing samples from multiple water uses. Therefore, of all factors tested, water use classifications were found to be the most effective at differentiating distinct microbiomes among these drinking water, wastewater, and water reuse samples collected across the US. Variation within the microbiota composition as related to additional cofactors is examined further for each water use in the following sections.

### Conventional Potable System Microbiome Drivers

**Figure 2:**
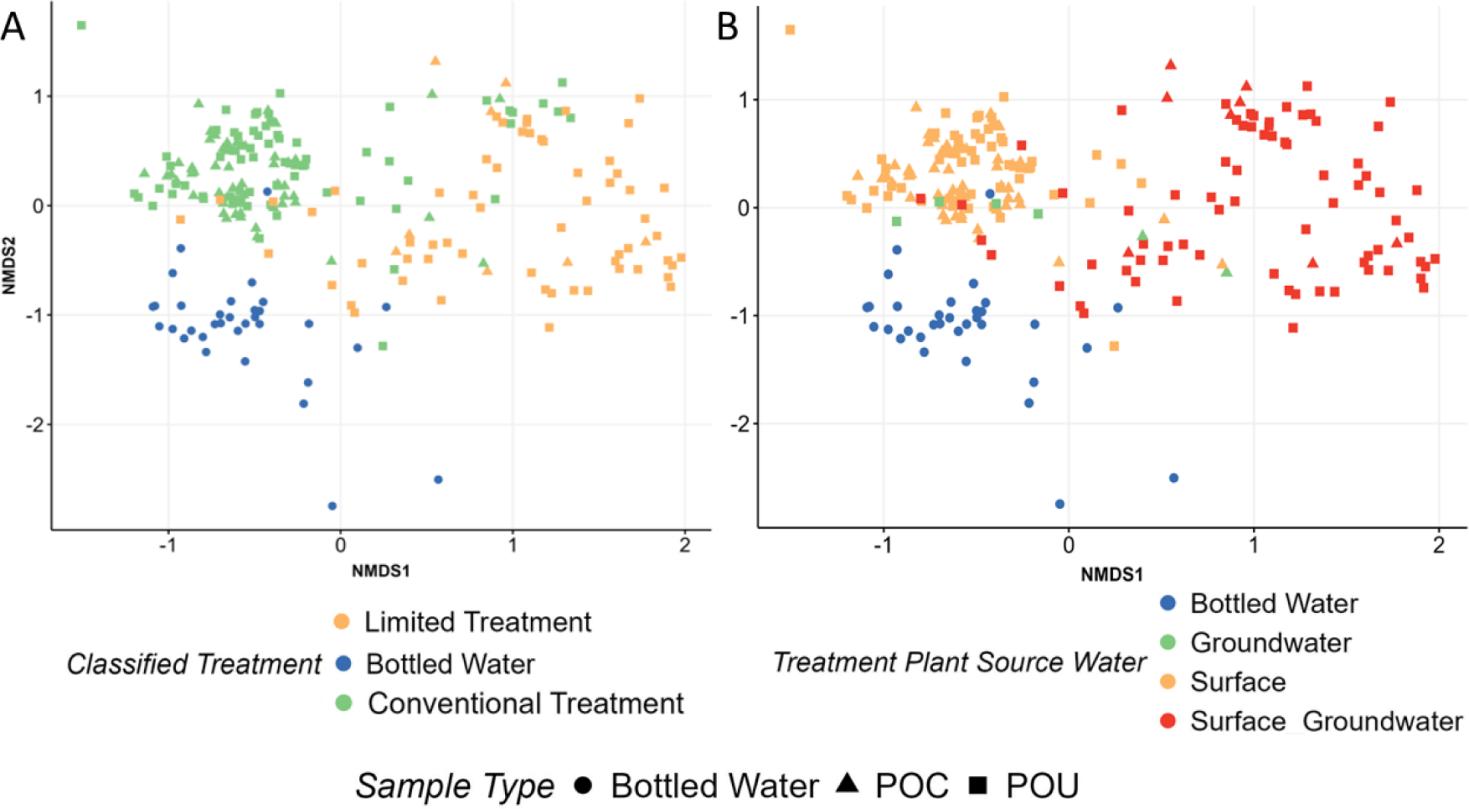
Bray–Curtis NMDS beta diversity plot for all bulk water samples intended for conventional potable use (*k* = 3, stress = 0.151). Samples are classified by their (A) respective classified treatment train (Betadisper *p* > 0.01; ANOSIM *p* < 0.01, *r*-stat = 0.61) where limited treatment was defined as 1–3 treatment processes and conventional treatment was defined as 4–7 treatment processes and (B) the source water’s origin (Betadisper *p* > 0.01; ANOSIM *p* < 0.01, *r*-stat = 0.58) where “surface groundwater” was mixed. Both figures have samples identified by their representative sampling location at either the POC or POU.

Variation encountered in the microbiota among conventional potable water samples was associated with several factors, especially treatment train configuration (Supporting Information Figure 20; *r*-stat = 0.61) and whether the origin of initial source waters was mixed (surface water and groundwater) or not (surface water or groundwater) (Supporting Information Figure 15; *r*-stat = 0.59). When assessing associations of microbiomes with treatment, dispersion was homogeneous and variation between categories were significant when accounting for the number of employed processes (Figure 2a; *r*-stat = 0.48), the disinfection residual (Supporting Information Figure 16; *r*-stat = 0.48), distinctions between the POC and POU (Supporting Information Figure 17; *r*-stat = 0.26) and treatment categorized as limited (1–3 processes) or conventional (4–7 process treatment). The source water origin was also found to be equally impactful when classified by the presence of mixing or by the source itself (Figure 2b; *r*-stat = 0.58). Furthermore, a combination of the degree of treatment and source water origin was particularly effective at differentiating the community composition (Supporting Information Figure 19; *r*-stat = 0.61) and identifying systems most susceptible to overlap with nonpotable reuse system (i.e., those with a mixed source water and limited treatment). All factors that met Betadisper and ANOSIM significance criteria were further assessed via Adnois2. From this analysis, categorical classification of treatment (Adonis2 *R*^2^ = 0.14), mixture of source waters (*R*^2^ = 0.03), source water origin (*R*^2^ = 0.01), number of employed processes (*R*^2^ = 0.10), disinfection residual (*R*^2^ = 0.02), and distinction between POC and POU (*R*^2^ = 0.01) were all found to be statistically significant (*p* < 0.01).

Minimally treated conventional potable water treatment trains resulted in microbiomes that were more similar to nonpotable reuse systems than conventional potable water systems employing more comprehensive treatments (Figure 2ab). Specifically, systems utilizing only aeration and disinfection or relying only on disinfection resulted in microbial community structures that approached or overlapped nonpotable reuse systems in bacterial composition (Supporting Information Figures 19 and 20), especially at the POU. Conversely, treatment trains that employed a combination of more conventional treatment processes; like coagulation, flocculation, sedimentation, filtration, membrane filtration, activated carbon filtration, and disinfection (e.g., chlorine, chloramine, and/or UV), were found to produce much more distinct microbiota, relative to nonpotable reuse samples and minimally treated conventional potable systems (Figures 1 and 2a and Supporting Information Figure 20). Furthermore, limited treatment was also found to experience higher measures of richness and Shannon diversity metrics compared to more conventional treatments and bottled water (Wilcoxon, *p*-value < 0.01, Supporting Information Figure 22).

Residual disinfectant was notably associated with distinct microbial communities, which is consistent with control of microbial populations being the intended purpose of application. Chloramine was also associated with higher levels of richness when compared to both bottled water and chlorine residuals (Wilcoxon, *p*-value < 0.01, Supporting Information Figure 23), speaking to its less potent disinfectant properties.

Source water was also a strong factor distinguishing among conventional potable water microbiomes. When overlaps between potable and nonpotable water microbiomes were encountered, these tended to correspond to situations where the conventional potable water treatments were minimal and potable water systems drew from a mix of groundwater and surface water sources (Supporting Information Figure 18). One possible explanation is that mixing of water sources expands the nutrient pool available to microbes, with minimal treatments accentuating such effects. Alpha diversity analysis tended to support this conclusion, as mixed source sources yielded significantly higher measures (richness, Shannon, and Simpson diversities) compared to bottled water, groundwater, and surface waters (Wilcoxon, *p*-value < 0.01, Supporting Information Figure 24). However, minimal treatment in the absence of surface water did not result in a similar microbiome as those with mixed source waters. For example, iron and manganese removal coupled with disinfection resulted in a microbial community structure very similar to conventionally treated potable water systems (Supporting Information Figure 20). Still, even with stringent treatments, source water can have lingering effects. This was illustrated by the fact that samples with more extensive treatment trains (e.g., coagulation, flocculation, sedimentation, filtration, and chlorine disinfection) when fed a mixed source water still were found to develop microbial community structures more similar to minimally treated conventional potable systems and nonpotable systems. This phenomenon was more apparent at the POU than the POC, suggesting that nutrient demand exerted in the distribution system led to population shifts with the POU also producing elevated measures of richness (Wilcoxon, *p*-value < 0.01, Supporting Information Figure 25). These findings provide new perspective on the need to tailor water treatment and distribution to the source water, especially for biologically unstable waters.^77,78^

Interestingly, climate and region were also found to be significant factors (Supporting Information Figures 21 and 26, and Supporting Information Table 15), however they failed the homogeneity criterion (Betadisper *p* < 0.01), which was likely due to their limited representation among other more dominant variables, such as treatment train configuration and/or source water origin. We hypothesize that geographical factors are likely more influential when distinguishing between systems with similar treatment and source water characteristics.

### Potable Reuse System Microbiome Drivers

Similarly to conventional potable systems, the variation within the microbiomes associated with potable reuse systems were most significantly tied to treatments applied. Namely, the entirely of the treatment train (Supporting Information Figure 29; *r*-stat = 0.60), disinfection processes employed (Supporting Information Figure 30; *r*-stat = 0.60), residual disinfectant (Supporting Information Figure 31; *r*-stat = 0.35), and the distinction between the POC and POU (Supporting Information Figure 32; *r*-stat = 0.29). Total treatment, employed disinfection processes, disinfection residual, climate, and disinfection between the POC and POU passed Betadisper and ANOSIM testing. When tested together via Adonis2, only total treatment (*R*^2^ = 0.37) and the distinction between POC and POU (*R*^2^ = 0.04) were found to be significant.

Even with substantial variety in treatment train configuration and corresponding microbiota, each potable reuse system produced a finished water with little to no overlap with nonpotable reuse waters and substantial overlap with conventional potable systems. This suggests that a wide range of potable reuse treatment options can produce waters characterized by comparable potable benchmarks. This is an encouraging finding, supporting the concept that microbiome signatures coupled with taxonomic analysis have the potential to serve as a high resolution means to categorize reuse waters as potable or nonpotable. It is also noteworthy that a clear distinction was identified between IPR and DPR systems (Supporting Information Figure 33), possibly due to the lack of residual disinfectant in the IPR effluents. However, these variables were heterogeneously dispersed making statistical interpretation difficult.

Interestingly, climate was also a notable factor that was associated with a significant difference in microbiome profiles among potable reuse samples (Supporting Information Figure 34; *r*-stat = 0.42). Given that all potable reuse systems were subjected to a high degree of treatment, this finding is consistent with the above hypothesis that at similar degrees of treatment geographical factors can act as a distinguishing factor.

### Nonpotable Reuse System Microbiome Drivers

**Figure 3:**
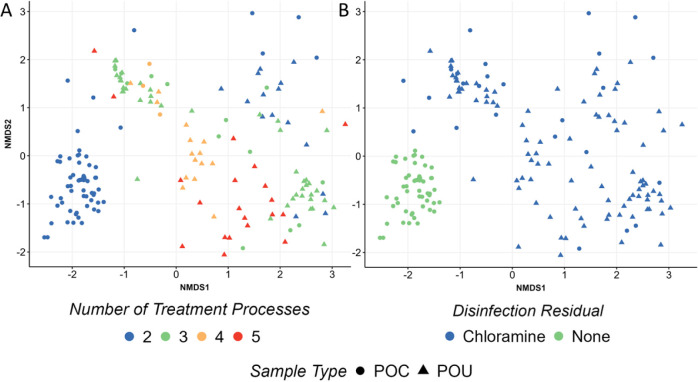
Bray–Curtis NMDS beta diversity plot for all bulk water samples intended for nonpotable use (*k* = 3, stress = 0.135). Samples are classified by their (A) number of treatment processes employed (Betadisper *p* > 0.01; ANOSIM *p* < 0.01, *r*-stat = 0.38) and (B) if a residual disinfectant was applied (Betadisper *p* < 0.01; ANOSIM *p* < 0.01, *r*-stat = 0.67). Samples are identified by their corresponding sampling location at either the POC or POU.

Microbiota in nonpotable reuse systems varied as a function of stage of treatment, both when classified categorically (Supporting Information Figure 39; *r*-stat = 0.09) and by number of treatment process (Figure 40a; *r*-stat = 0.38). There was also a significant difference in the microbiota due to the application of residual disinfection and whether the system was undisinfected versus carrying a chloramine residual (Figure 3b; Betadisper *p* < 0.01; ANOSIM *p* < 0.01, *r*-stat = 0.67), with specific to the total treatment train (Supporting Information Figure 41; Betadisper *p* < 0.01; ANOSIM *p* < 0.01, *r*-stat = 0.84), and distinctions between POC and POU (Supporting Information Figure 44; Betadisper *p* < 0.01; ANOSIM *p* < 0.01, *r*-stat = 0.67). However, the last three comparisons were found to have heterogeneous dispersions, likely due to unbalanced samplings, and should be interpreted conservatively.

Similarly to potable systems, treatment train configuration and application of disinfection were the strongest factors explaining observed variation in the nonpotable water use microbiota. Alpha diversity metrics were also significantly impacted, with more conventional treatment trains yielding lower richness and Shannon diversity compared to more limited treatments (Wilcoxon, *p*-value < 0.01, Supporting Information Figure 42). Similarly to potable reuse waters, climate was found to be a significant driver of the microbiota (Supporting Information Figure 45; *r*-stat = 0.19), especially when considering the application (or not) of disinfectants. All factors that passed Betadisper and ANOSIM testing assessed via Adnois2 identified numerical classification of treatment (*R*^2^ = 0.18) and climate (*R*^2^ = 0.06) as statistically significant (*p* < 0.01). However, the inclusion of the three categories that failed Betadisper resulted in numerical classification of treatment (*R*^2^ = 0.18), climate (*R*^2^ = 0.06), disinfection residual (*R*^2^ = 0.09), and total treatment (*R*^2^ = 0.04) being statistically significant(*p* < 0.01).

Furthermore, increased variance in microbial community composition at the POU appeared to be influenced by regional differences and changes in climate designations. This is logical, given that less stringent treatments are typically applied in the production of nonpotable reuse waters, which results in less biologically stable waters. These less biologically stable waters are subsequently primed for other factors to shape the resultant microbiota,^3,28,50−83^ such as: distribution system residence times, age, and pipe material; consumption and inactivation of microbially suppressing disinfection residuals; composition of organics; inorganics; preceding treatments; water chemistry; and other geographical differences. In the comparison between POU and POC, it was also found that all three measures of alpha diversity were significantly higher at the POU (Wilcoxon, *p*-value < 0.01, Supporting Information Figure 43).

### Phylum- and Genus-Level Core and Discriminatory Analysis

**Figure 4:**
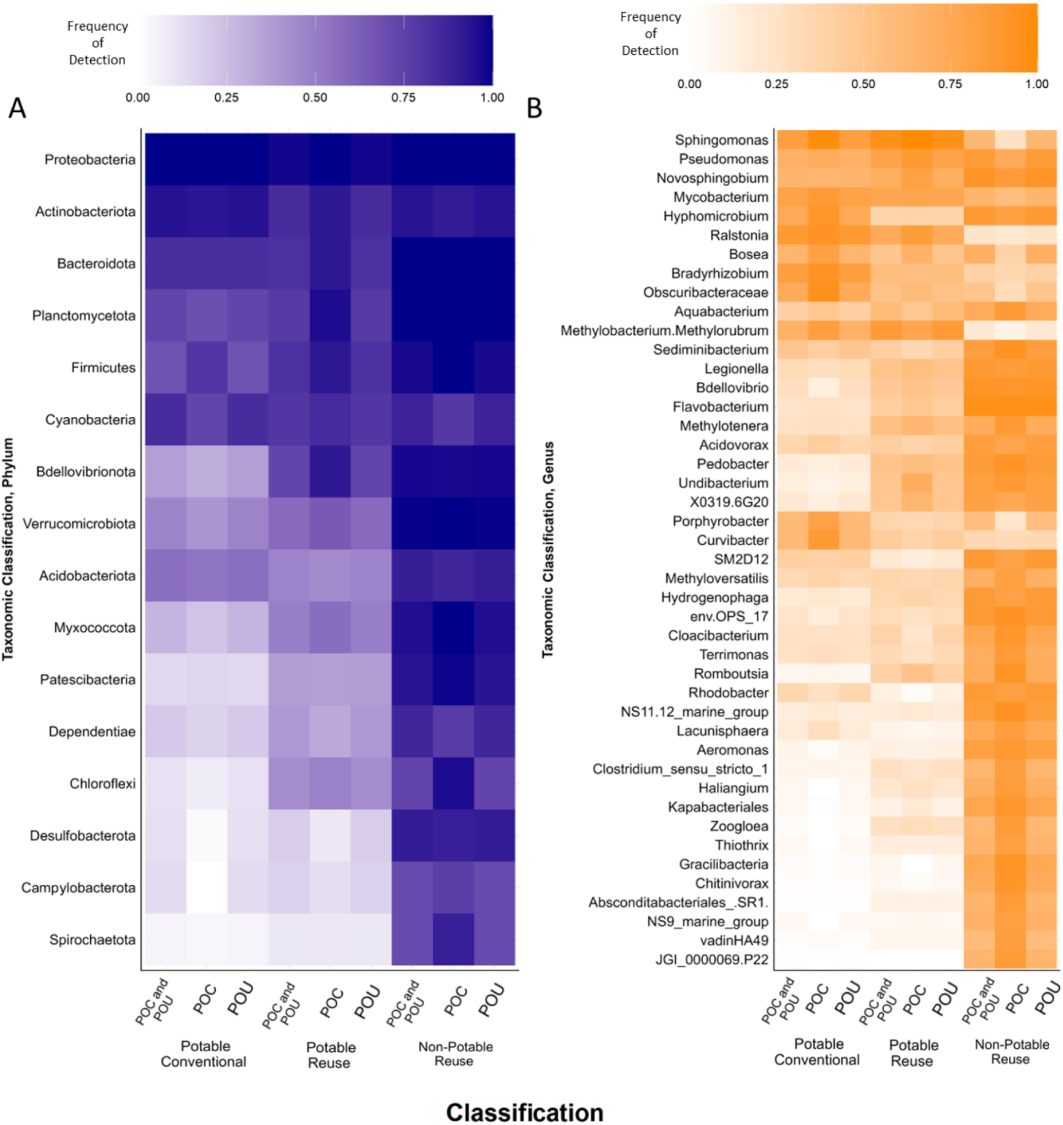
Core and discriminatory microbiome analysis conducted at the phylum (A) and genus (B) levels, grouped by water use and differentiations between the POC, POU, and all samples combined (POC and POU). Average frequency of detection among all classified samples is presented. Core taxa where any taxonomic assignments that were present in greater than 80% of all samples from that water use while discriminatory taxa were required to meet two specific criteria: 1) any OTU that was found to be core in one water use and 2) at a prevalence less than 20% in the compared water use or if the difference in prevalence was greater than 60% between the two compared water uses. Furthermore, both analyses were conducted using a modified sample set that removed atypical systems identified using the beta diversity analysis, namely potable water systems that were subjected to mixed source waters and limited treatment. Associated core and discriminatory taxa are presented in Supporting Information Tables 22 and 23. Sample sizes were: 244 potable conventional, 53 potable reuse, and 177 nonpotable reuse.

Ninety-one unique phyla were identified across all water samples, with 5, 7, and 15 of these phyla found to be core (determined via the average frequency of detection method) to potable conventional, potable reuse, and nonpotable reuse systems, respectively, at some level (e.g., all samples combined, POC, and/or POU; Figure 4a, Supporting Information Table 22). Discriminatory phyla were readily identified among the various water uses. Among water uses, more discriminatory phyla were found at the POC (17) than at the POU (5) or when evaluating the POC and POU samples together (5). At the genus level, 1924 unique genera were identified, with 10, 5, and 35 being core to potable conventional, potable reuse, and nonpotable reuse systems, respectively (Figure 4b, Supporting Information Table 23). In terms of discriminatory genera, 13 unique relationships were found when both the POC and POU samples were combined and analyzed together (Figure 4b). When assessed independently, 13 were found at the POU and 31 at the POC. Here we focus primarily on examples of taxa that most effectively distinguished the three water categories and sampling locations.

Fifty-seven phyla and 1100 genera were identified as differentially abundant between potable (conventional and reuse) and nonpotable waters at either the POC, POU, and/or all samples combined (ANCOM, *p* < 0.01; Supporting Information spreadsheet). Of these, 19 phyla and 327 genera were differentially abundant between potable and nonpotable waters at all three points of comparison (Supporting Information Spreadsheet). When accounting for log-fold change in abundances (LFC) of these 19 phyla, 9 phyla had average LFC > 1.5 (indicating nonpotable waters with higher abundances) while only 2 phyla had average LFC < -1.5 (indicating potable waters with higher abundances). At the genus level, 63 of the 327 genera were found to have average LFC > 1.5, with only 8 having LFC < -1.5.

### Key Phyla That Differentiated the Intended Water Uses

Overall, despite the low taxonomic resolution inherent to Phylum-level analysis, it was remarkable that there were significant differences between the core and discriminatory microbiomes across the water categories. In particular, regardless of distinctions between the POC and POU, Desulfobacterota exhibited high frequencies of detection in nonpotable waters and extremely low frequencies of detection in both conventional potable and potable reuse systems while Myxococcota, Patescibacteria, and Spirochaetota similarly differentiated nonpotable and conventionally potable waters. Strictly at the POC, the phylum Dependentiae also appeared to be a good candidate for differentiating nonpotable reuse waters from conventional potable waters, while Chloroflexi were identified at the POU both with higher abundances in nonpotable waters. On the other hand, Bdellovibrionota strongly differentiated conventional potable waters from both potable and nonpotable reuse waters at the POC (Figure 4a and Supporting Information Table 22). Interestingly, no taxa were identified at the phylum level via the average frequency of detection method as being core to conventional potable waters and uncommon in nonpotable waters, with only one taxon (Bdellovibrionota) being core to potable reuse water when compared to nonpotable reuse waters.

When assessing differentially abundant phyla between potable and nonpotable waters Cyanobacteria (ANCOM, *p* < 0.01, LFC = −2.06) and Actinobacteriota were found in higher abundances in potable waters (ANCOM, *p* < 0.01, LFC = −1.75). Conversely, the phyla Patescibacteria, Bdellovibrionota, Myxococcota, Bacteroidota, Verrucomicrobiota Desulfobacterota, Dependentiae, and Firmicutes where all found in higher abundances in nonpotable waters (ANCOM, *p* < 0.01, LFC < 1.5). Of these, Desulfobacterota, Patescibacteria, and Myxococcota make the best candidates for discriminatory phyla between potable (conventional and reuse) and nonpotable waters as they were consistently identified via both the average frequency and detection (presence/absence) and differentially abundant (abundance) methods (Table 1). Interestingly, a recently published study applied a similar methodology to test the potability of an advanced treatment train and determined that in this specific instance the water quality was not suitable for drinking water, based on high concentrations of *E. coli* and total coliforms.^84^ They also found that 7 of the 8 discriminatory phyla (Patescibacteria, Bdellovibrionota, Myxococcota, Bacteroidota, Verrucomicrobiota, Dependentiae, and Firmicutes) identified in this study were among the 15 most abundant at the end of treatment in their study.^84^

**Table 1:** Selected Results of the ANCOM Differentially Abundant Test on Potable vs Non-Potable Waters at the Phylum level^a^

Potable vs Non-Potable	POC and POU		POC		POU			
Phylum^b^	Adjusted *p*-value	LFC	Adjusted *p*-value	LFC	Adjus ted *p*-value	LFC^c^	Average A LFC	Discriminatory Frequency of Detection^d^
Patescibacteria	4.5E-55	3.49	1.1E-76	5.29	8.8E-15	2.43	3.74	**-**
Bdellovibrionota	8.8E-41	2.74	1.2E-20	3.08	2.0E-31	3.07	2.96	POC
Myxococcota	2.4E-34	2.24	1.3E-48	3.76	8.7E-08	1.43	2.48	POC and POU
Bacteroidota	2.5E-29	2.23	1.9E-13	2.40	2.5E-16	2.30	2.31	-
Verrucomicrobiota	2.0E-28	2.25	1.6E-08	2.03	1.3E-18	2.37	2.21	-
SAR324_clade(Marine_group_B)	1.6E-32	2.01	8.5E-20	2.03	7.1E-29	2.53	2.19	-
Desulfobacterota	4.3E-24	1.86	1.3E-32	2.80	3.0E-08	1.54	2.07	POC and POU
Dependentiae	1.3E-20	1.91	3.1E-04	1.44	5.5E-23	2.49	1.95	POC and POU
Firmicutes	8.0E-12	1.52	1.2E-14	2.39	5.4E-05	1.44	1.78	-
Fusobacteriota	1.2E-07	1.21	2.5E-08	1.69	1.8E-04	1.35	1.42	-
Actinobacteriota	1.7E-09	–1.63	1.6E-10	–2.54	9.4E-03	–1.08	–1.75	-
Cyanobacteria	7.9E-11	–1.72	8.3E-19	–3.45	3.1E-03	–1.02	–2.06	-

a Positive LFC indicate higher abundance in non-potable waters while negative LFC indicate higher abundance in potable waters.

b Differentially abundant was defined as ANCOM, *p* < 0.01, for all three comparison at the POC, POU, or both POC POU.

c Taxa included in this table were selected to highlight those with the greatest magnitude of average log-fold change (LFC). The full table of differentially abundant taxa determined via ANCOM are provided in the Supporting Information spreadsheet.

d Taxa that were also discriminatory based on average frequency of detection.

When further examining phyla that discriminated nonpotable reuse waters from potable waters (conventional and reuse), most have been previously associated with wastewater treatment plants. Myxococcota and Bdellovibrionota are known for their predatory or parasitic behaviors in wastewater treatment plants^85,86^ while Desulfobacterota include numerous organisms capable of reducing sulfur compounds via the DsrAB-dissimilatory sulfite reduction pathway^87^ and has been identified in high abundances during aerobic^88^ and anerobic^89^ wastewater treatment and not detected in two studies focused on potable reuse trains.^76,90^ The phylum Spirochaetota has been identified to increase abundance in sewer biofilms when exposed to higher concentrations of prescription drugs^91^ and beneficial to methanogenesis pathways.^92^ Chloroflexi are commonly found in treatment plants designed to remove phosphorus and nitrogen, with the ability to contribute to floc formation, degrade complex polymeric organic compounds, and sometimes cause sludge bulking.^93,94^ Furthermore, Chloroflexi was found to be enriched in soils irrigated with varying degrees of treated wastewater, with the highest degree of enrichment occurring with less treated water.^95^ However, it is important to note that phylum is a very broad taxonomic classification that encompasses numerous diverse functions.

### Key Genera That Differentiated the Water Uses

Of all highly detected genera, only *Sphingomonas* was detected to be core to potable reuse waters at the POC, POU, and with all samples combined while only the genera *Sphingomonas*, *Ralstonia*, and *Bradyrhizobium* met this criterion for potable conventional waters. Meanwhile, *Novosphingobium*, *Hyphomicrobium*, *Sediminibacterium*, *Legionella*, *Bdellovibrio*, *Flavobacterium*, *Pedobacter*, *UndibacteriumSM2D12*, *env.OPS_17*, *Hydrogenophaga*, *Rhodobacter*, *NS11.12_marine_group*, and *Aeromonas* were detected as core genera associated with all stages of nonpotable waters.

Unlike at the phylum level, discriminatory genera core to potable conventional waters (and to a slightly lesser degree potable reuse waters) were detected. Namely, *Ralstonia* at all comparisons and *Methylobacterium*.*Methylorubrum*, *Sphingomonas*, and *Obscuribacteraceae* at either the POC or POU.

When considering genera core to nonpotable systems and uncommon in conventional potable and potable reuse systems 2 genera (*NS11.12_marine_group* and *Aeromonas*) were found to be consistently discriminatory throughout all three comparisons, regardless of their origin relative to the POC or POU. Additionally, 6 genera (*Bdellovibrio*, *Flavobacterium*, *Pedobacter*, *Undibacterium*, *env.OPS_17*, and *Hydrogenophaga*) were found to be discriminatory between nonpotable and conventional potable systems with another 2 (*SM2D12* and *Rhodobacter*) differentiating nonpotable and potable reuse waters.

Of the differentially abundant taxa identified consistently between potable and nonpotable water (ANCOM, *p* < 0.01) and across all three comparisons (POC, POU, and all samples combined), the 6 genera with the largest negative LFC (Table 2) included *Obscuribacteraceae* (ANCOM, LFC = −2.77), *Mycobacterium* (ANCOM, LFC = −2.92), *Bradyrhizobium* (ANCOM, LFC = −3.19), *Sphingomonas* (ANCOM, LFC = −3.58), *Methylobacterium-Methylorubrum* (ANCOM, LFC = −3.65), and *Ralstonia* (ANCOM, LFC = −4.09). Four of the six were identified as being discriminatory taxa via the average frequency of detection criteria.

**Table 2:** Selected Results of the ANCOM Differentially Abundant Test on Potable vs Non-Potable Waters at the Genus level^a^

Potable vs Non-Potable	POC and POU		POC		POU			
Genus^b^	Adjusted *p*-value	LFC	Adjus ted *p*-value	LFC	Adjusted *p*-value	LFC	Average LFC^c^	Dis criminatory Frequency of Detection^d^
*Flavobacterium*	7.9E-119	4.79	3.1E-54	4.71	2.0E-53	4.71	4.74	POC and POU
*Aeromonas*	4.6E-63	3.55	8.6E-42	3.59	3.4E-27	3.54	3.56	POC and POU
*Chitinivorax*	4.2E-58	3.29	0.0E+00	4.31	6.3E-15	2.45	3.35	POC
*Hydrogenophaga*	1.2E-46	3.37	3.0E-09	2.39	4.5E-40	4.02	3.26	POC and POU
*Gracilibacteria*	3.9E-45	3.13	0.0E+00	4.96	1.4E-07	1.61	3.24	POC
*NS11.12_marine_group*	1.4E-51	3.21	7.3E-29	3.47	8.9E-19	2.88	3.19	POC and POU
*Zoogloea*	7.5E-35	3.03	1.9E-50	4.65	2.8E-07	1.64	3.11	POC
*Rheinheimera*	3.1E-30	2.89	1.6E-05	1.60	2.9E-38	3.99	2.83	-
*Undibacterium*	UE-30	2.98	1.3E-05	2.03	7.8E-19	3.44	2.82	POC and POU
*Romboutsia*	2.7E-43	2.78	2.0E-45	3.59	2.2E-11	2.00	2.79	POC
*Saccharimonadales*	1.3E-34	2.68	0.0E+00	3.58	1.9E-11	2.05	2.77	-
*Pedobacter*	3.6E-36	2.81	4.9E-14	2.92	2.5E-16	2.51	2.75	POC and POU
*Candidatus* Megaira	1.1E-47	2.72	6.6E-15	2.22	2.4E-44	3.21	2.72	-
*env.OPS_17*	4.8E-36	2.46	6.2E-45	3.55	7.8E-09	1.78	2.60	POC and POU
*Bdellovibrio*	3.8E-38	2.48	2.2E-14	2.18	6.6E-28	2.91	2.52	POC and POU
*Rhodobacter*	4.3E-24	2.55	2.2E-12	2.52	2.6E-11	2.46	2.51	POC and POU
*Obscuribacteraceae*	6.8E-16	–2.31	2.4E-24	–4.49	4.2E-04	–1.50	–2.77	POC
*Mycobacterium*	2.2E-15	–2.53	1.1E-13	–3.77	2.2E-08	–2.46	–2.92	-
*Bradyrhizobium*	9.1E-29	–2.98	4.0E-22	–4.19	1.5E-09	–2.41	–3.19	-
*Sphingomonas*	7.4E-21	–2.98	3.9E-82	–6.33	1.4E-03	–1.43	–3.58	POC
*Methylobacterium-Methylorubrum*	2.1E-49	–3.40	4.3E-36	–4.20	1.3E-25	–3.35	–3.65	POC and POU
*Ralstonia*	6.3E-64	–4.07	1.5E-33	–4.60	4.9E-29	–3.60	–4.09	POC and POU

a Positive LFC indicate higher abundance in non-potable waters while negative LFC indicate higher abundance in potable waters.

b Differentially abundant was defined as ANCOM, *p* < 0.01, for all three comparison at the POC, POU, or both POC POU.

c Taxa included in this table were selected to highlight those with the greatest magnitude of average log-fold change (LFC). The full table of differentially abundant taxa determined via ANCOM are provided in the Supporting Information spreadsheet.

d Taxa that were also discriminatory based on average frequency of detection.

Of the 10 genera that were identified as being core to nonpotable waters and discriminatory to either all potable waters or conventional/reuse potable waters using average frequency of detection, all were found to display consistently higher abundances in nonpotable waters (ANCOM, *p* < 0.01) with LFC > 1.5. Furthermore, 8 genera were found to be within the top 15 highest average LFC (Table 2) for differential abundant taxa consistently identified between all three comparisons, including: *Flavobacterium* (ANCOM, LFC = 4.74), *Aeromonas* (ANCOM, LFC = 3.56), *Hydrogenophaga* (ANCOM, LFC = 3.26), *NS11–12_marine_group* (ANCOM, LFC = 3.19), *Undibacterium* (ANCOM, LFC = 2.82), *Pedobacter* (ANCOM, LFC = 2.75), *env.OPS_17* (ANCOM, LFC = 2.6), and *Bdellovibrio* (ANCOM, LFC = 2.52).

When considering all three water uses, a few discriminatory genera are known to contain human pathogenic members including *Ralstonia*, *Mycobacteria*, and *Aeromonas* with both *Ralstonia* and *Mycobacteria being more abundant in potable waters.Mycobacterium* spp. are known for slow growth rates, preference for biofilms, and high disinfection tolerance.^96^*Aeromonas* are known as an important disease-causing pathogen for fish and other cold-blood species while being an opportunistic pathogen in humans.^97^ In one study focused on drinking water and wastewater treatment plants, *Aeromonas* was found in wastewater and ozonated effluents, but not in ground waters, tap waters, or chlorination tanks.^98^ Among *Ralstonia* spp., which wasthe only genus containing human pathogens that was core to potable systems, some are known denitrifiers and opportunitic pathogens.^99,100^

Beyond human pathogens, *Pedobacter*, *Bdellovibrio*, *Hydrogenophaga*, and *Flavobacterium* were found to be discriminatory between potable waters and nonpotable waters with higher abundances in nonpotable waters. While not a known human pathogen, *Pedobacter* has been noted to carry a particularly high number of antibiotic resistance genes^101^ and to be enriched in hospital wastewater with high pharmaceutical concentrations.^102^*Bdellovibrio* is parasitic to Gram negative bacteria^103^ and was found in treatment trains relying on biological treatment (core to nonpotable reuse and not uncommon in potable reuse water), where there are abundant bacteria to prey upon. *Flavobacterium* is widely encountered in aquatic environments, especially wastewater treatment plants, and has the potential to cause disease in fish populations while also contributing to the degradation of complex organic matter.^104−106^*Hydrogenophaga* has been identified as a facultative autotrophic denitrifier in wastewater systems^107^ and enriched in distribution systems transitioning from conventional to DPR waters^76^ making it effective at differentiating between conventional potable, potable reuse, and nonpotable reuse waters.

Of all discriminatory relationships identified via multiple methods, the genera *Aeromonas*, and *NS11.12_marine_group* were identified as the most suitable candidates for distinguishing potable and nonpotable waters, as they were all consistently detected and abundant in nonpotable reuse systems at the POC, persist at high frequency of detection throughout nonpotable reuse distribution systems, and are uncommon at the POC and POU in both conventional potable systems and reuse systems intended for potable use. When comparing just conventional potable waters and nonpotable waters *Hydrogenophaga*, *Bdellovibrio*, *Flavobacterium*, *env.OPS_17,Pedobacter*, and *Undibacterium* are suitable candidates for core taxa to nonpotable systems, while *Ralstonia* was similarly effective with higher abundances within conventional potable systems.

### Effects of Water Distribution on Core and Discriminatory Genera: POC Vs POU

It is important to consider that substantial shifts in water quality occur during distribution from the POC to the POU, which is where there is actual potential for human exposure. Overall, regardless of the level of taxonomic classification, more discriminatory relationships were found directly after treatment than samples that were subjected to distribution (Figure 4 and Supporting Information Tables 22 and 23), suggesting that distribution systems act to normalize the microbial composition of waters originally treated with different intended uses. Both genus and phylum based-discriminatory analysis revealed that most core taxa were associated with nonpotable samples at the POC. Subsequent distribution led to increased microbial variation and shifts in microbial community composition that are likely related to localized factors.

Nonpotable reuse systems were predominately found to contain more core taxa and typically made up the core component of the discriminatory analysis, though other discriminatory relationships were found. Potable conventional systems and potable reuse systems also yielded extremely limited discriminatory components and shared many core taxa, while simultaneously being divergent from nonpotable waters. This supports the overall hypothesis that intended water use, and the corresponding factors, such as treatment technologies applied, select for distinct microbiota.

### Next Steps

This study demonstrated that 16S rRNA gene amplicon sequencing can effectively discriminate waters based on their intended use/reuse application. Comparison of beta diversities and associated taxa could provide a comprehensive and high resolution means to assess water quality and its overall potability that can complement existing assessments focused on fecal indicators. This can be of particular value given concerns that fecal indicator paradigms can fall short in water reuse contexts.^108^ In these cases, higher resolution assessments can provide value in characterizing the impact of novel treatment trains, characterizing impacts of changing source waters on microbial composition, and diagnosing systems experiencing operational challenges. We additionally identified a number of candidate phyla and genera that were especially discriminatory among the water types that can be considered in future studies aimed at refining the approach demonstrated herein. Such an approach could especially be of value when seeking to evaluate novel water treatment technologies and where on the spectrum of potable to nonpotable signatures the resulting microbiomes lie. Overall, improved understanding of the microbiomes associated with various water qualities can help better support and optimize a “fit for purpose” paradigm of water reuse.

## Supporting Information Available

Additional descriptions of the experimental details and the methodologies applied, including read processing information and sample metadata; complete statistical testing for ANOVA, ANISOM, ADONIS, BetaDisper, ANCOM testing, and more complete reporting on core and discriminatory taxa at the phylum and genus levels are presented; supplemental alpha and beta diversity figures related to trends within categorical variables (e.g., water use, climate, region, disinfectant, treatments, and sample type) (PDF)
