# Supplementary material for: What is the Difference between Conventional Drinking Water, Potable Reuse Water, and Nonpotable Reuse Water? A Microbiome Perspective
Source: Environ Sci Technol. 2024 Sep 11;58(38):16877–90. doi: 10.1021/acs.est.4c04679 (PMC11428167; doi:10.1021/acs.est.4c04679)
Supplement: Supplementary file 1 — es4c04679_si_001.pdf [file es4c04679_si_001.pdf]

Supporting Information

Title: What is the Difference between Conventional Drinking Water, Potable Reuse Water, and Non-potable Reuse Water? A Microbiome Perspective

Authors: Matthew F. Blair[1], Emily Garner[2], Pan Ji[1], and Amy Pruden[1]*

[1]Via Department of Civil and Environmental Engineering, Virginia Tech, Blacksburg, Virginia 24061, United States

[2]Wadsworth Department of Civil and Environmental Engineering, West Virginia University, Morgantown, West Virginia 26506, United States

*Corresponding Author (e-mail: apruden@vt.edu)

Number of pages: 64

Number of tables: 23

Number of figures: 46

<u>Supporting Materials and Methods</u>

<u>Summary of Sample Metadata</u>

SI Table 1. Key Attributes of Water Systems and Samples Included in this Study. Different metadata classification are broken up by attribute and intended water use at either the POC, POU, or combined.

Comparison	Attributes	Potable			Potable Reuse			Non-Potable Reuse			Totals
		POU (n)	POC (n)	All (n)	POU (n)	POC (n)	All (n)	POU (n)	POC (n)	All (n)	
Water Use		132	63	244*	26	27	53	95	82	177	474
Climate	BSh – hot semi-arid	7	2	9				16	3	19	28
	BWk – cold desert	2	1	3	8	4	12				15
	Cfa – humid subtropical	56	22	78		15	15	39	68	107	200
	Csa – hot-summer Mediterranean	4	2	6	6	2	8				14
	Csb – warm-summer Mediterranean	33	6	39	12	6	18	40	11	51	108
	Dfa – hot-summer humid continental	10	10	20							20
	Dfb – warm-summer humid continental	20	20	40							40
Region	Midwest	30	30	60							60
	Northeast	10	10	20							20
	Southeast	46	12	58		15	15	39	68	107	180
	Southwest	23	4	27				36	11	47	74
	West	23	7	30	26	12	38	20	3	23	91
Disinfection Residual Type	NH_2Cl	54	16	70	18	8	26	95	26	121	217
	Cl_2	78	47	125	8	4	12				137
	None					15	15		56	56	71
DNA Extraction Kit	FastDNA Spin Kit	132	63	244	26	12	38	95	26	121	354
	FastDNA Spin Kit for Soil					15	15		56	56	71
Source Water	Groundwater	4	2	6	8	3	11				17
	Surface Water	56	53	109	18	9	27				136
	Surface and Groundwater	72	8	80							80
	Treated Wastewater					15	15	95	82	177	192

Disinfection Processes	**NH_2Cl**	38	3	41				16	3	19	60
	**NH_2Cl O_3 Industry**				2	1	3				3
	**NH_2Cl O_3 UV**					15	15				15
	**NH2Cl UV**	10	10	20	2		2		4	4	26
	**Cl_2**	54	36	90	4	2	6				96
	**Cl_2 NH_2Cl**							59	19	78	78
	**Cl_2 NH_2Cl | UV NH_2Cl****							20		20	20
	**Cl_2 NH_2Cl O_3**	2	1	3							3
	**Cl_2 NH_2Cl O_3 UV**				2	1	3				3
	**Cl_2 ClO_2**	16	2	18							18
	**Cl_2 O_3**	2	1	3	6	3	9				12
	**Cl_2 O_3 UV**				2		2				2
	**Cl2 UV**	10	10	20	4	2	6				26
	**Cl_2 UV Pasteurization**				2	1	3				3
	**UV**				2	2	4				4
Treatment Processes	**Aeration Cl_2**	36	2	38							38
	**Bardenpho Cl_2**							16	10	26	26
	**Bardenpho Cl_2 | Biofiltration IFAS UV ****							20		20	20
	**Biofiltration IFAS UV**								4	4	4
	**CAS Denit**								56	56	56
	**CAS Denit Cl_2**							23	6	29	29
	**CAS Denit Coag Floc Sed O_3 BAC GAC UV**					15	15				15
	**CAS DualMediaFiltration Cl_2**							20	3	23	23
	**CAS DualMediaFiltration|MBR NH_2Cl ****							16	3	19	19
	**CAS MF RO UV**				2	2	4				4
	**CAS MF RO UV O_3 Coag Floc Sed Filtration Cl_2**				2		2				2
	**CAS Nit PDenit BioP Coag Floc Sed Filtration Cl_2**				4	2	6				6
	**CAS O_3 BAF Coag Floc Sed Filtration Cl_2**				4	2	6				6
	**CAS O_3 BAF O_3 Coag Floc Sed Filtration Cl_2**				2	1	3				3
	**Cl_2**	20	4	24							24
	**Coag Floc Sed Filtration Cl_2**	18	3	21							21

	**Coag Sed Filtration Cl_2**	20	20	40							40
	**Coag Sed Filtration GAC UV Cl_2**	10	10	20							20
	**Coag Sed Filtration UF Cl_2**	10	10	20							20
	**Coag Sed Filtration UV NH_2Cl**	10	10	20							20
	**FE&MN Cl_2**	2	1	3							3
	**FE&MN Cl_2 CAS MF RO UV Cl_2**				4	2	6				6
	**FE&MN Cl_2 CAS UF RO UV Past Cl_2**				2	1	3				3
	**NH_2Cl**	2	1	3							3
	**NH_2Cl CAS MF RO UV**				2		2				2
	**O_3 Coag Filtration NH_2Cl O_3 Coag Floc Sed Filtration Cl_2**	2	1	3							3
	**O_3 Coag Filtration NH_2Cl CAS MF RO UV O_3 Coag Floc Sed Filtration Cl_2**				2	1	3				3
	**O_3 Coag Filtration NH_2Cl Industry**				2	1	3				3
	**O_3 Coag Floc Sed Filtration Cl_2**	2	1	3							3
**Reuse Blending Ratio**	**0.05**				2	1	3				3
	**0.1**				16	7	23				23
	**0.5**				8	4	12				12
	**None**	132	63	244*	0	15	15	95	82	177	387

* Includes 49 bottled water samples ** Indicates mixing of two treatment trains

Köppen classification: Bsh – midlatitude steppe and desert; Bwk – cold desert; Cfa – humid subtropical; Csa hot-summer Mediterranean; Csb – warm-summer Mediterranean; Dfa – hot-summer humid continental; Dfb – warm-summer humid continental

SI Table 2: Summary of sample counts for each water us/type and sample location.

Sample Type	Blank	Bottled Water	POC	POU	Grand Total
**Blank**	30	-	-	-	30
**Non-potable Reuse**	-	-	82	95	177
**Potable Conventional**	-	49	63	132	244
**Potable Reuse**	-	-	27	26	53
**Grand Total**	30	49	172	253	504

SI Table 3: Summary of Climate metadata based on intended water use using Koppen classifications.

Köppen Climate Classification	Description	Potable	Reclaimed	Reuse	Totals
**Bsh**	Mid-latitude steppe and desert climate	9	19	0	28
**Bwk**	Cold desert climate	3	0	12	15
**Cfa**	Humid subtropical climate	78	107	15	200
**Csa**	Hot-summer Mediterranean climate	6	0	8	14
**Csb**	Warm-summer Mediterranean climate	39	51	18	108
**Dfa**	Hot-summer humid continental climate	20	0	0	20
**Dfb**	Warm-summer humid continental climate	40	0	0	40
	Total	195	177	53	425

SI Table 4. Summary of disinfection processes based on intended water use. This included any treatment process commonly associated with disinfection at any stage of treatment.

Disinfection Treatments	Potable	Reclaimed	Reuse	Totals
**Chloramine**	41	19	0	60
**Chlorine**	90	0	6	96
**Chlorine Chloramine**	0	98	0	98
**Chlorine ChlorineDioxide**	18	0	0	18
**Ozone Chloramine Industry**	0	0	3	3
**Ozone Chlorine**	3	0	9	12
**Ozone Chlorine Chloramine**	3	0	0	3
**Ozone UV Chloramine**	0	0	15	15
**Ozone UV Chlorine**	0	0	2	2
**Ozone UV Chlorine Chloramine**	0	0	3	3
**UV**	0	0	4	4
**UV Chloramine**	20	4	2	26
**UV Chlorine**	20	0	6	26
**UV Chlorine Pasteurization**	0	0	3	3

SI Table 5. Summary of disinfectant residuals based on intended water us.

Disinfection Residual	Potable	Reclaimed	Reuse	
**Chloramine**	70	121	26	217
**Chlorine**	125	0	12	137

SI Table 6: Summary of POC vs POU samples based on intended water use.

Sample Location	Potable	Reclaimed	Reuse
**Point of Compliance, POC (WTP)**	63	82	27
**Point of Use, POU (DS)**	132	95	26

QA/QC of Sequencing Library Prep

All samples prepped in lab (both in prior studies and updates samples) were subjected to the same library prep pipelines and QA/QC standards regardless of user or sequencing protocol. Specifically, the QA/QC during library prep included prepping four amplicons per sample (3 positive amplicons with sample DNA and 1 negative control without sample DNA). All amplicons were subjected to each stage of library prep and identical workflows to identify potential contamination. Amplicons were initially screened for successful amplification using gel imaging where the negative controls were also checked to insure no unintended amplification. Any samples with amplification of their negative controls were rerun from the start of the protocol. All samples passing this stage of QA/QC achieved either (1) successful amplification of positive samples without amplification within the negative control (all environmental samples) or (2) unsuccessful amplification in both sample and negative control (blanks) after at least 2 attempts. Note, all blank samples failed this step of QA/QC due to low initial DNA mass, but were still included in the sample set regardless. Following QA/QC associated with gel imaging, samples were quantified using Qubit to calculate amplified concentrations and prep samples for an equal mass pooling. This stage also identified samples for reruns if (1) triplicate DNA concentrations experienced significant variability or (2) negative controls experienced comparable DNA concentrations to low mass samples even without significant identifiable contamination during previous QA/QC. After this round of QA/QC, samples were pooled at an

equal mass per lane. Again, the sample blanks experienced low DNA concentrations and failed this stage of QA/QC, but were spiked at the highest volume possible (~65uL).

Validation of Post-Sequencing Trimming

One of the first objectives of this work was to validate consolidated analysis of 16S rRNA gene amplicon sequences originating from different primers with an overlapped sequencing region. As NGS technologies continue to gain popularity and be applied throughout the water and wastewater industries it is inevitable that variation will be introduced throughout all stages of sequencing without an agreed upon standard. Variation present among downstream analysis is the least worrisome since results can always be reproduced in accordance with agreed upon methods as standards become adopted industry-wide. However, variability present prior to sequencing (e.g. within DNA extraction, library prep, and sequencing pipelines) have a much greater potential to introduce variation that cannot be accounted for following the completion of sequencing. Therefore, to increase the longevity and usability of sequencing results and allow for an increase in industry wide collaborative studies, there is a need to be able to develop and validate techniques to overcome minor differences in preparatory pipelines prior to sequencing. One such case relates to the variability associated with differing targeted regions of the 16S rRNA gene utilized in 16Sco rRNA gene amplicon sequencing.

Here sequencing results from two commonly targeted regions (V4 and V4-V5) (with an overlapped amplicon region) were successfully combined by trimming produced sequence variants down to an identified primer binding site using the cut-adapt tool. SI Figure 1 graphically presents the distribution of base pairs (bps) lengths for each generated ASV, before and after multiple attempts at trimming. The control samples represent a lane of untrimmed sequences produced from the 515f-926r primer set targeting both the V4-V5 region, while the

combined samples represent the ideal targeted range of ASV lengths when only using the V4 region and 515f-806r primer set. Similarly, Figure S2 provides the distribution of removed bps, measured via the change in sequence length, during multiple trimming attempts. Trims around 120 bps were ideal as the 515f-926r primer set targets a region that is roughly 120 bps longer than the 515f-806r primer set. However, it is worth noting that various organisms contain nucleotide variation throughout the variable region(s) of the 16S rRNA gene indicating that all sequences will not have exactly the same ASV length nor the same change in length. Instead a reasonably tight distribution about the 120-bp trim was expected. Changes in ASV length much greater than 120 bps or much lower than 120 bps indicated too lenient or strict trimming parameters, respectively.

With this context understood, SI Figure 1 and 2 support the ability for the Cutadapt tool to be utilized ex post facto, with optimized trimming parameters. Trimming was effective at retroactively reducing 515f-926r generated ASVs down to the 806r binding site, consolidating all generated sequences to the same V4 region of the 16S rRNA gene. Changes in the Cutadapt MER were seen to have the greatest impact on the effectiveness of trimming. Increased MERs lead to decreased binding site specificity and excessive trimming. Conversely, a lower max error rate increased primer site specifically and lead to more accurate trims with resultant ASVs falling within the desirable trimming ranges. However, lower MERs also lead to a higher percentage of untrimmed sequences. Therefore, a tiered trimming approach was found to be most effective, where sequences were initially trimmed with a low max error rate (MER=0.1) and then untrimmed sequences were re-trimmed with a higher max error rate (MER=0.2). The resultant sequences from the tiered approach proved to be the only resultant sequences tested that did not have sequence length that were statistically different from the targeted lengths (ANOVA, p-value

= 0.987) with all other trimming approaches (MER = 0.1, 0.4, 0.6) generating statistically different length distributions (ANOVA, p-value < 0.05), SI Table 7. Further, all tested trimming parameters generated statistically different change in length distributions (ANOVA, p-value < 0.05) indicating that the Cutadapt tool's MER parameter was an effective means of controlling trimming site specificity, SI Table 8. The tiered approach with final MER=0.2 resulted in 99.32% of all sequencing being trimmed correctly, 0.38% remaining untrimmed, and 0.30% being poorly trimmed. This compared favorably to 96.98% correctly trimmed, 2.32% untrimmed, and 0.70% poorly trimmed for the default Cutadapt parameters, SI Tables 9 and 10. Trimming validation asserts that post sequencing trimming is a reasonable and effective approach to consolidating 16S rRNA gene amplicon sequences originating from different primers with an overlapped sequencing region into a single region of interest, prior to downstream analysis.

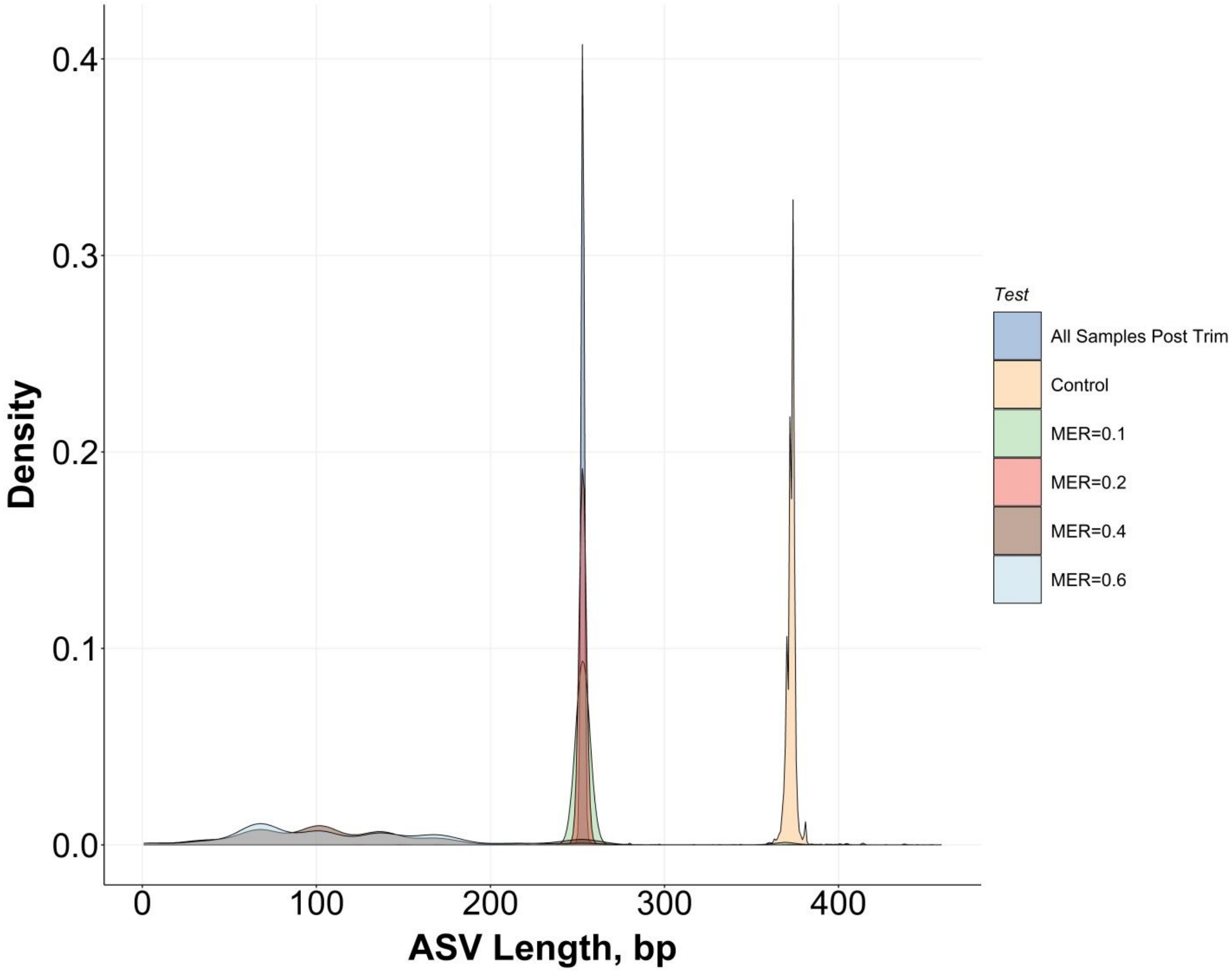

SI Figure 1. Density plot of 16S rRNA gene amplicon ASV lengths after multiple Cutadapt pipelines trimmed ASVs generated from the 515f/926r down to the 515f/806r binding site. Here the control density represents untrimmed 515f/826r ASV lengths, all samples post trim represent the model 515f/806r reference density, MER=0.1 represents the default Cutadapt parameters, and MER=0.2, MER=0.4, and MER=0.6 represents a tiered trimming approach with the secondary trim MER set to their respective values after an initial trim at the default parameters.

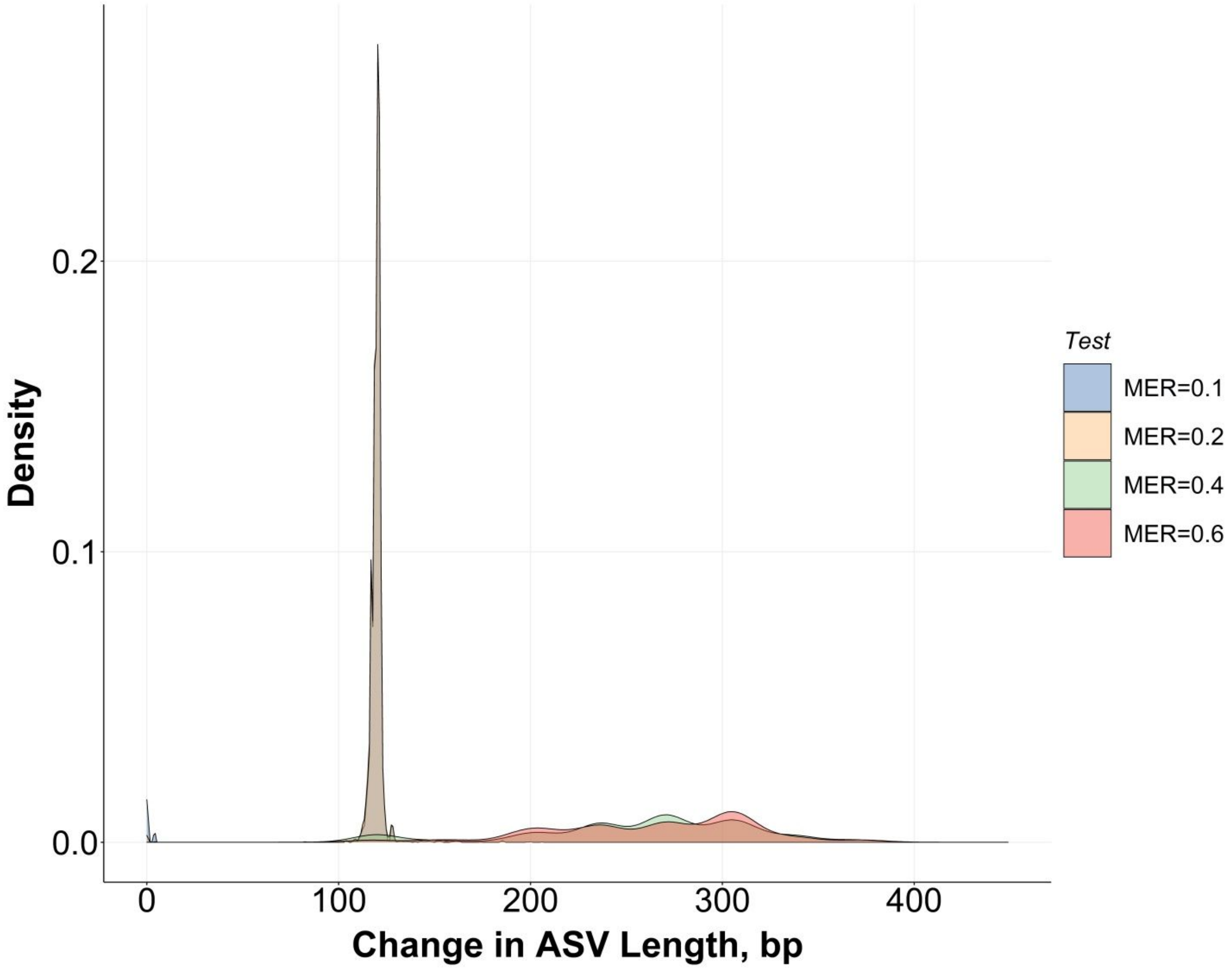

SI Figure 2. Density plot of the removed base pairs for each Cutadapt pipeline when trimming 16S rRNA gene amplicon ASV lengths from the 515f/926r primer set down to the 515f/806r binding site. MER stand for max error rate and is a parameter utilized by the cutadapt tool. Default MER is set to 0.1 while experimental testing targeted supplementary MERs of 0.2, 0.4, and 0.6, as outlined in the methods.

SI Table 7: ANOVA results for the comparison of ASV length distributions post trimming.

ANOVA Comparison	P-value
**Control-All Samples Post Trim**	0.000
**MER=0.1-All Samples Post Trim**	0.000
**MER=0.2-All Samples Post Trim**	0.988
**MER=0.4-All Samples Post Trim**	0.000
**MER=0.6-All Samples Post Trim**	0.000

**MER=0.1-Control**	0.000
**MER=0.2-Control**	0.000
**MER=0.4-Control**	0.000
**MER=0.6-Control**	0.000
**MER=0.2-MER=0.1**	0.000
**MER=0.4-MER=0.1**	0.000
**MER=0.6-MER=0.1**	0.000
**MER=0.4-MER=0.2**	0.000
**MER=0.6-MER=0.2**	0.000
**MER=0.6-MER=0.4**	0.000

SI Table 8: ANOVA results for the change in ASV length post trimming.

ANOVA Comparison	P-value
**MER=0.2-MER=0.1**	0.000
**MER=0.4-MER=0.1**	0.000
**MER=0.6-MER=0.1**	0.000
**MER=0.4-MER=0.2**	0.000
**MER=0.6-MER=0.2**	0.000
**MER=0.6-MER=0.4**	0.000

SI Table 9: Post trimming results based on ASV length.

ASV Length, bp	Untrimmed Control, n=9,315	MER 0.1, n=9,315	MER 0.1 + MER 0.2, n=9,315	MER 0.1 + MER 0.4, n=9,315	MER 0.1 + MER 0.6, n=9,315	All Samples Post Trim, n=71,119
<225	0.00%	0.01%	0.19%	90.89%	96.55%	0.12%
226-275	0.00%	97.16%	99.32%	9.02%	3.39%	99.53%
276-349	0.44%	0.49%	0.20%	0.06%	0.03%	0.26%
>350	99.56%	2.34%	0.28%	0.03%	0.02%	0.09%
**Total**	**100.00%**	**100.00%**	**100.00%**	**100.00%**	**100.00%**	**100.00%**

Note: MER stands for Max Error Rate and is a parameter within the cutadapt tool

SI Table 10: Post trimming results based on the change in ASV lengths.

Amount Trimmed, bp	MER 0.1, n=9,315	MER 0.1 + MER 0.2, n=9,315	MER 0.1 + MER 0.4, n=9,315	MER 0.1 + MER 0.6, n=9,315
0, Untrimmed	2.32%	0.38%	0.00%	0.00%
100-140, Ideal	96.67%	98.85%	7.86%	2.33%

80-100 & 140-160, Transitional	0.31%	0.47%	1.81%	1.92%
1-79 & 161+, Poor	0.70%	0.30%	90.33%	95.75%
**Total**	**100.00%**	**100.00%**	**100.00%**	**100.00%**

Note: MER stands for Max Error Rate and is a parameter within the cutadapt tool

SI Table 11: Skree plot output (stress) for tested ordination at various dimensions (k).

**Dimension (k)**	**Stress**					
	**All Samples, POC and POU**	**All Samples, POC**	**All Samples, POU**	**Nonpotable Reuse, POC and POU**	**Conventional Potable, POC and POU**	**Potable Reuse, POC and POU**
**1**	0.324	0.305	0.341	0.342	0.383	0.301
**2**	0.217	0.193	0.223	0.195	0.208	0.199
**3**	0.157	0.136	0.159	0.135	0.151	0.149
**4**	0.127	0.109	0.128	0.102	0.122	0.116
**5**	0.108	0.094	0.108	0.079	0.104	0.095
**6**	0.095	0.082	0.093	0.064	0.092	0.081

SI Table 12: ANOSIM and Betadisper results for comparisons made from all water uses at both the POC and POU combined.

**All Water Types, POC and POU**		**Anosim**		**Betadisper**		**Anosim**
**Comparison**	**Metadata**	**p-value**	**p-value adj**	**p-value**	**p-value adj**	**r-stat**
**Potability**	**Class1_Pot_NonPot**	**0.001**	**0.003**	**0.029**	**0.091**	0.34
**Water Use, low res**	**Classification_1**	**0.001**	**0.003**	**0.084**	**0.238**	0.38
**Water Use, high res**	**Classification_2**	**0.001**	**0.003**	0.000	0.000	0.52
**Climate**	**Climate**	**0.001**	**0.003**	0.000	0.000	0.13
**Region**	**Region**	**0.001**	**0.003**	0.000	0.000	0.19
**POC vs POU**	**Sample_Local**	**0.001**	**0.003**	0.000	0.000	0.23
**Residual Disinfectant**	**Final_Disinfection_Residual**	**0.001**	**0.003**	0.000	0.000	0.37
**Primer Type**	**Primers**	**0.001**	**0.003**	0.000	0.000	0.46
**Extraction Type**	**Extraction_Type**	**0.001**	**0.003**	0.000	0.000	0.37

SI Table 13: ANOSIM and Betadisper results for comparisons made from all water uses at the POC.

All Water Types, POC		Anosim		Betadisper		Anosim
**Comparison**	**Metadata**	**p-value**	**p-value adj**	**p-value**	**p-value adj**	**r-stat**
**Potability**	**Class1_Pot_NonPot**	**0.001**	**0.003**	0.000	0.001	0.61
**Water Use, low res**	**Classification_1**	**0.001**	**0.003**	**0.038**	**0.104**	0.71
**Water Use, high res**	**Classification_2**	**0.001**	**0.003**	0.000	0.000	0.81
**Climate**	**Climate**	**0.001**	**0.003**	0.000	0.000	0.13
**Region**	**Region**	**0.001**	**0.003**	0.000	0.000	0.33
**Residual Disinfectant**	**Final_Disinfection_Residual**	**0.001**	**0.003**	0.000	0.000	0.60

SI Table 14: ANOSIM and Betadisper results for comparisons made from all water uses at the POU.

All Water Types, POU		Anosim		Betadisper		Anosim
**Comparison**	**Metadata**	**p-value**	**p-value adj**	**p-value**	**p-value adj**	**r-stat**
**Potability**	**Class1_Pot_NonPot**	**0.001**	**0.002**	**0.099**	**0.364**	0.33
**Water Use, low res**	**Classification_1**	**0.001**	**0.002**	**0.672**	**1.000**	0.41
**Water Use, high res**	**Classification_2**	**0.001**	**0.002**	**0.672**	**1.000**	0.41
**Climate**	**Climate**	**0.001**	**0.002**	0.000	0.000	0.14
**Region**	**Region**	**0.001**	**0.002**	0.000	0.000	0.15
**Residual Disinfectant**	**Final_Disinfection_Residual**	**0.001**	**0.002**	0.000	0.000	0.10

SI Table 15: ANOSIM and Betadisper results for comparisons made using conventional potable waters at the POC and POU combined.

Conventional Potable, POC and POU		Anosim		Betadisper		Anosim
**Comparison**	**Metadata**	**p-value**	**p-value adj**	**p-value**	**p-value adj**	**r-stat**
**Climate**	**Climate**	**0.001**	**0.003**	0.000	0.000	0.30
**Region**	**Region**	**0.001**	**0.003**	0.000	0.000	0.48

POC vs POU	Sample_Local	**0.001**	**0.003**	0.003	**0.016**	0.26
Residual Disinfectant	Final_Disinfection_Residual	**0.001**	**0.003**	**0.256**	**0.922**	0.48
Source Water Origin	Potable_Source_Water	**0.001**	**0.003**	0.007	**0.034**	0.58
Primary Disinfectant	Potable_Disinfection_Primary	**0.001**	**0.003**	0.000	0.000	0.44
Treatment Train	Potable_Treatment_Type	**0.001**	**0.003**	0.000	0.000	0.54
Disinfection Processess	Disinfection_Total	**0.001**	**0.003**	0.000	0.000	0.46
Total Treatments	Treatment_Total	**0.001**	**0.003**	0.000	0.000	0.54
Numerical Treatment Classification	Treatment_Classification_Number	**0.001**	**0.003**	**0.014**	**0.065**	0.48
Categorical Treatment Classification	Treatment_Classification_Cat	**0.001**	**0.003**	**0.105**	**0.404**	0.61
Mixed Source Water	Mixed_Source_Water	**0.001**	**0.003**	0.007	**0.034**	0.59
Overlapped with Nonpotable	Potable_Overlap	**0.001**	**0.003**	**0.286**	**0.966**	0.61
Uses Ozone	Includes_Ozone	**0.001**	**0.003**	**0.066**	**0.276**	0.64
Uses RO or UF	Includes_RO_UF	**0.001**	**0.003**	0.000	0.000	0.33
Uses UV	Includes_UV	**0.001**	**0.003**	0.000	0.000	0.28

SI Table 16: ANOSIM and Betadisper results for comparisons made using potable reuse waters at the POC and POU combined.

Potable Reuse, POC and POU		Anosim		Betadisper		Anosim
Comparison	Metadata	p-value	p-value adj	p-value	p-value adj	r-stat
IPR vs DPR	Classification_2v2	**0.001**	**0.006**	0.000	0.000	0.31
Climate	Climate	**0.001**	**0.006**	**0.074**	**0.420**	0.42
Region	Region	**0.001**	**0.006**	0.000	0.000	0.31
POC vs POU	Sample_Local	**0.001**	**0.006**	**0.530**	**1.000**	0.29
Residual Disinfectant	Final_Disinfection_Residual	**0.001**	**0.006**	0.002	**0.017**	0.35
Source Water Origin	Potable_Source_Water	**0.001**	**0.006**	0.000	0.006	0.23

**Primary Disinfectant**	**Potable_Disinfection_Primary**	0.742	1.000	0.000	0.000	-0.05
**Treatment Train**	**Potable_Treatment_Type**	0.716	1.000	0.000	0.000	-0.05
**Disinfection Processess**	**Disinfection_Total**	**0.001**	**0.006**	**0.799**	**1.000**	0.60
**Total Treatments**	**Treatment_Total**	**0.001**	**0.006**	**0.799**	**1.000**	0.60
**Numerical Treatment Classification**	**Treatment_Classification_Number**	0.040	0.192	**0.010**	**0.090**	0.13
**Categorical Treatment Classification**	**Treatment_Classification_Cat**	0.566	1.000	**0.076**	**0.420**	-0.02
**Overlapped with Nonpotable**	**Potable_Overlap**	0.573	1.000	**0.076**	**0.420**	-0.02
**Uses Ozone**	**Includes_Ozone**	**0.005**	0.026	**0.978**	**1.000**	0.12
**Uses RO or UF**	**Includes_RO_UF**	0.082	0.368	**0.118**	**0.605**	0.06
**Uses UV**	**Includes_UV**	**0.001**	**0.006**	**0.279**	**1.000**	0.21
**Additional Treatment**	**Reuse.Reclaimed_Treatment**	**0.001**	**0.006**	**0.061**	**0.420**	0.39
**Blending**	**Reuse.Reclaimed_Blending**	**0.001**	**0.006**	0.001	0.009	0.26
**Post Blend Disinfection**	**PostBlend_Disinfection**	**0.001**	**0.006**	**0.021**	**0.171**	0.37
**Post Blend Treatment**	**PostBlend_Treatment**	**0.001**	**0.006**	**0.225**	**1.000**	0.38

SI Table 17: ANOSIM and Betadisper results for comparisons made using non-potable reuse waters at the POC and POU combined.

**Non-potable Reuse, POC and POU**		**Anosim**		**Betadisper**		**Anosim**
**Comparison**	**Metadata**	**p-value**	**p-value adj**	**p-value**	**p-value adj**	**r-stat**
**Climate**	**Climate**	0.001	0.004	0.066	0.216	0.19
**Region**	**Region**	0.001	0.004	0.000	0.000	0.20
**POC vs POU**	**Sample_Local**	0.001	0.004	0.000	0.000	0.55
**Residual Disinfectant**	**Final_Disinfection_Residual**	0.001	0.004	0.000	0.000	0.67
**Disinfection Processess**	**Disinfection_Total**	0.001	0.004	0.000	0.000	0.60
**Total Treatments**	**Treatment_Total**	0.001	0.004	0.000	0.000	0.84

**Numerical Treatment Classification**	**Treatment_Classification_Number**	0.001	0.004	0.045	0.164	0.38
**Categorical Treatment Classification**	**Treatment_Classification_Cat**	0.002	0.007	0.181	0.531	0.09
**Uses UV**	**Includes_UV**	0.004	0.012	0.018	0.077	0.10
**Treatment Train**	**Reuse.Reclaimed_Treatment**	0.001	0.004	0.000	0.000	0.84

SI Table 18. Adonis2 output for multiple comparisons within conventional potable waters.

```
Permutation test for adonis under reduced model
Terms added sequentially (first to last)
Permutation: free
Number of permutations: 1000

adonis2(formula = All.PotablePOCPOU.dist.bray ~ Treatment_Classification_Cat + Mixed_Source_Water + Potable_Source_Water + Treatment_Classification_Number + Potabl
e_Overlap + Final_Disinfection_Residual + Sample_Local, data = All.PotablePOCPOU.meta, permutations = permutations, by = "terms")
                                 Df SumOfSqs      R2       F   Pr(>F)
Treatment_Classification_Cat      2   12.844 0.13566 20.9968 0.000999 ***
Mixed_Source_Water                1    3.121 0.03296 10.2033 0.000999 ***
Potable_Source_Water              1    0.905 0.00956  2.9592 0.001998 **
Treatment_Classification_Number   3    8.465 0.08941  9.2250 0.000999 ***
Final_Disinfection_Residual       1    1.502 0.01587  4.9121 0.000999 ***
Sample_Local                      1    1.162 0.01227  3.7982 0.000999 ***
Residual                        218   66.679 0.70427
Total                           227   94.678 1.00000
---
Signif. codes:  0 ‘***’ 0.001 ‘**’ 0.01 ‘*’ 0.05 ‘.’ 0.1 ‘ ’ 1
```

SI Table 19. Adonis2 output for multiple comparisons within potable reuse waters.

```
Permutation test for adonis under reduced model
Terms added sequentially (first to last)
Permutation: free
Number of permutations: 1000

adonis2(formula = All.PotableReusePOCPOU.dist.bray ~ Treatment_Total + Disinfection_Total + Final_Disinfection_Residual + Sample_Local + Climate, data = All.Potabl
eReusePOCPOU.meta, permutations = permutations, by = "terms")
                Df SumOfSqs      R2      F   Pr(>F)
Treatment_Total 10   8.5550 0.36969 2.5860 0.000999 ***
Sample_Local     1   1.0221 0.04417 3.0895 0.000999 ***
Residual        41  13.5636 0.58614
Total           52  23.1407 1.00000
---
Signif. codes:  0 ‘***’ 0.001 ‘**’ 0.01 ‘*’ 0.05 ‘.’ 0.1 ‘ ’ 1
```

SI Table 20. Adonis2 output for multiple comparisons within non-potable reuse waters. Option1 with only homogenously dispersed comparisons.

```
Permutation test for adonis under reduced model
Terms added sequentially (first to last)
Permutation: free
Number of permutations: 1000

adonis2(formula = All.NonPotableReusePOCPOU.dist.bray ~ Treatment_Classification_Number + Treatment_Classification_Cat + Climate, data = All.NonPotableReusePOCPOU.
meta, permutations = permutations, by = "terms")
                                 Df SumOfSqs      R2      F   Pr(>F)
Treatment_Classification_Number   3   12.868 0.17821 13.263 0.000999 ***
Climate                           1    4.358 0.06035 13.474 0.000999 ***
Residual                        170   54.982 0.76144
Total                           174   72.208 1.00000
---
Signif. codes:  0 ‘***’ 0.001 ‘**’ 0.01 ‘*’ 0.05 ‘.’ 0.1 ‘ ’ 1
```

SI Table 21. Adonis2 output for multiple comparisons within non-potable reuse waters. Option2 with homogenously and heterogeneously dispersed comparisons.

```
Permutation test for adonis under reduced model
Terms added sequentially (first to last)
Permutation: free
Number of permutations: 1000

adonis2(formula = All.NonPotableReusePOCPOU.dist.bray ~ Treatment_Classification_Number + Treatment_Classification_Cat + Climate + Final_Disinfection_Residual + Tr
eatment_Total, data = All.NonPotableReusePOCPOU.meta, permutations = permutations, by = "terms")
                               Df SumOfSqs      R2       F   Pr(>F)    
Treatment_Classification_Number   3   12.868 0.17821 15.8118 0.000999 ***
Climate                         1    4.358 0.06035 16.0637 0.000999 ***
Final_Disinfection_Residual     1    6.797 0.09414 25.0569 0.000999 ***
Treatment_Total                 2    2.881 0.03989  5.3093 0.000999 ***
Residual                      167   45.304 0.62741                   
Total                         174   72.208 1.00000                   
---
Signif. codes:  0 '***' 0.001 '**' 0.01 '*' 0.05 '.' 0.1 ' ' 1
```

SI Table 22. Phylum based core and discriminatory analysis of taxa identified by average frequency of detection (decimal %)[1] and supplemented with differentially abundant taxa identified by ANCOM testing. Note, the frequency of detection utilized a modified dataset.

Phylum	Potable Conventional, Frequency of Detection			Potable Reuse, Frequency of Detection			Non-potable Reuse, Frequency of Detection			Differential Abundance, padj<0.01, LFC		
	POC and POU	POC	POU	POC and POU	POC	POU	POC and POU	POC	POU	POC and POU	POC	POU
Proteobacteria	***1.00***	***1.00***	***1.00***	***0.98***	***1.00***	***0.98***	***1.00***	***1.00***	***1.00***			
Actinobacteriota	***0.95***	***0.93***	***0.95***	***0.85***	***0.93***	***0.85***	***0.94***	***0.91***	***0.94***	-1.63	-2.54	-1.08
Bacteroidota	***0.84***	***0.84***	***0.84***	***0.83***	***0.93***	***0.83***	***1.00***	***1.00***	***1.00***	***2.23***	***2.40***	***2.30***
Firmicutes	0.70	***0.81***	0.70	***0.83***	***0.93***	***0.83***	***0.97***	***1.00***	***0.97***	***1.52***	***2.39***	***1.44***
Planctomycetota	0.75	0.71	0.75	0.79	***0.96***	0.79	***1.00***	***1.00***	***1.00***	***0.93***		***1.25***
Cyanobacteria	***0.85***	0.76	***0.85***	***0.81***	***0.85***	***0.81***	***0.89***	***0.80***	***0.89***	-1.72	-3.45	-1.02
Bdellovibrionota	0.41	0.36	0.41	0.75	***0.93***	0.75	***0.98***	***0.98***	***0.98***	***2.74***	***3.08***	***3.07***
Acidobacteriota	0.59	0.57	0.59	0.51	0.48	0.51	***0.90***	***0.88***	***0.90***			
Verrucomicrobiota	0.51	0.44	0.51	0.60	0.67	0.60	***0.99***	***1.00***	***0.99***	***2.25***	***2.03***	***2.37***
Myxococcota	0.33	0.28	0.33	0.53	0.59	0.53	***0.96***	***1.00***	***0.96***	***2.24***	***3.76***	***1.43***
Patescibacteria	0.20	0.21	0.20	0.42	0.41	0.42	***0.94***	***0.99***	***0.94***	***3.49***	***5.29***	***2.43***
Chloroflexi	0.17	0.13	0.17	0.47	0.52	0.47	0.75	***0.96***	0.75	***1.31***	***3.53***	
Dependentiae	0.26	0.22	0.26	0.43	0.37	0.43	***0.87***	0.79	***0.87***	***1.91***	***1.44***	***2.49***
Desulfobacterota	0.18	0.09	0.18	0.25	0.15	0.25	***0.91***	***0.90***	***0.91***	***1.86***	***2.80***	***1.54***
Campilobacterota	0.19	0.07	0.19	0.23	0.19	0.23	0.73	0.78	0.73	***1.56***	***2.49***	
Spirochaetota	0.11	0.10	0.11	0.15	0.15	0.15	0.73	***0.90***	0.73	***1.10***	***2.67***	

[1] Core taxa are bolded with discriminatory taxa are highlighted in red.

SI Table 23. Genus based core and discriminatory analysis of taxa identified by average frequency of detection (decimal %)[1] and supplemented with differentially abundant taxa identified by ANCOM testing. Note, the frequency of detection utilized a modified dataset.

Genus	Potable Conventional, Frequency of Detection			Potable Reuse, Frequency of Detection			Non-potable Reuse, Frequency of Detection			Differential Abundance, padj<0.01, LFC		
	POC and POU	POC	POU	POC and POU	POC	POU	POC and POU	POC	POU	POC and POU	POC	POU
Sphingomonas	***0.84***	***0.98***	***0.84***	***0.96***	***1.00***	***0.96***	0.61	0.25	0.61	-2.98	-6.33	-1.43
Pseudomonas	0.67	0.72	0.67	0.79	***0.89***	0.79	***0.86***	0.73	***0.86***	***1.06***		***2.51***
Ralstonia	***0.89***	***0.93***	***0.89***	0.74	***0.85***	0.74	0.23	0.20	0.23	-4.07	-4.60	-3.60
Mycobacterium	0.80	***0.88***	0.80	0.77	0.78	0.77	0.65	0.57	0.65	-2.53	-3.77	-2.46
Novosphingobium	0.64	0.63	0.64	0.70	***0.81***	0.70	***0.92***	***0.86***	***0.92***	***1.52***		***2.32***
Hyphomicrobium	0.74	***0.91***	0.74	0.36	0.37	0.36	***0.89***	***0.83***	***0.89***			***1.18***
Bradyrhizobium	***0.84***	***0.93***	***0.84***	0.57	0.56	0.57	0.40	0.35	0.40	-2.98	-4.19	-2.41
Obscuribacteraceae	0.73	***0.95***	0.73	0.53	0.59	0.53	0.50	0.30	0.50	-2.31	-4.49	-1.50
Bosea	0.64	***0.82***	0.64	0.49	0.67	0.49	0.69	0.38	0.69		-2.58	***1.75***
Methylobacterium.Methylorubrum	0.67	***0.84***	0.67	***0.87***	0.78	***0.87***	0.19	0.10	0.19			
Aquabacterium	0.40	0.47	0.40	0.58	0.48	0.58	0.73	***0.88***	0.73	***0.96***		
Sediminibacterium	0.49	0.42	0.49	0.42	0.33	0.42	***0.83***	***0.94***	***0.83***	***1.68***	***2.21***	
Legionella	0.31	0.28	0.31	0.51	0.56	0.51	***0.88***	***0.85***	***0.88***	***2.09***	***1.75***	***2.32***
Bdellovibrio	0.28	0.14	0.28	0.47	0.52	0.47	***0.93***	***0.91***	***0.93***	***2.48***	***2.18***	***2.91***
Curvibacter	0.60	***0.88***	0.60	0.43	0.37	0.43	0.34	0.32	0.34	-2.00	-2.89	-2.02
Acidovorax	0.36	0.42	0.36	0.42	0.37	0.42	***0.85***	0.78	***0.85***	***2.59***		***3.57***
Flavobacterium	0.23	0.25	0.23	0.42	0.48	0.42	***0.96***	***0.96***	***0.96***	***4.79***	***4.71***	***4.71***
Methylotenera	0.25	0.26	0.25	0.55	0.63	0.55	0.72	***0.88***	0.72	***2.42***	***3.52***	
Porphyrobacter	0.60	***0.81***	0.60	0.36	0.33	0.36	0.56	0.22	0.56		-3.37	***1.98***
X0319.6G20	0.20	0.14	0.20	0.49	0.63	0.49	***0.82***	0.75	***0.82***			
Pedobacter	0.17	0.14	0.17	0.51	0.56	0.51	***0.86***	***0.93***	***0.86***	***2.81***	***2.92***	***2.51***
Undibacterium	0.14	0.11	0.14	0.49	0.70	0.49	***0.83***	***0.89***	***0.83***	***2.98***	***2.03***	***3.44***
SM2D12	0.42	0.39	0.42	0.19	0.15	0.19	***0.87***	***0.80***	***0.87***	***1.83***	***1.27***	***2.15***
env.OPS_17	0.23	0.16	0.23	0.30	0.26	0.30	***0.87***	***0.94***	***0.87***	***2.46***	***3.55***	***1.78***
Cloacibacterium	0.25	0.25	0.25	0.38	0.22	0.38	0.77	***0.90***	0.77	***2.36***	***2.68***	***2.12***
Methyloversatilis	0.33	0.37	0.33	0.36	0.33	0.36	0.69	***0.83***	0.69	***1.74***	***2.72***	
Hydrogenophaga	0.18	0.21	0.18	0.34	0.37	0.34	***0.87***	***0.81***	***0.87***	***3.37***	***2.39***	***4.02***
Terrimonas	0.25	0.28	0.25	0.32	0.26	0.32	0.71	***0.86***	0.71	***1.33***	***2.62***	
Rhodobacter	0.36	0.26	0.36	0.13	0.04	0.13	***0.87***	***0.83***	***0.87***	***2.55***	***2.52***	***2.46***

**Romboutsia**	0.06	0.09	0.06	0.40	0.52	0.40	0.72	***0.93***	0.72	***2.78***	***3.59***	***2.00***
**NS11.12_marine_group**	0.14	0.19	0.14	0.17	0.19	0.17	***0.85***	***0.94***	***0.85***			
**Lacunisphaera**	0.17	0.28	0.17	0.09	0.07	0.09	0.74	***0.84***	0.74	***1.31***	***1.49***	***1.02***
**Clostridium_sensu_stricto_1**	0.08	0.09	0.08	0.26	0.22	0.26	0.63	***0.84***	0.63	***1.99***	***2.50***	***1.53***
**Aeromonas**	0.09	0.04	0.09	0.13	0.11	0.13	***0.83***	***0.89***	***0.83***	***3.55***	***3.59***	***3.54***
**Haliangium**	0.07	0.00	0.07	0.21	0.26	0.21	0.69	***0.85***	0.69	***1.96***	***3.18***	***0.92***
**Kapabacteriales**	0.06	0.02	0.06	0.11	0.19	0.11	0.77	***0.90***	0.77	***2.28***	***2.93***	***1.74***
**Zoogloea**	0.03	0.00	0.03	0.26	0.30	0.26	0.62	***0.86***	0.62	***3.03***	***4.65***	***1.64***
**Thiothrix**	0.05	0.00	0.05	0.15	0.15	0.15	0.64	***0.83***	0.64	***2.44***	***4.37***	
**Gracilibacteria**	0.03	0.02	0.03	0.08	0.00	0.08	0.75	***0.93***	0.75	***3.13***	***4.96***	***1.61***
**Absconditabacteriales_.SR1.**	0.02	0.02	0.02	0.11	0.11	0.11	0.64	***0.86***	0.64			
**NS9_marine_group**	0.05	0.00	0.05	0.06	0.07	0.06	0.67	***0.83***	0.67	***2.01***	***3.06***	***1.17***
**Chitinivorax**	0.02	0.02	0.02	0.06	0.04	0.06	0.71	***0.91***	0.71	***3.29***	***4.31***	***2.45***
**vadinHA49**	0.02	0.04	0.02	0.08	0.07	0.08	0.58	***0.84***	0.58	***1.71***	***3.26***	
**JGI_0000069.P22**	0.01	0.00	0.01	0.00	0.00	0.00	0.64	***0.86***	0.64			

[1] Core taxa are bolded with discriminatory taxa highlighted in red.

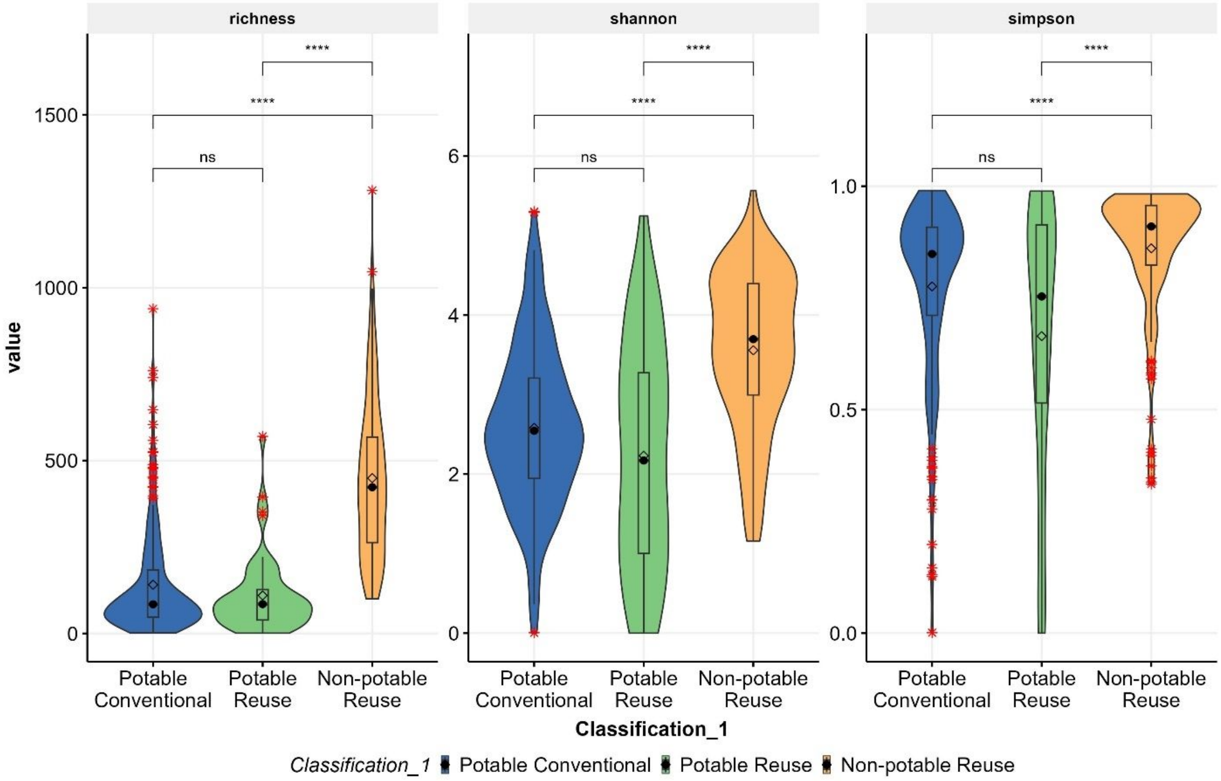

Figure 3. Violin plots of selected alpha diversity metrics for samples grouped by intended water use. Wilcoxon rank sum tests, with p-value adjustment, were used to test for significant differences between comparisons.

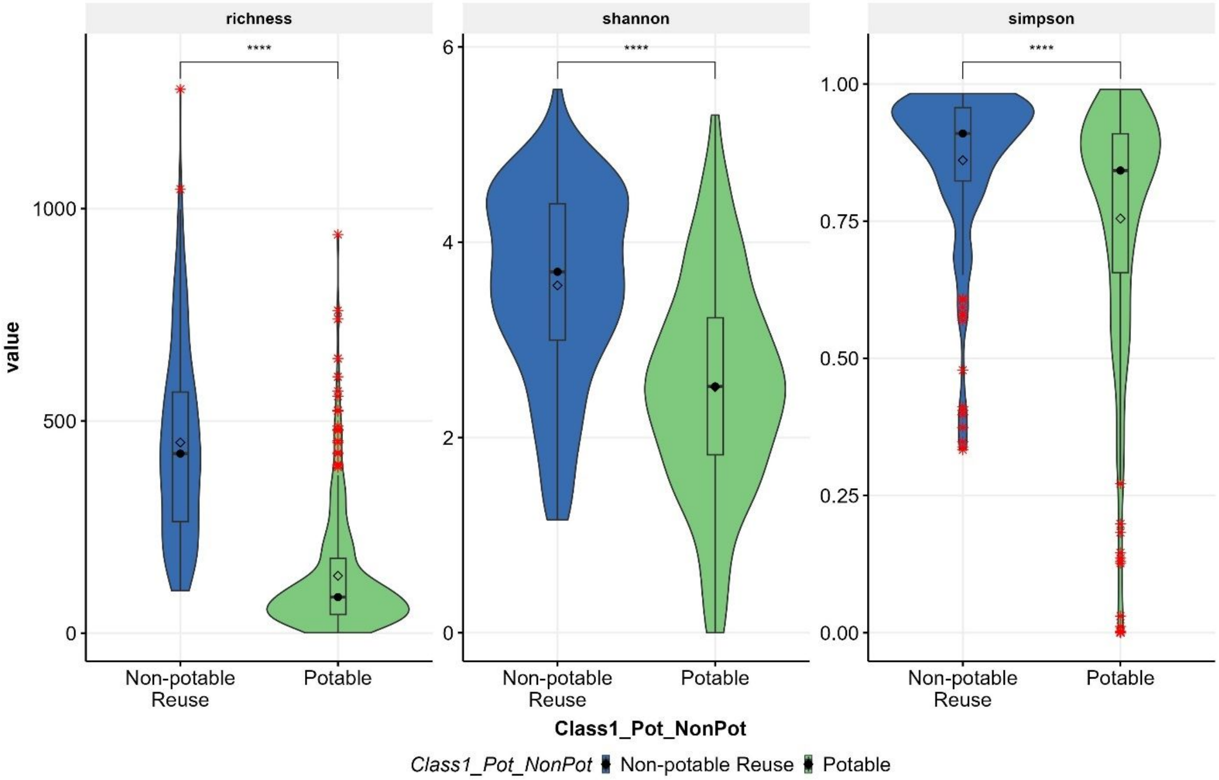

Figure 4. Violin plots of selected alpha diversity metrics for all samples grouped by potability. Wilcoxon rank sum tests, with p-value adjustment, were used to test for significant differences between comparisons.

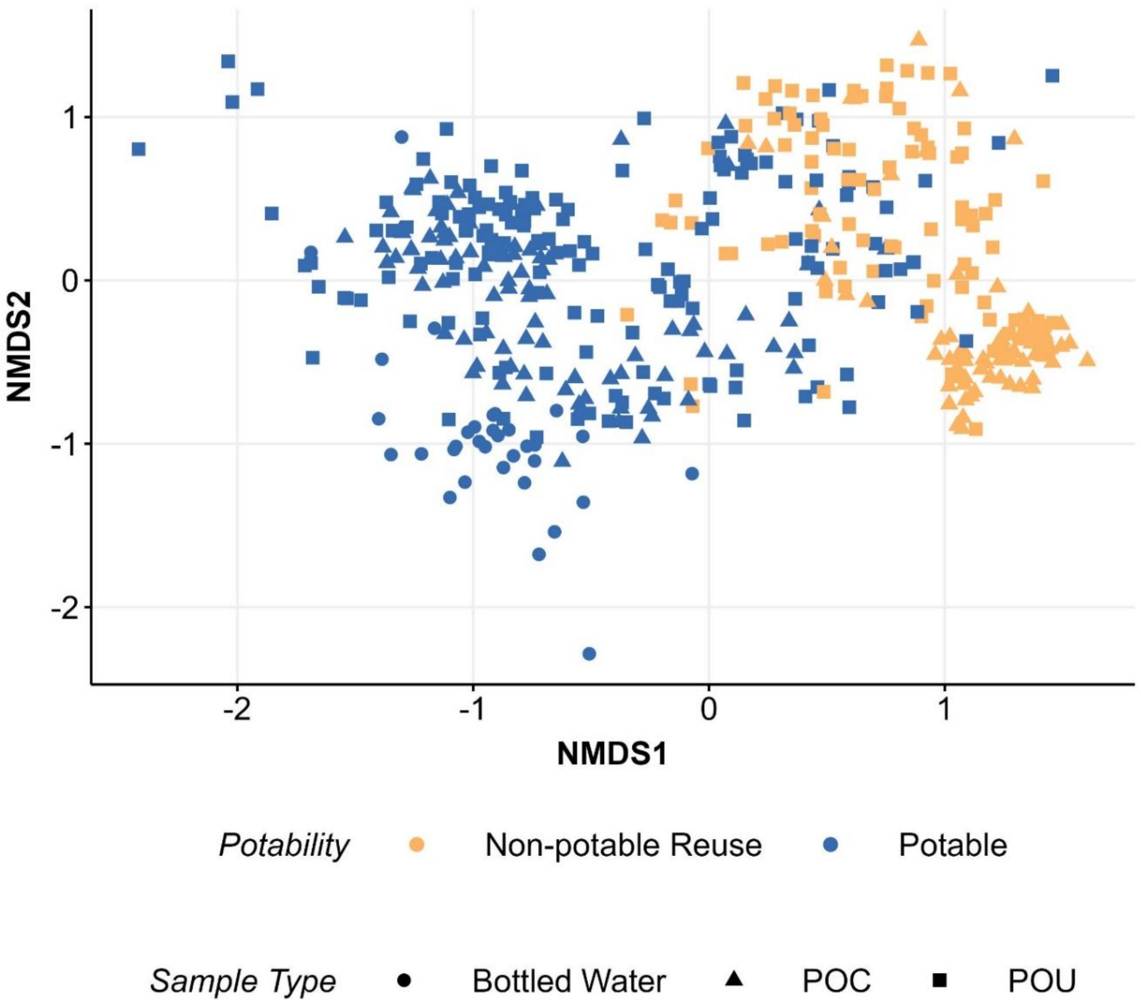

SI Figure 5. Bray-Curtis beta diversity plot for all bulk water samples regardless of distinctions between POC and POU, classified by potability.

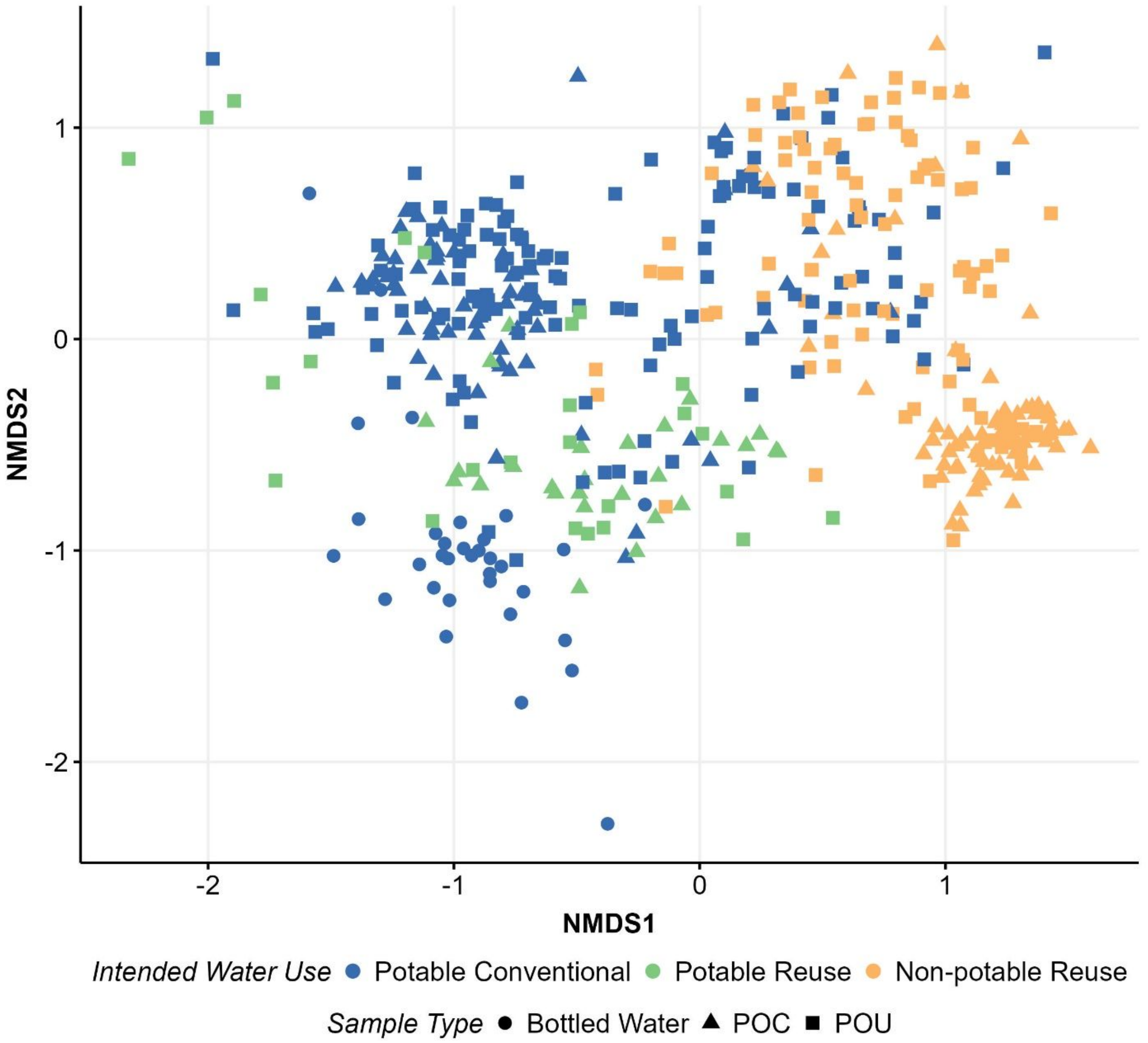

SI Figure 6. Bray-Curtis beta diversity plot for all bulk water samples regardless of distinctions between POC and POU, classified by intended water use.

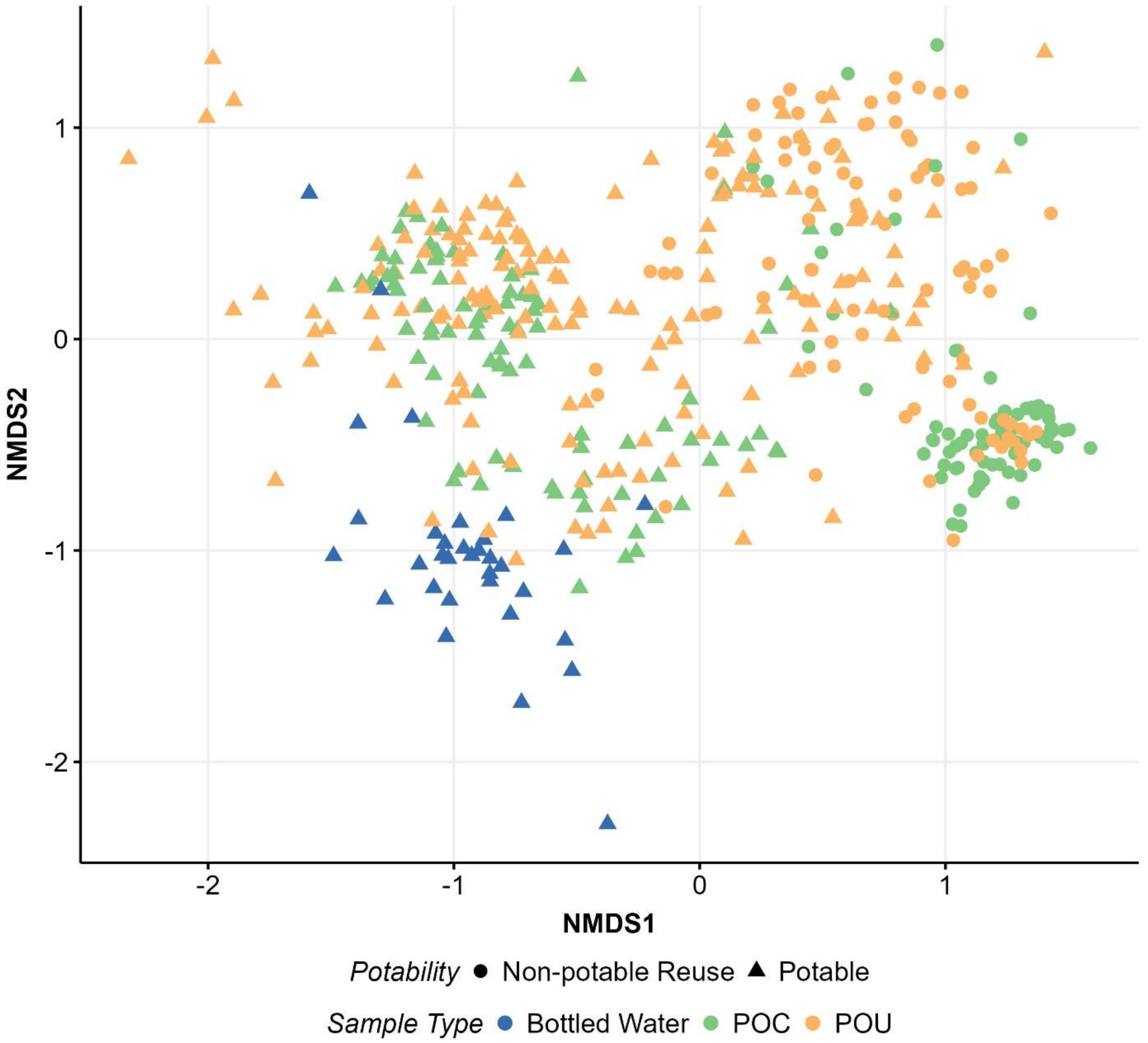

SI Figure 7. Bray-Curtis beta diversity plot for all bulk water samples of interest, classified by potability and sample location related to the POC or POU.

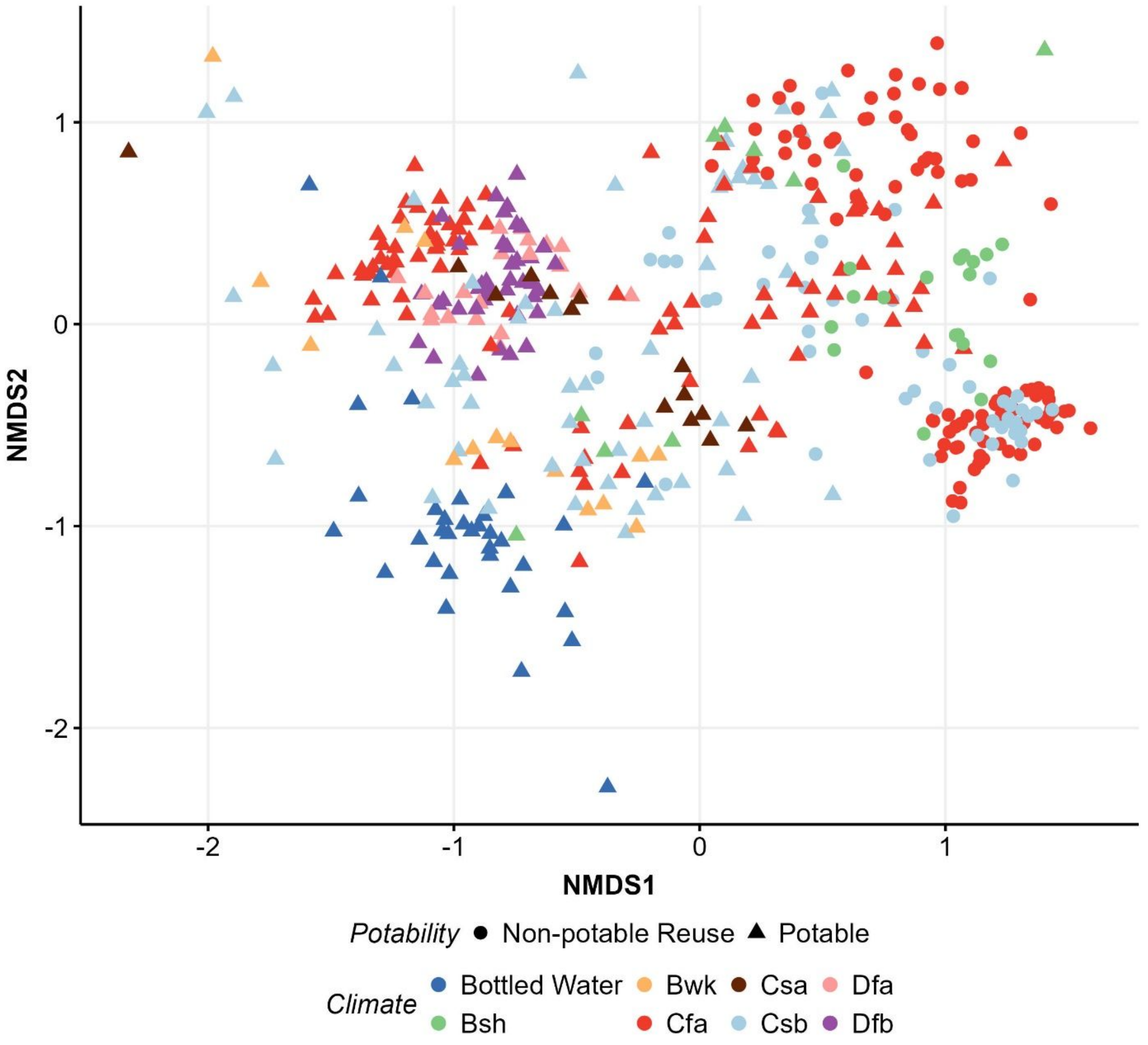

SI Figure 8. Bray-Curtis beta diversity plot for all bulk water samples of interest, classified by potability and Köppen climate designator.

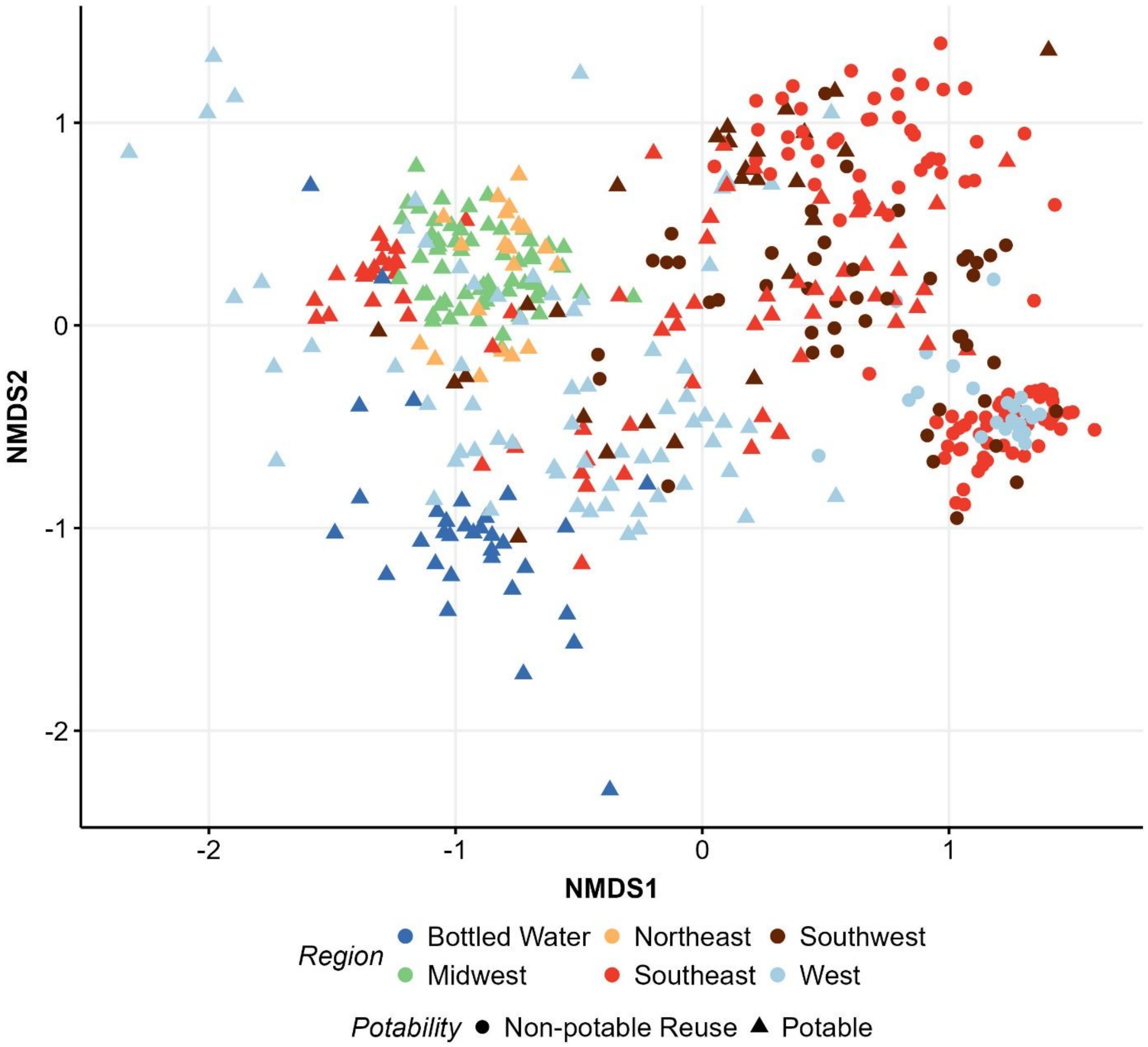

SI Figure 9. Bray-Curtis beta diversity plot for all bulk water samples of interest, classified by potability and regional designator.

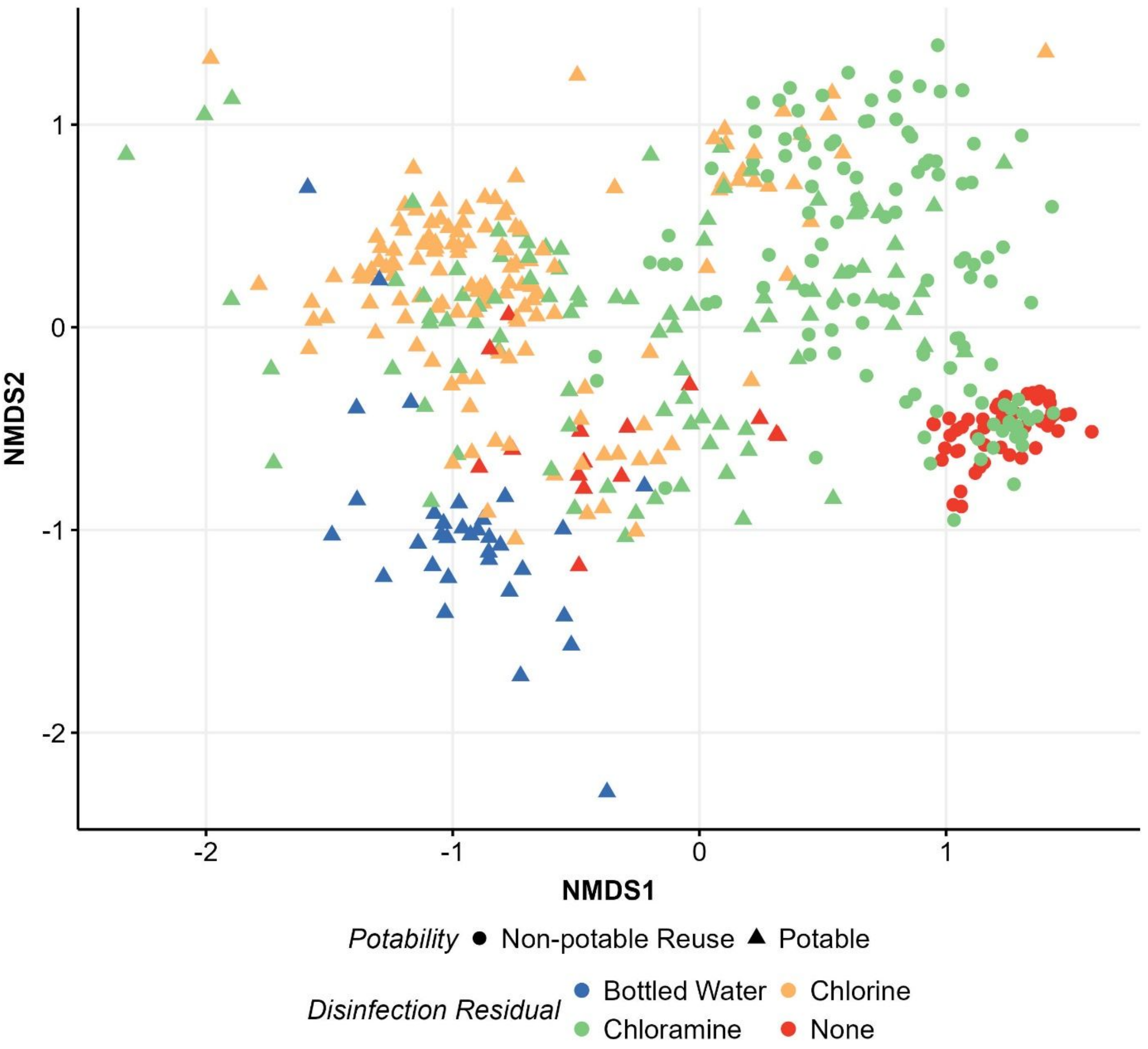

SI Figure 10. Bray-Curtis beta diversity plot for all bulk water samples of interest, classified by potability and disinfection residual.

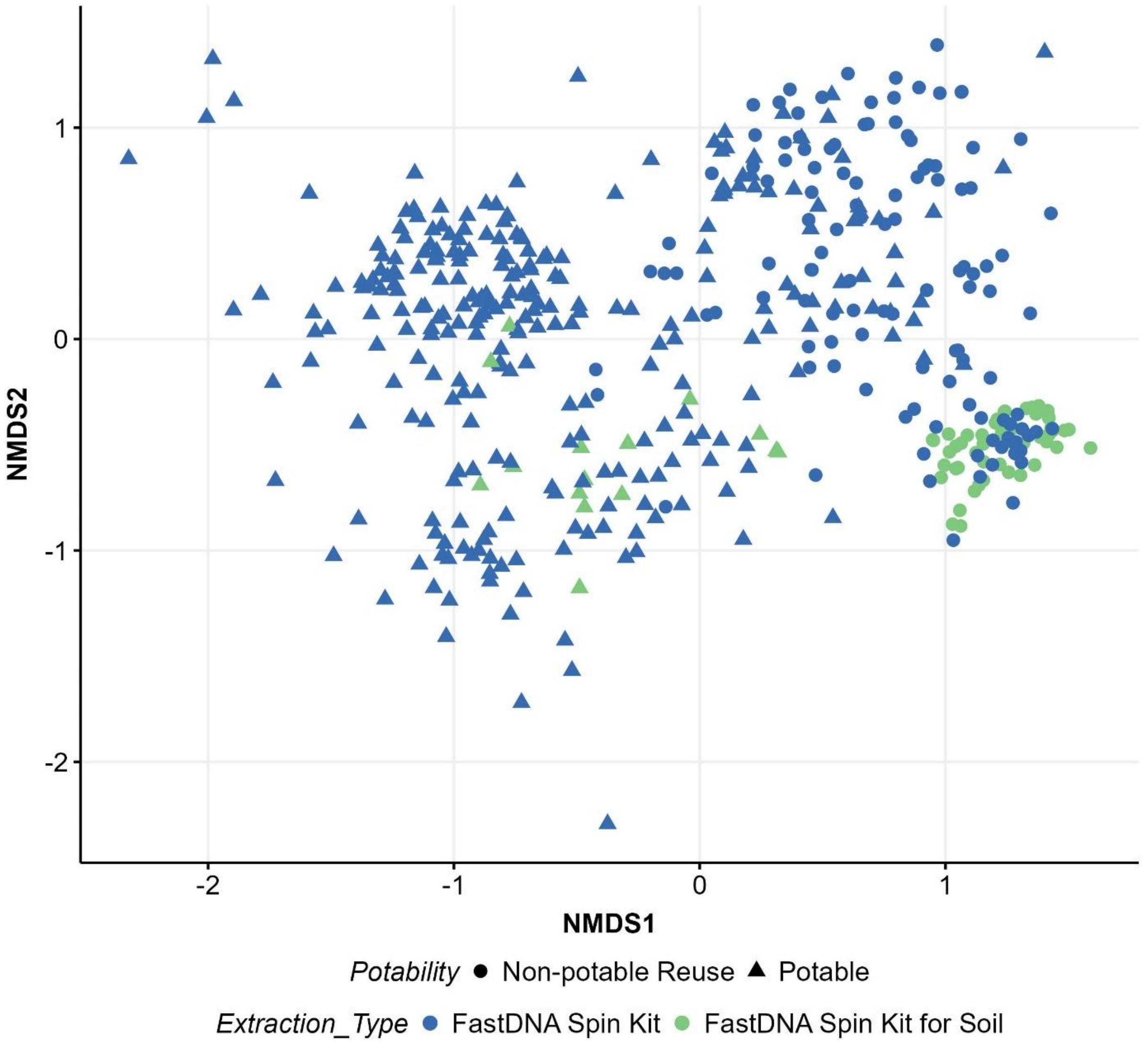

SI Figure 11. Bray-Curtis beta diversity plot for all bulk water samples of interest, classified by DNA extraction kit and potability.

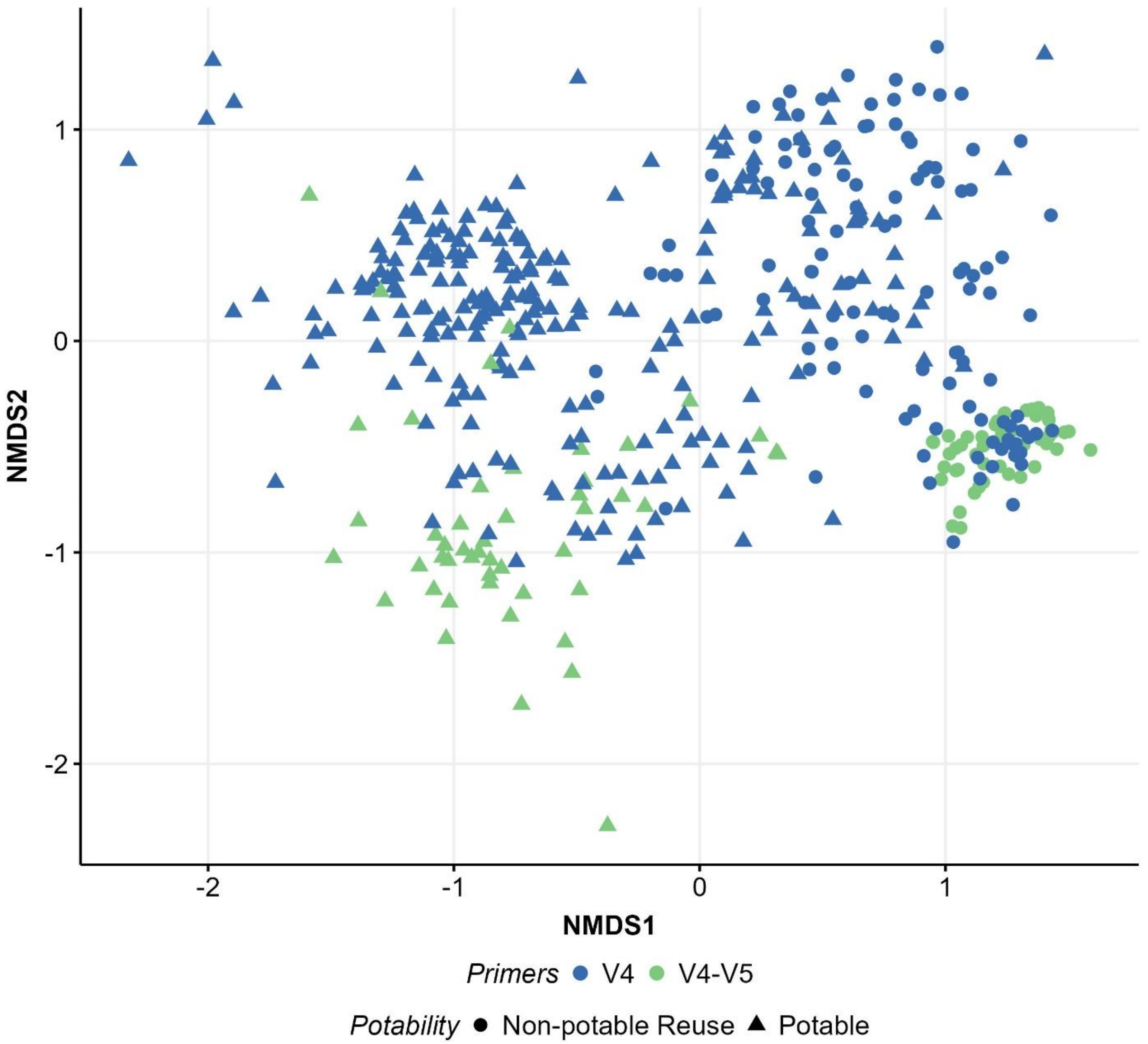

SI Figure 12. Bray-Curtis beta diversity plot for all bulk water samples of interest, classified by potability and sequencing primers.

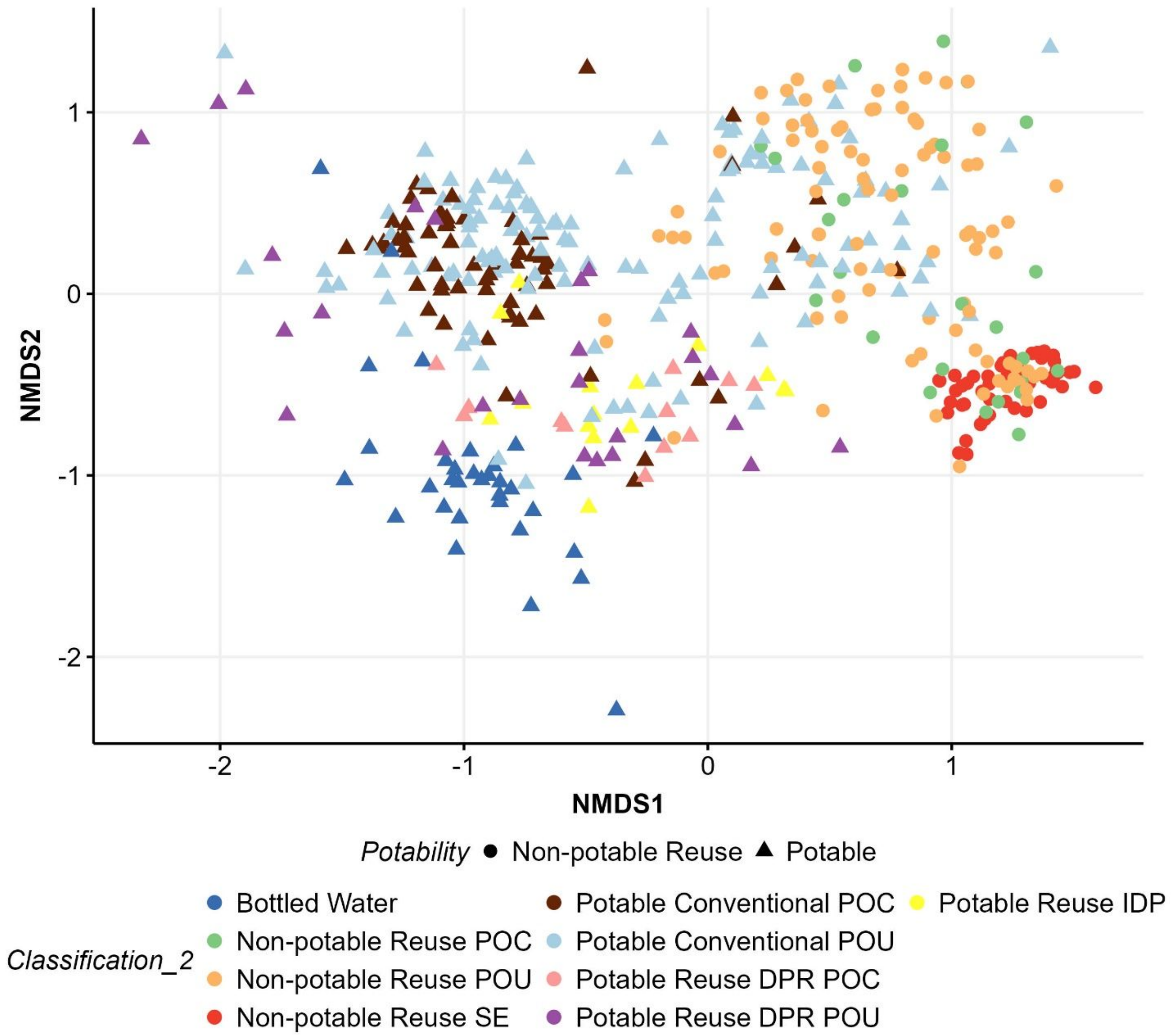

SI Figure 13. Bray-Curtis beta diversity plot for all bulk water samples of interest, classified by potability and higher resolution water use.

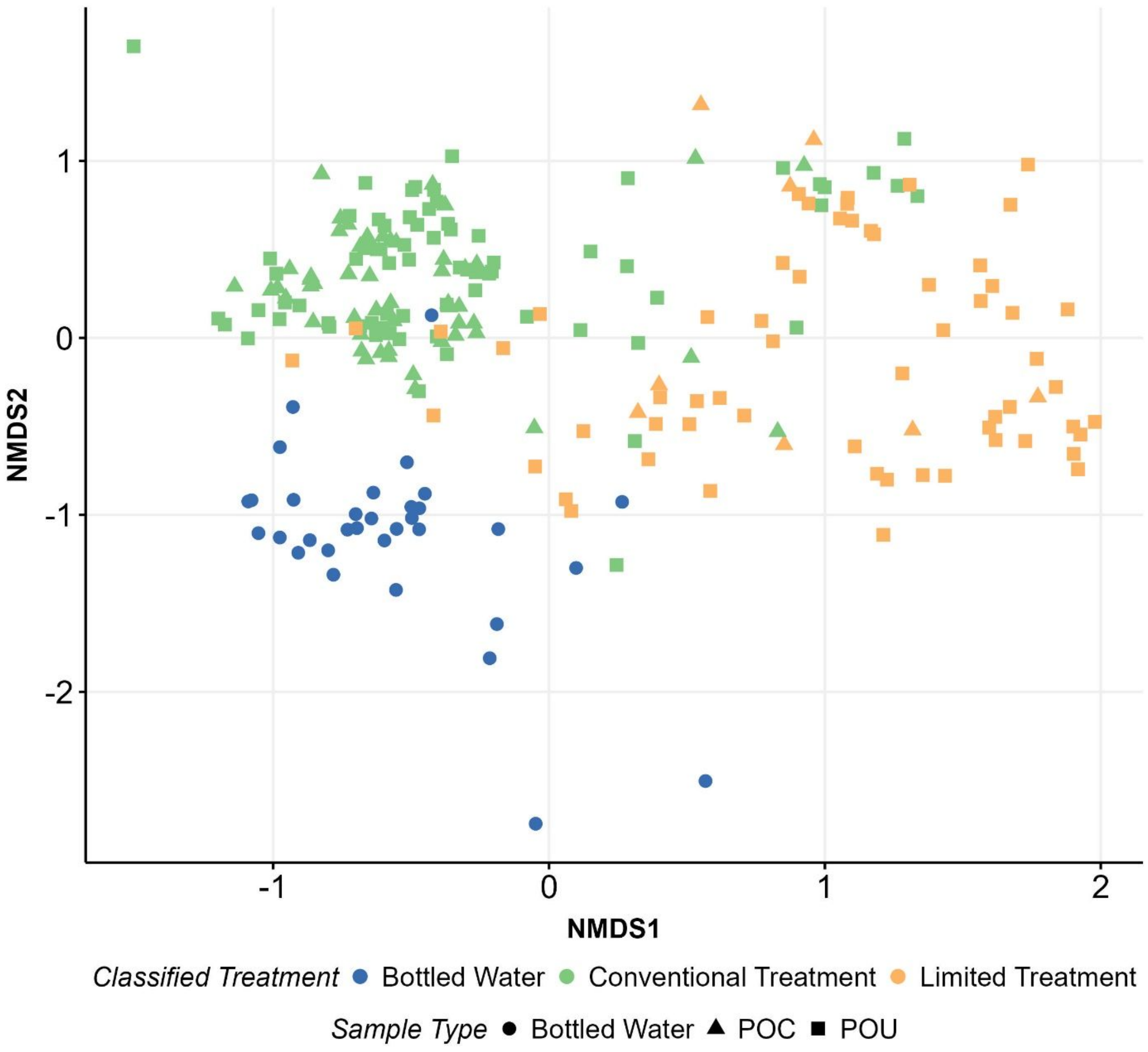

SI Figure 14. Bray-Curtis beta diversity plot for all potable bulk water samples, classified by classified treatment.

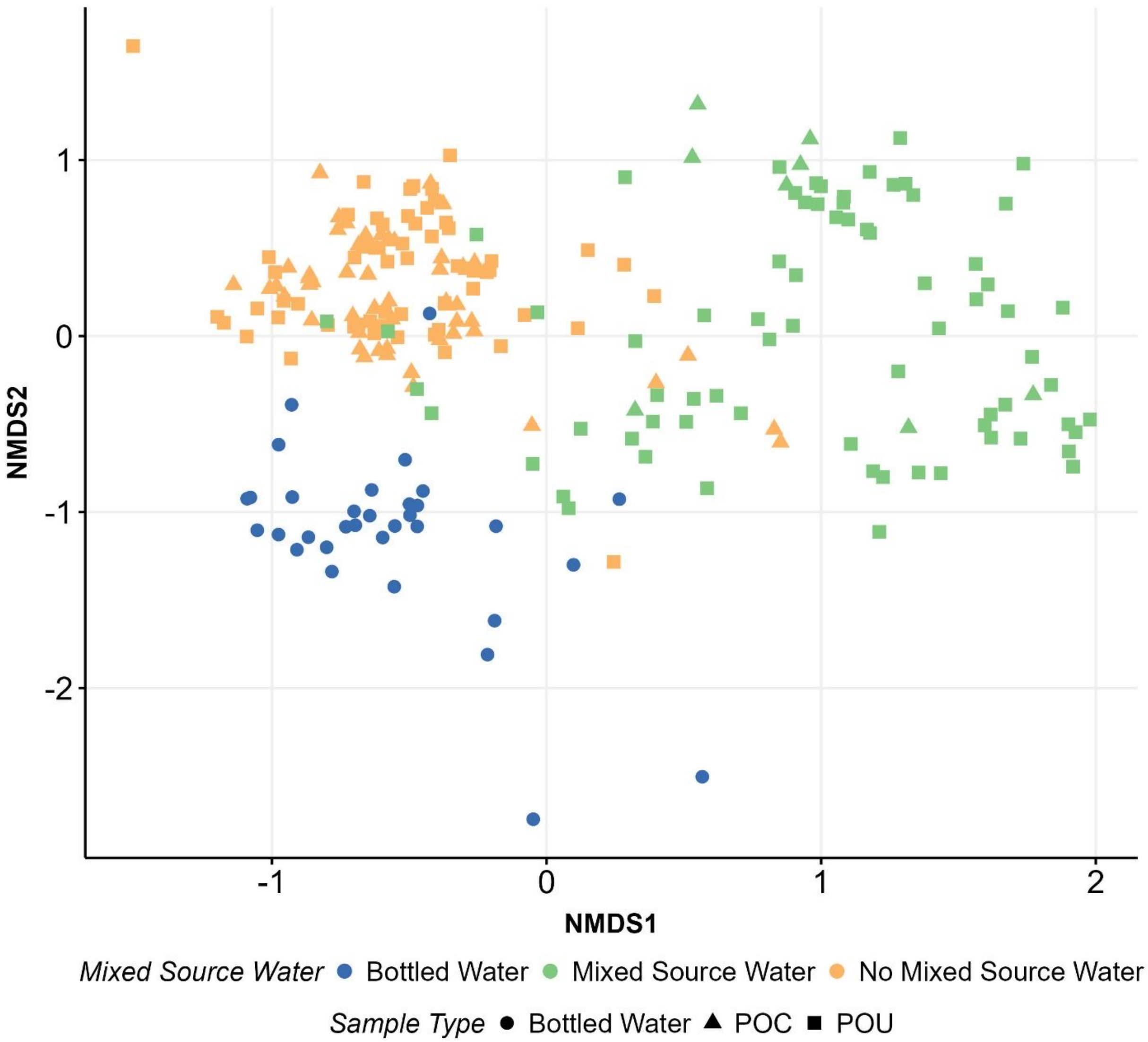

SI Figure 15. Bray-Curtis beta diversity plot for all potable bulk water samples, classified by mixed source water.

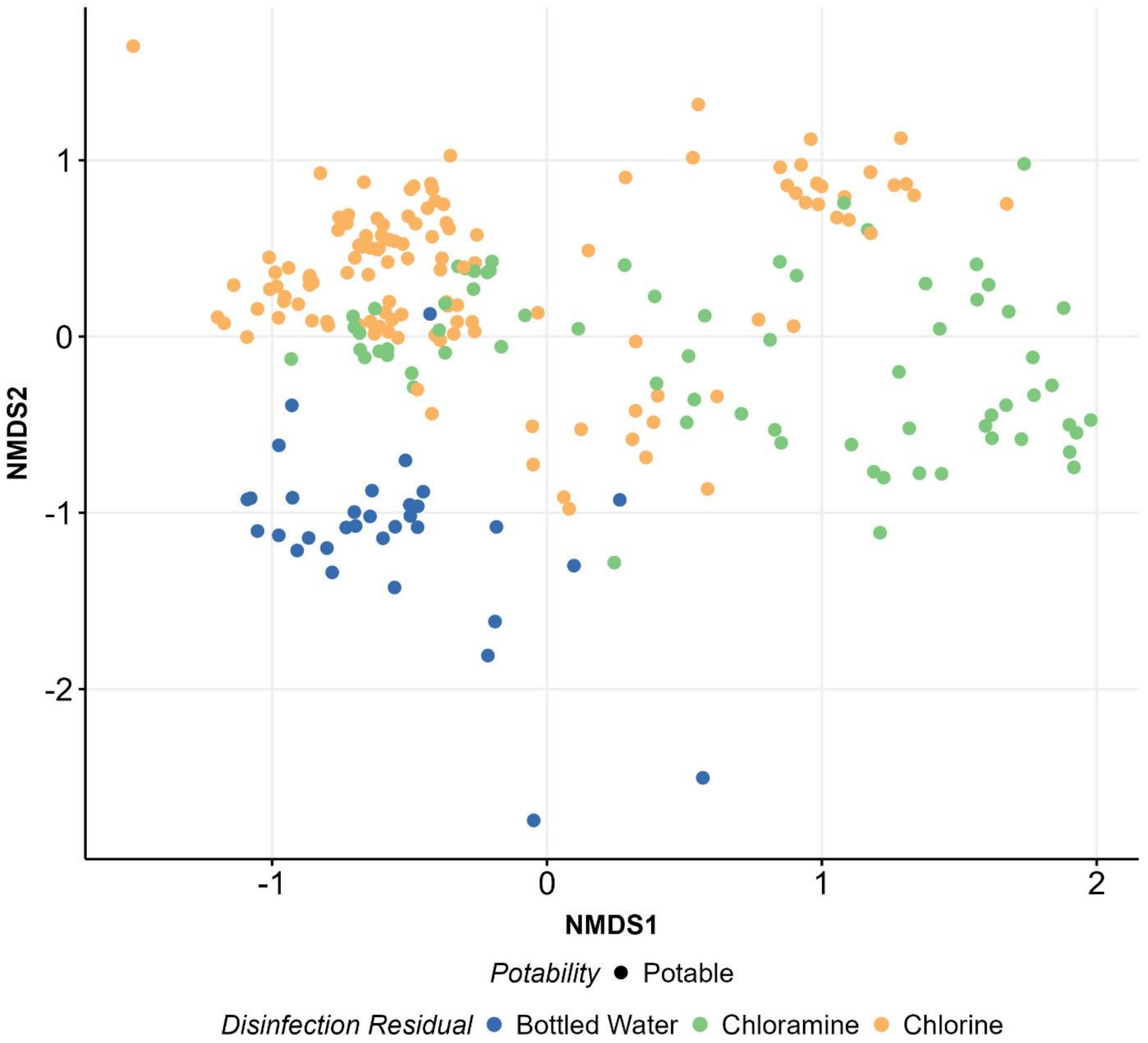

SI Figure 16. Bray-Curtis beta diversity plot for all potable bulk water samples, classified by final disinfection residual.

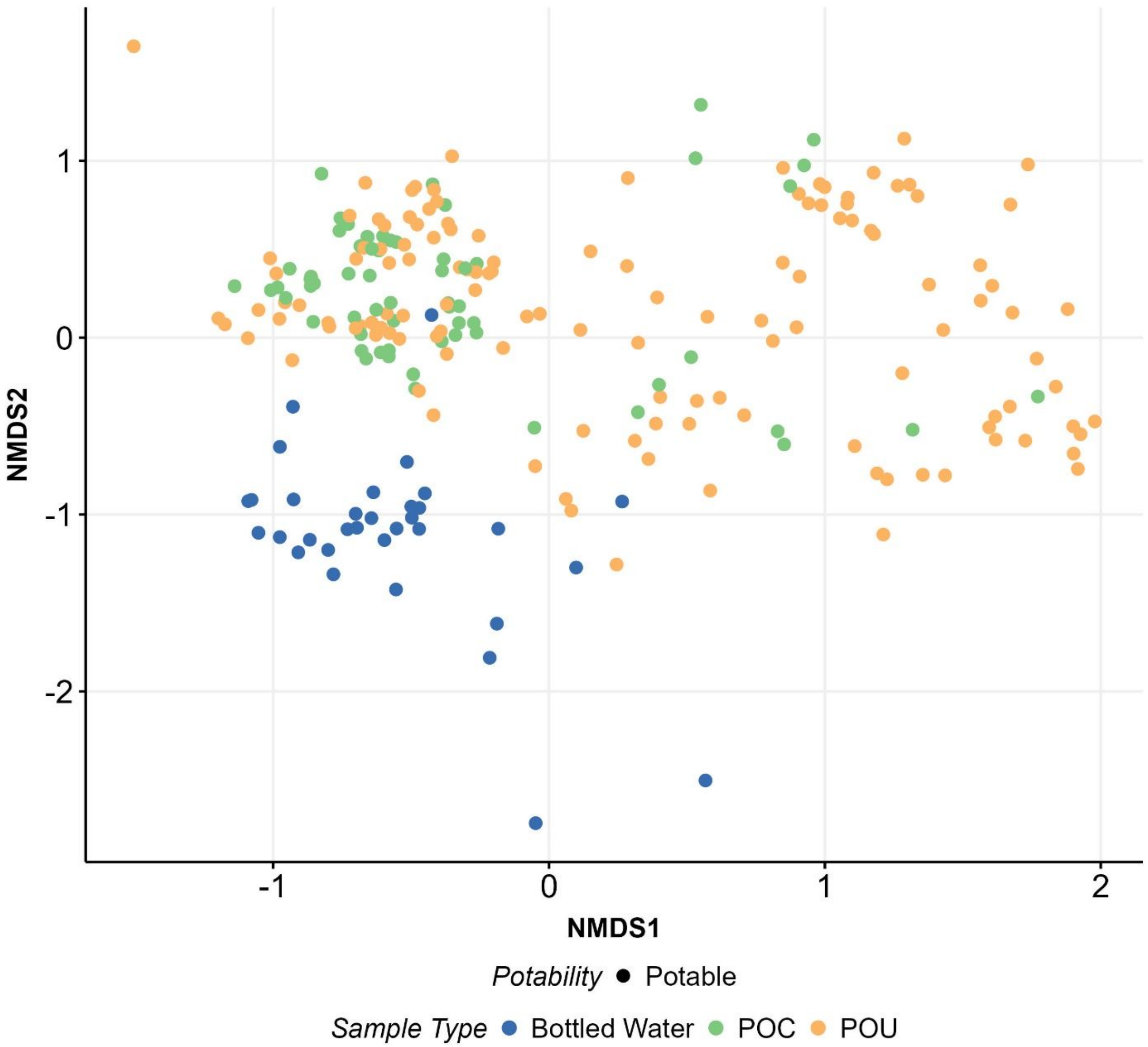

SI Figure 17. Bray-Curtis beta diversity plot for all potable bulk water samples, classified by sample location.

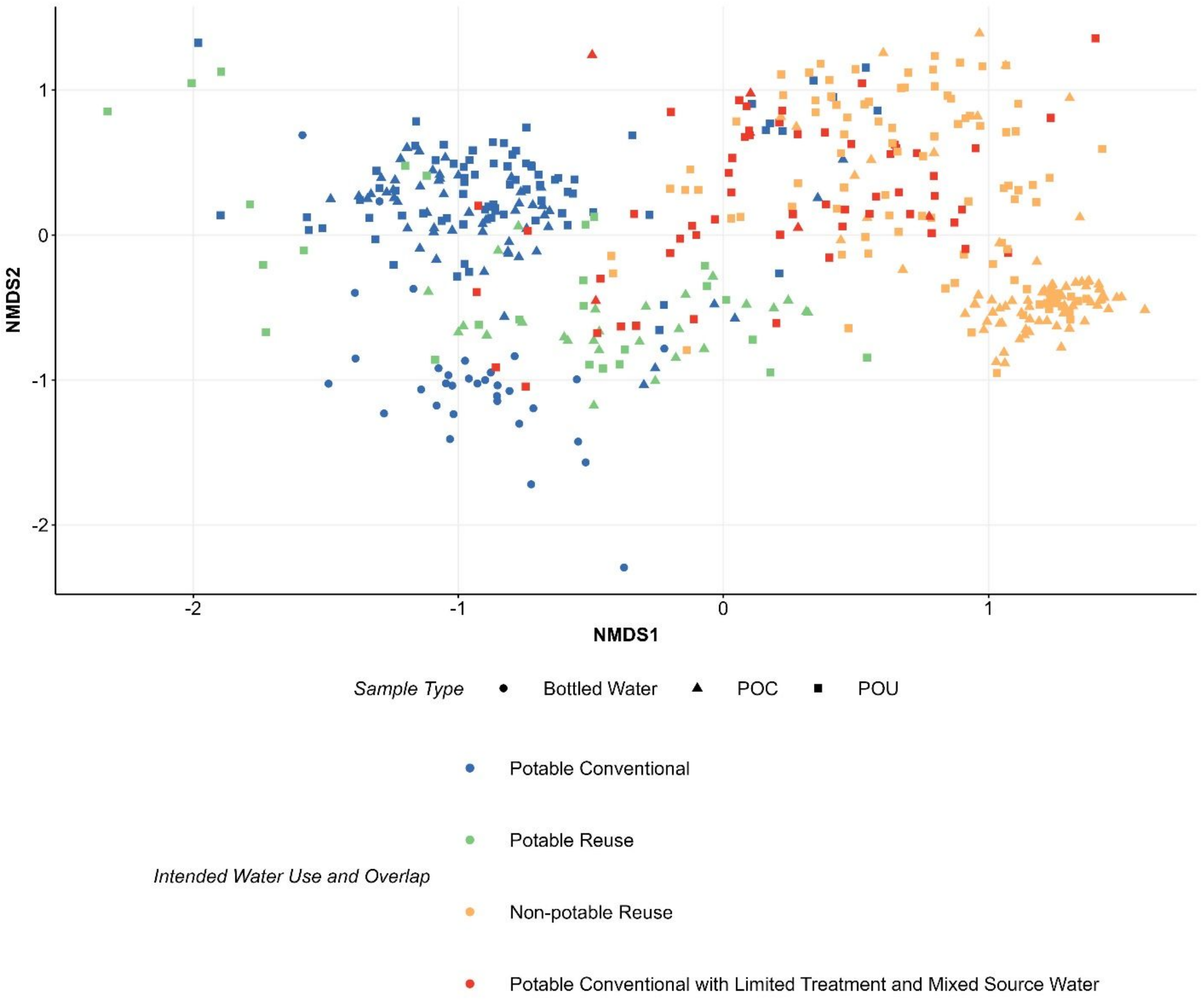

SI Figure 18. Bray-Curtis beta diversity plot for all potable bulk water samples, classified by water use with potable samples subjected to limited treatment and mixed source waters identified. These samples were removed in the modified dataset as identified in the manuscript text.

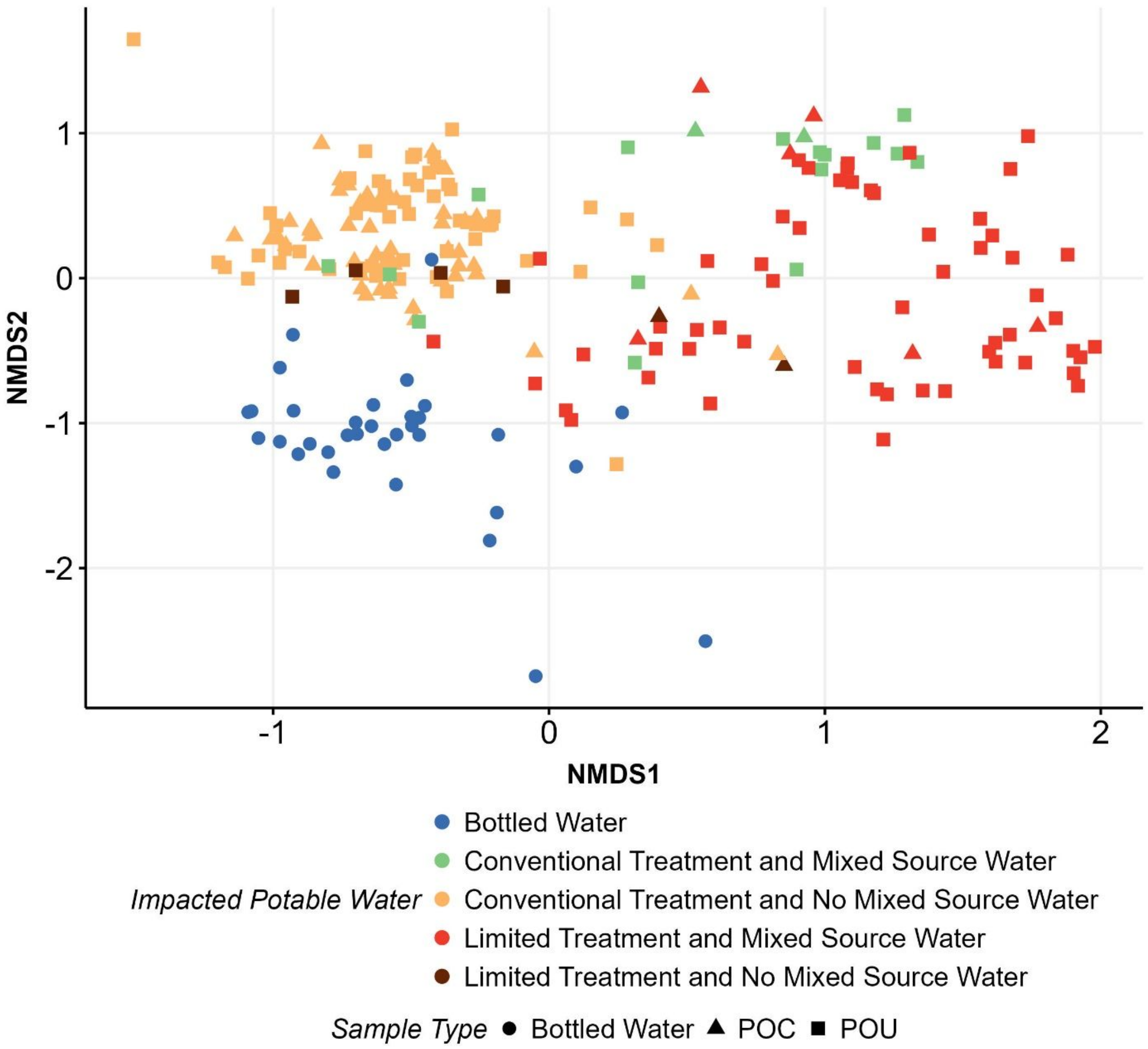

SI Figure 19. Bray-Curtis beta diversity plot for all potable bulk water samples, classified by factors influencing non-potable overlap.

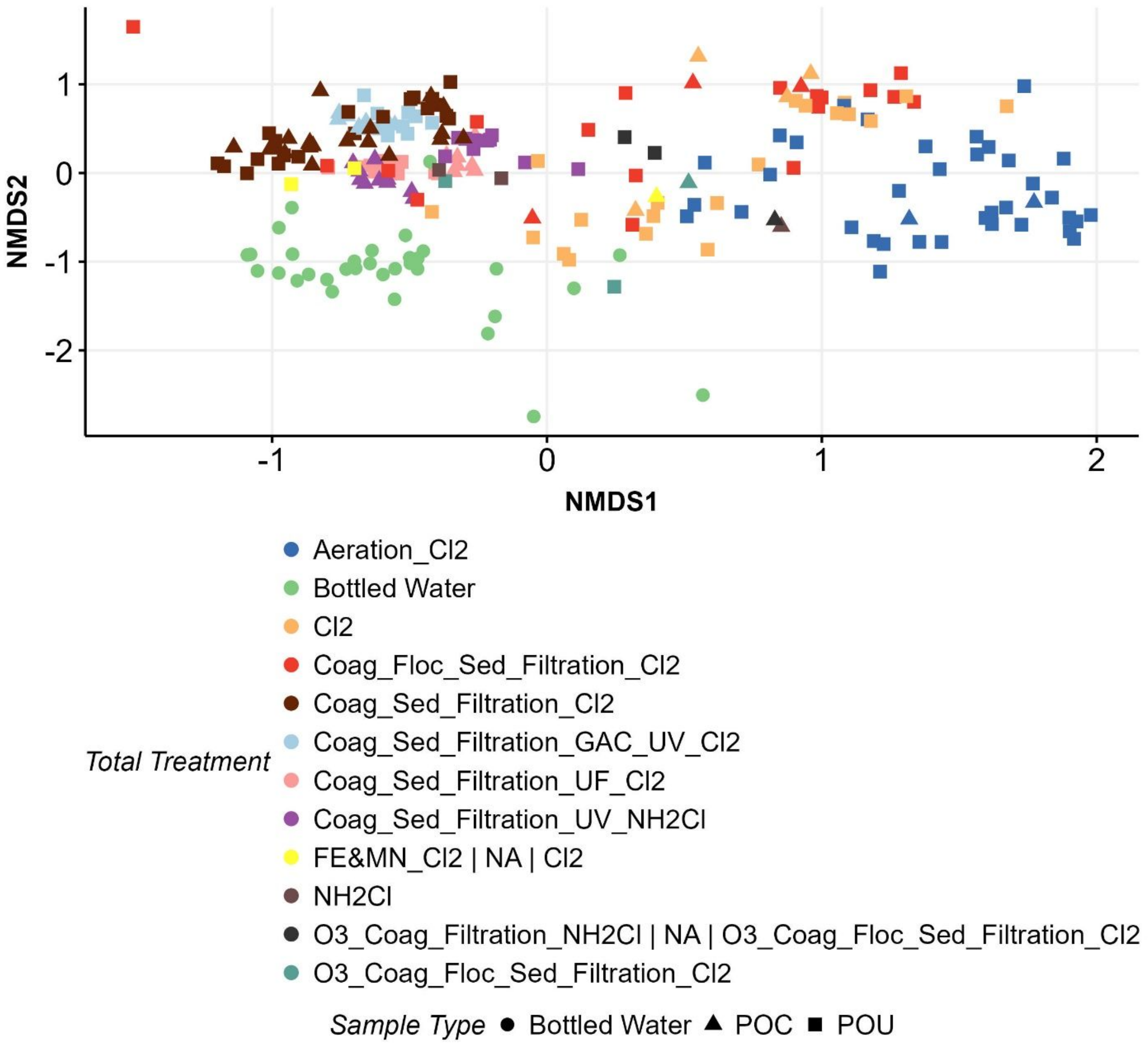

SI Figure 20. Bray-Curtis beta diversity plot for all potable bulk water samples, classified by entire treatment train.

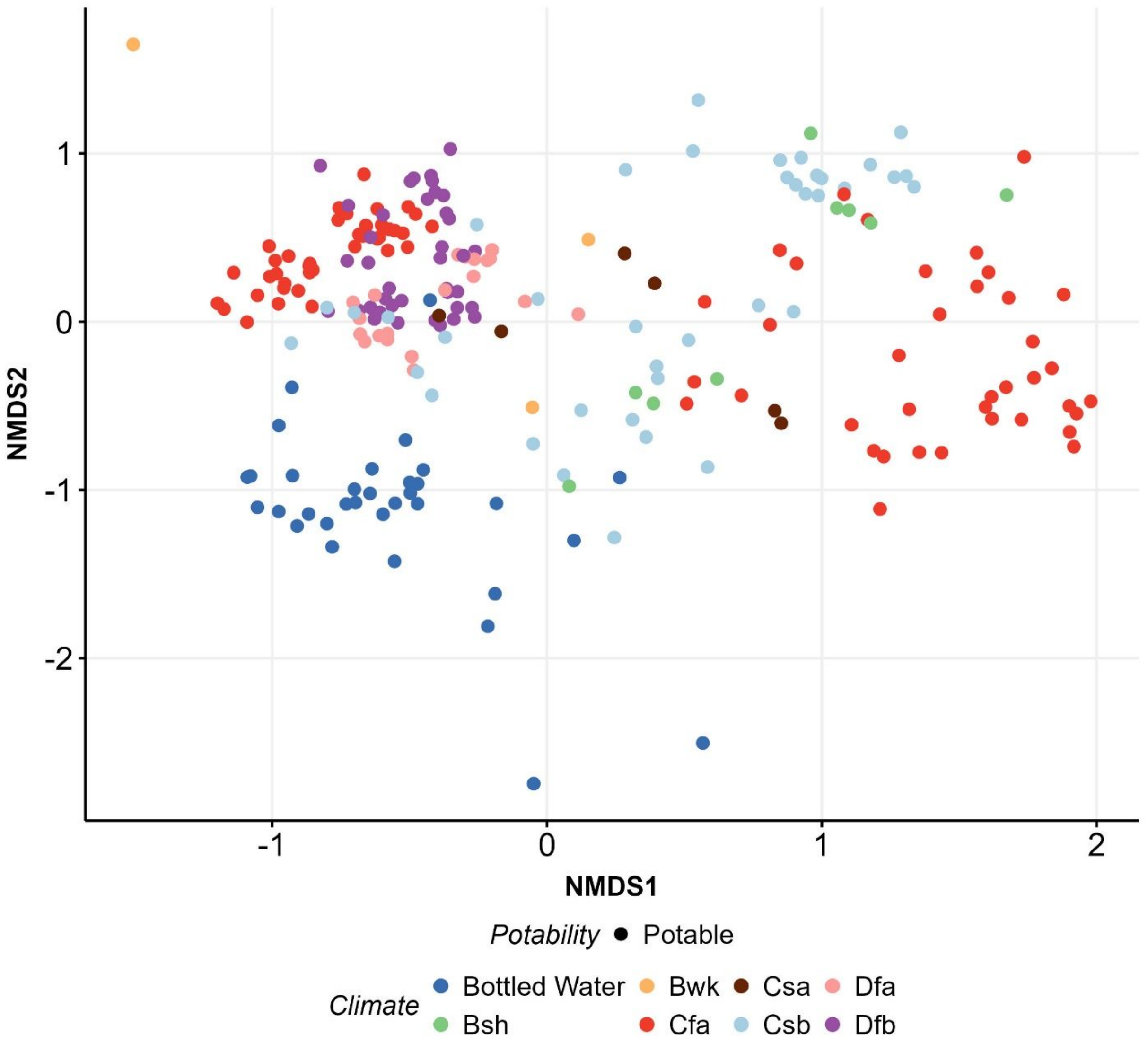

SI Figure 21. Bray-Curtis beta diversity plot for all potable bulk water samples, classified by Köppen climate designator.

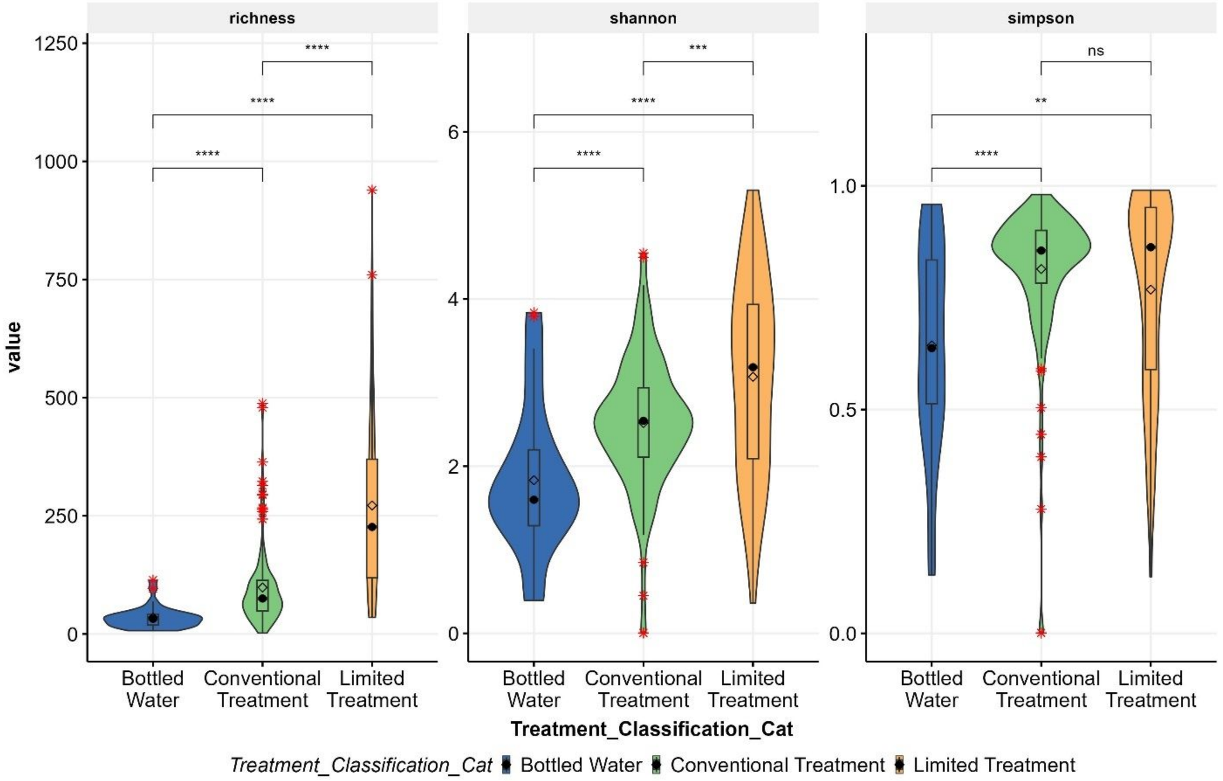

Figure 22. Violin plots of selected alpha diversity metrics for conventional potable samples grouped by categorized treatment. Wilcoxon rank sum tests, with p-value adjustment, were used to test significant differences between comparisons.

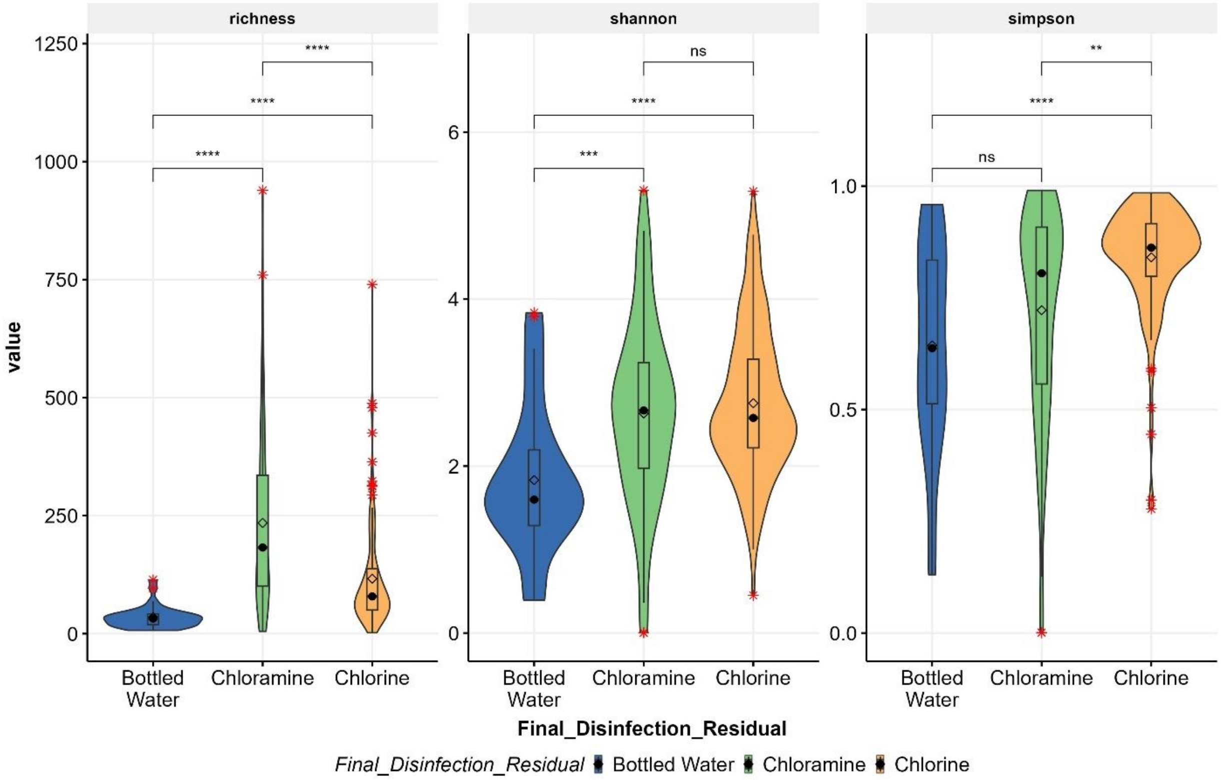

Figure 23. Violin plots of selected alpha diversity metrics for conventional potable samples grouped by disinfection residual. Wilcoxon rank sum tests, with p-value adjustment, were used to test significant differences between comparisons.

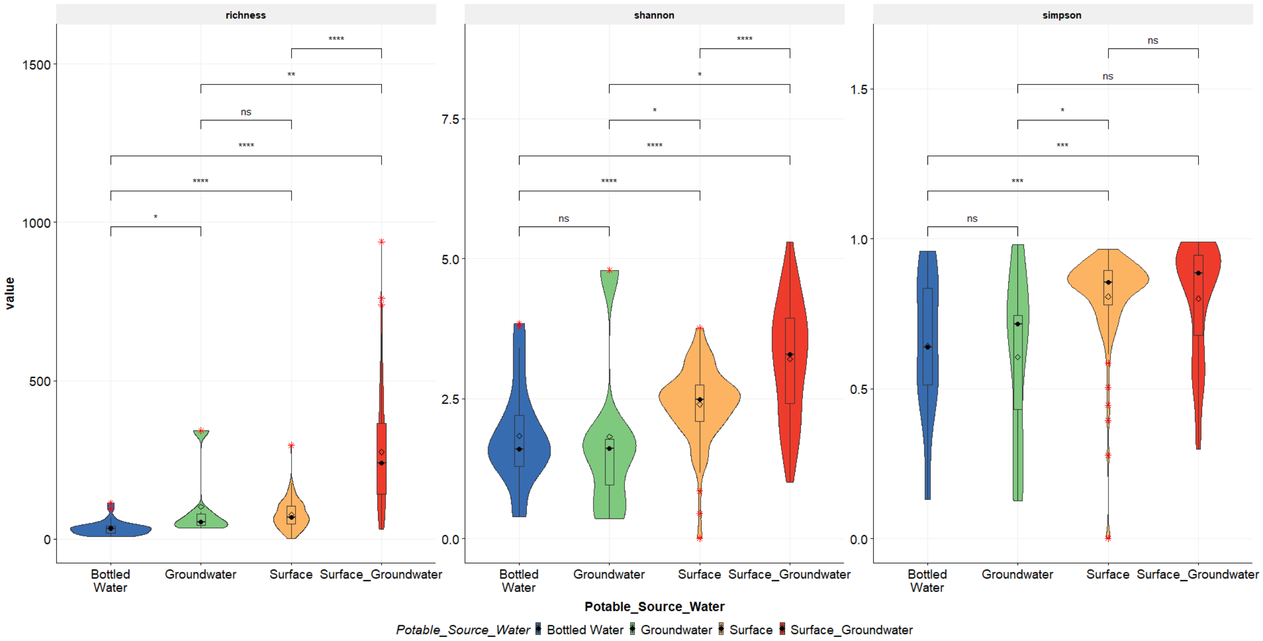

Figure 24. Violin plots of selected alpha diversity metrics for conventional potable samples grouped by initial source water. Wilcoxon rank sum tests, with p-value adjustment, were used to test significant differences between comparisons.

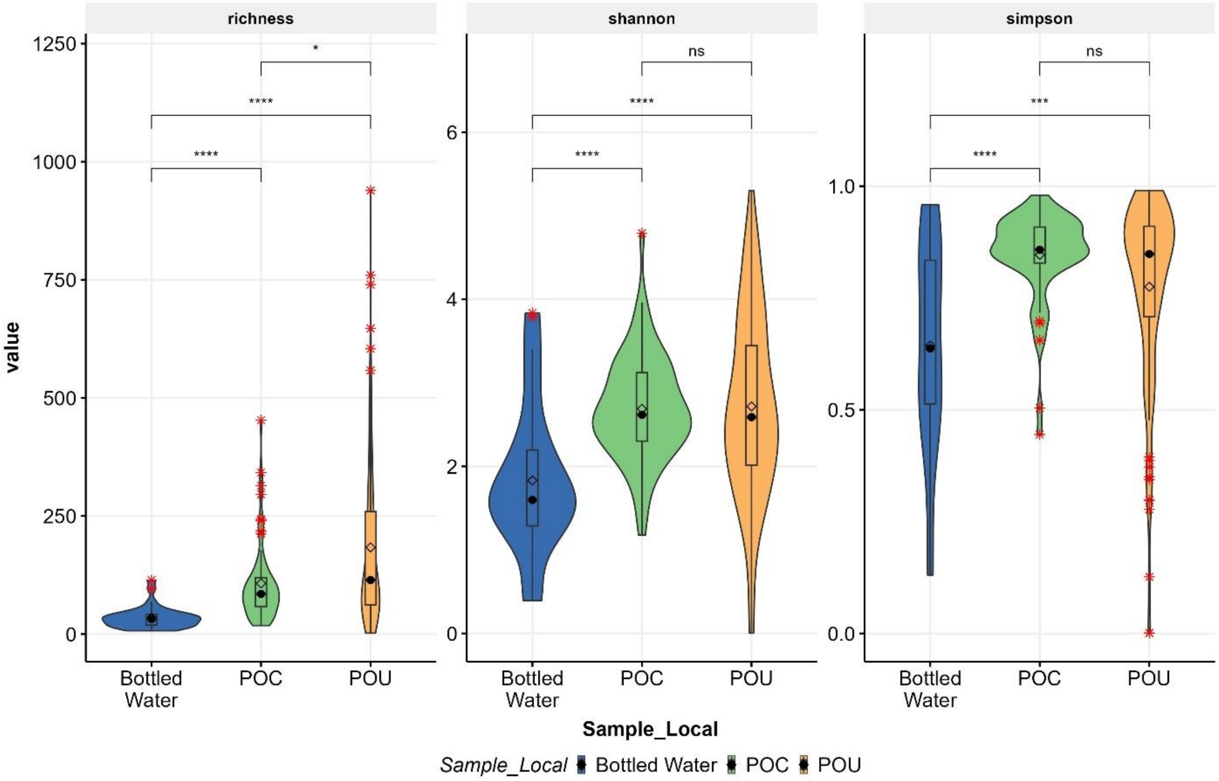

Figure 25. Violin plots of selected alpha diversity metrics for conventional potable samples grouped by sample location. Wilcoxon rank sum tests, with p-value adjustment, were used to test significant differences between comparisons.

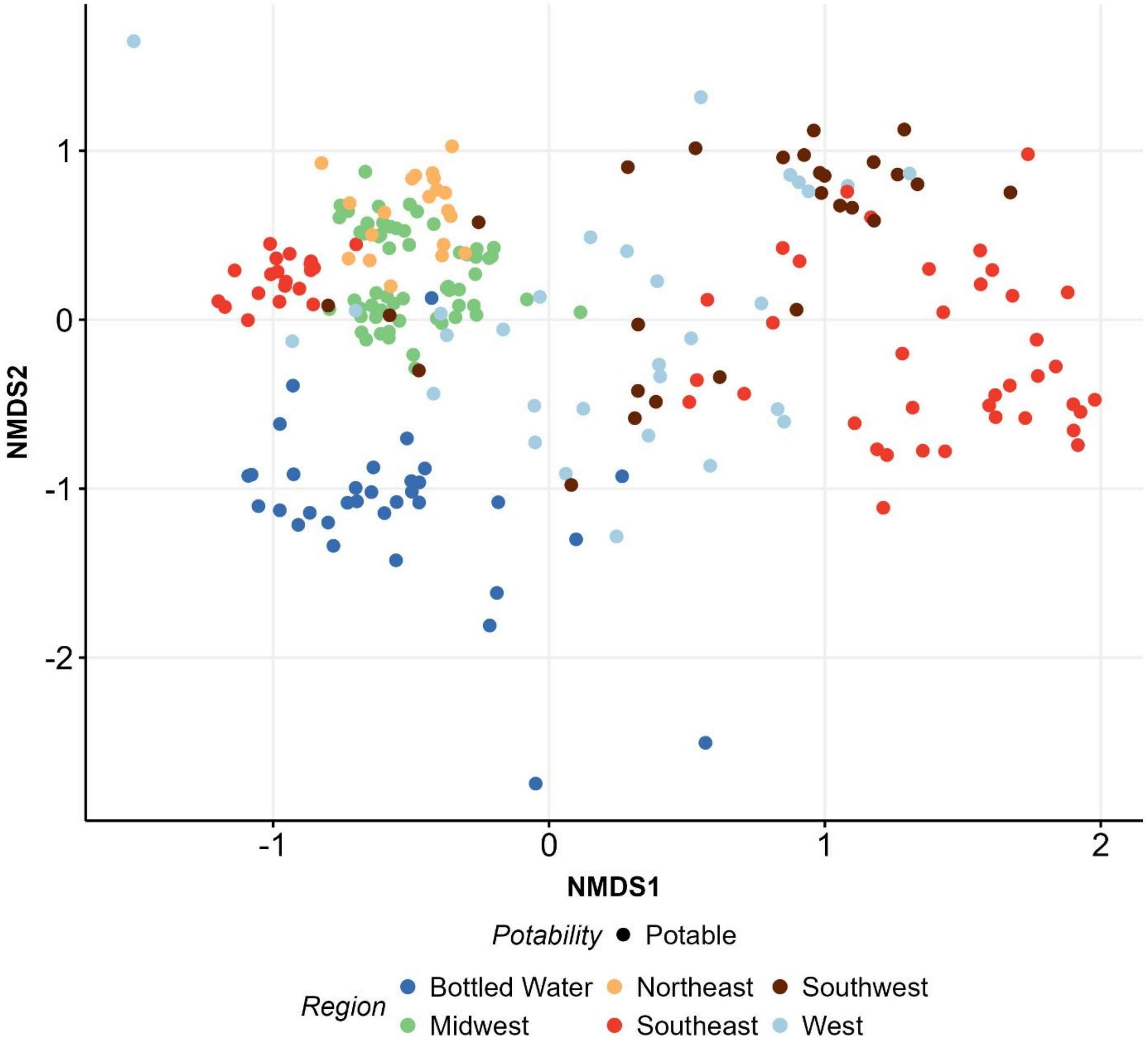

SI Figure 26. Bray-Curtis beta diversity plot for all potable bulk water samples, classified by a regional designator.

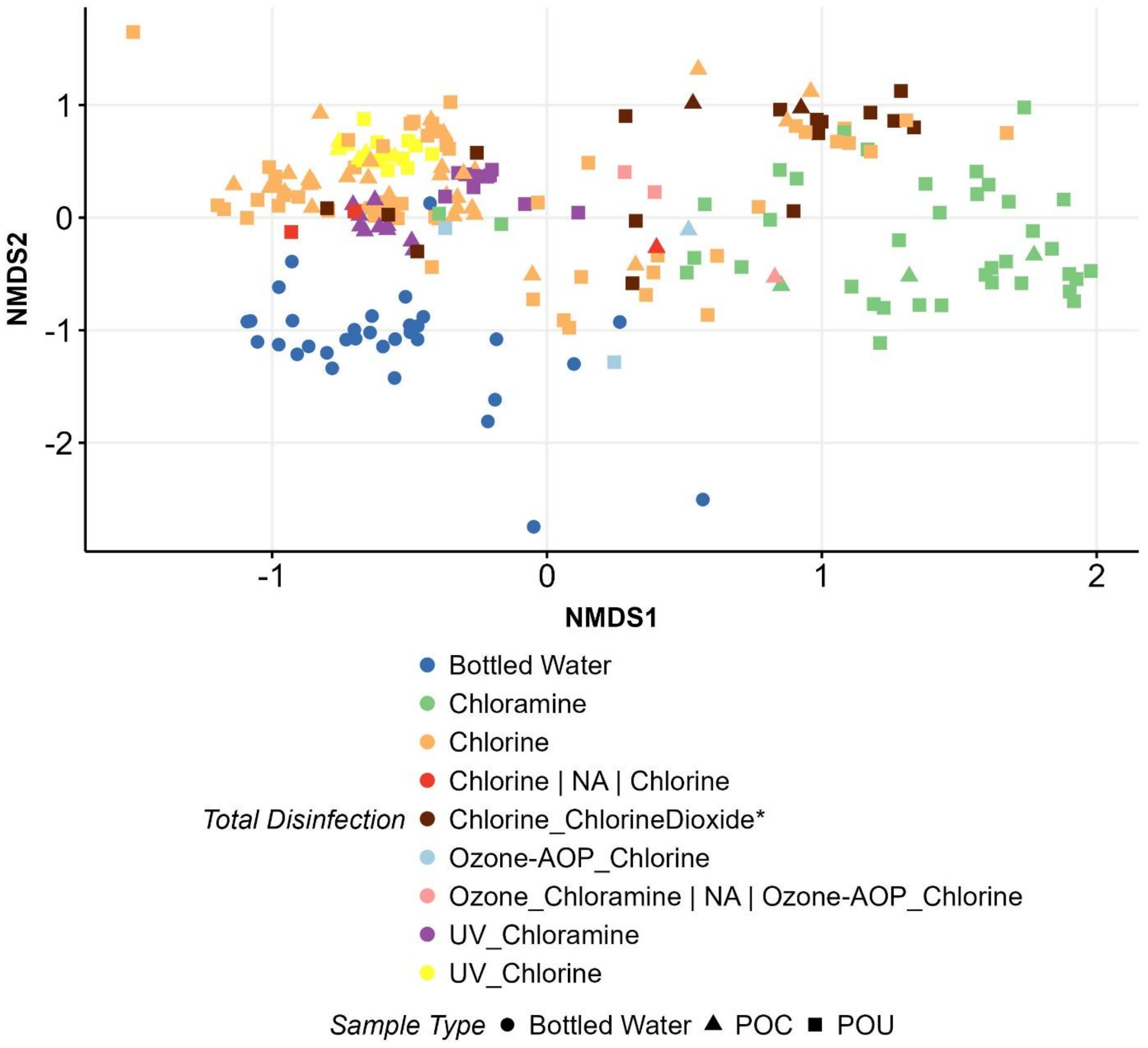

SI Figure 27. Bray-Curtis beta diversity plot for all potable bulk water samples, classified by disinfection processes.

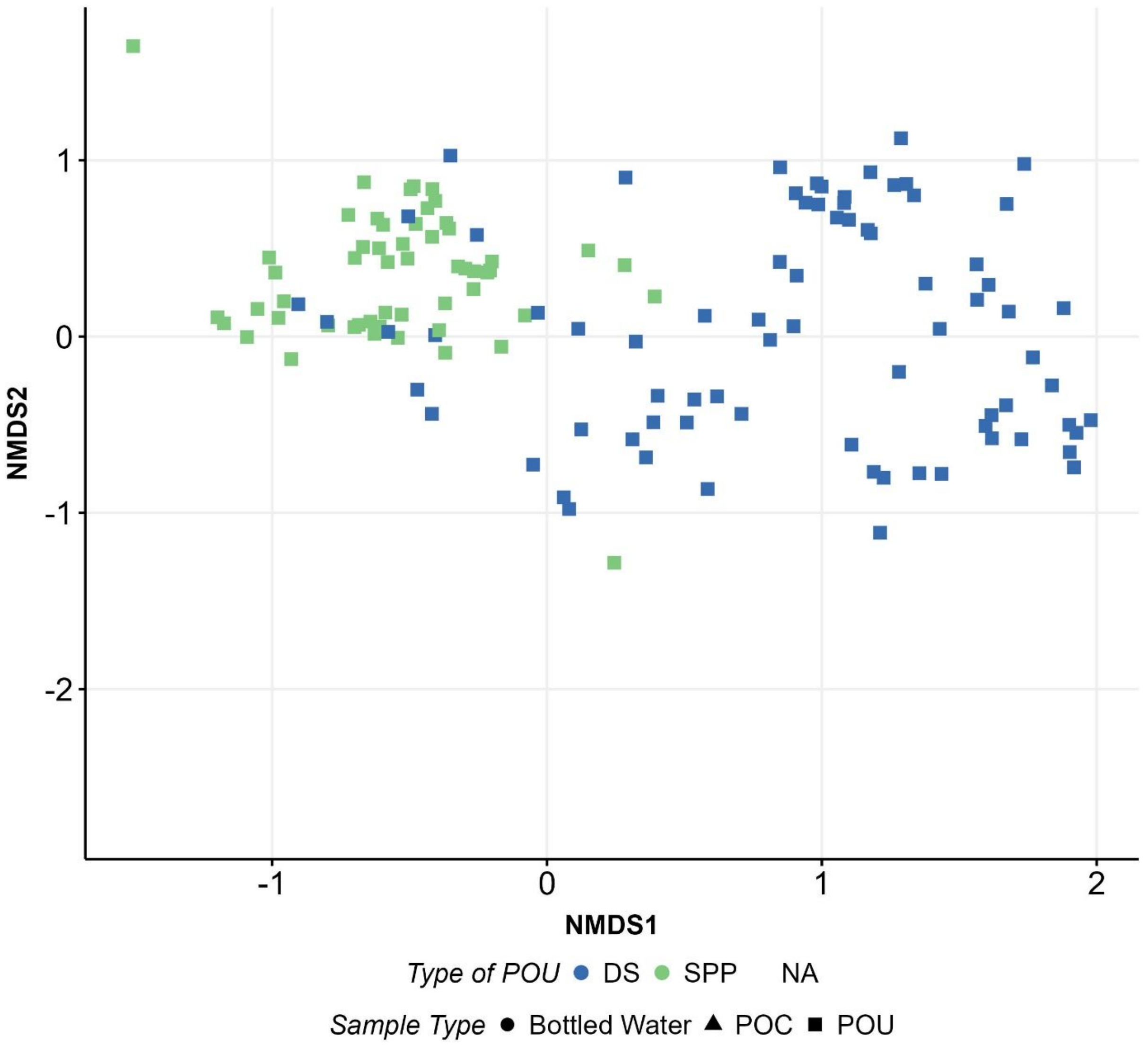

SI Figure 28. Bray-Curtis beta diversity plot for all potable bulk water samples, classified by type of POU.

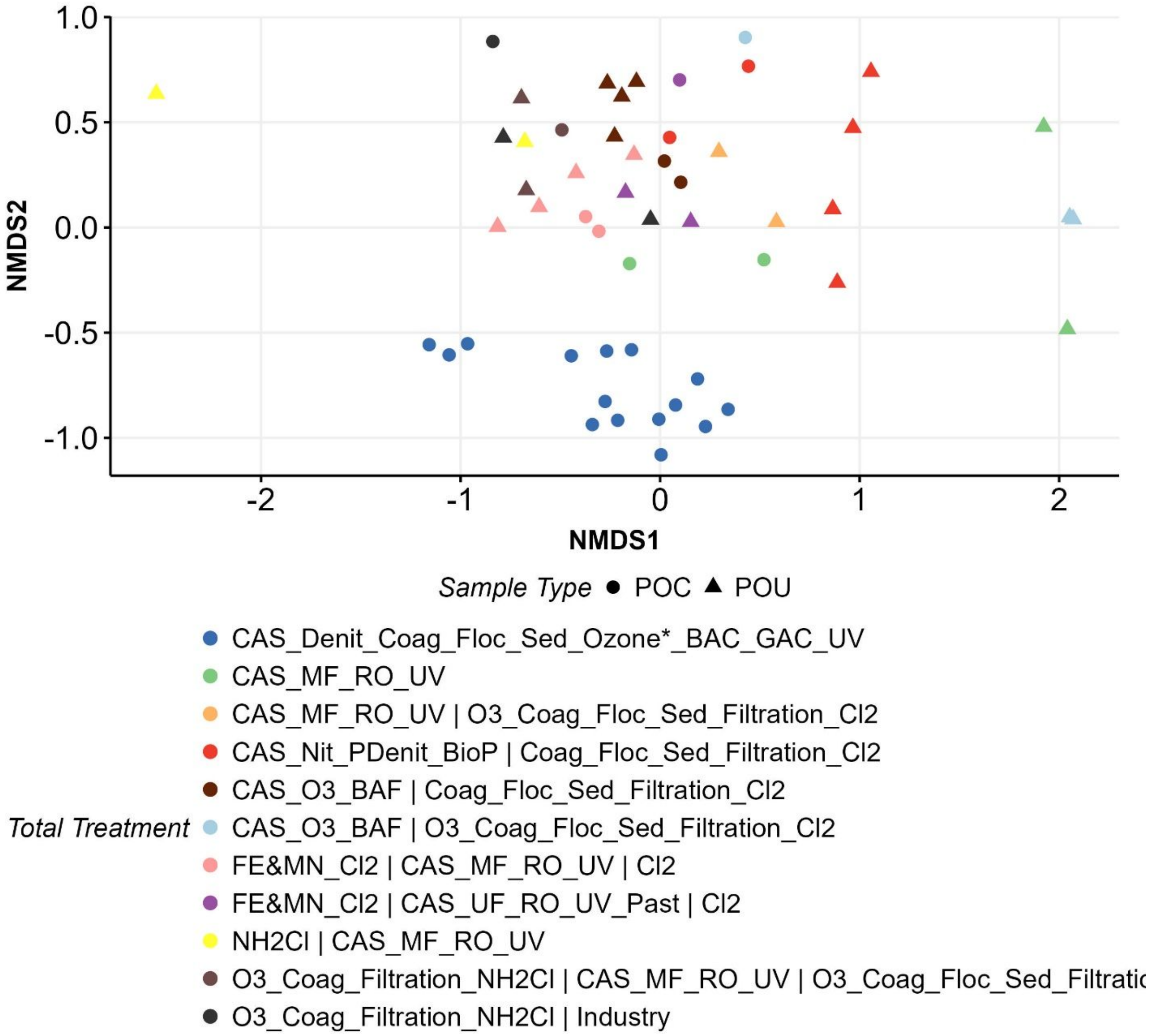

SI Figure 29. Bray-Curtis beta diversity plot for all potable reuse bulk water samples, classified by entire treatment train.

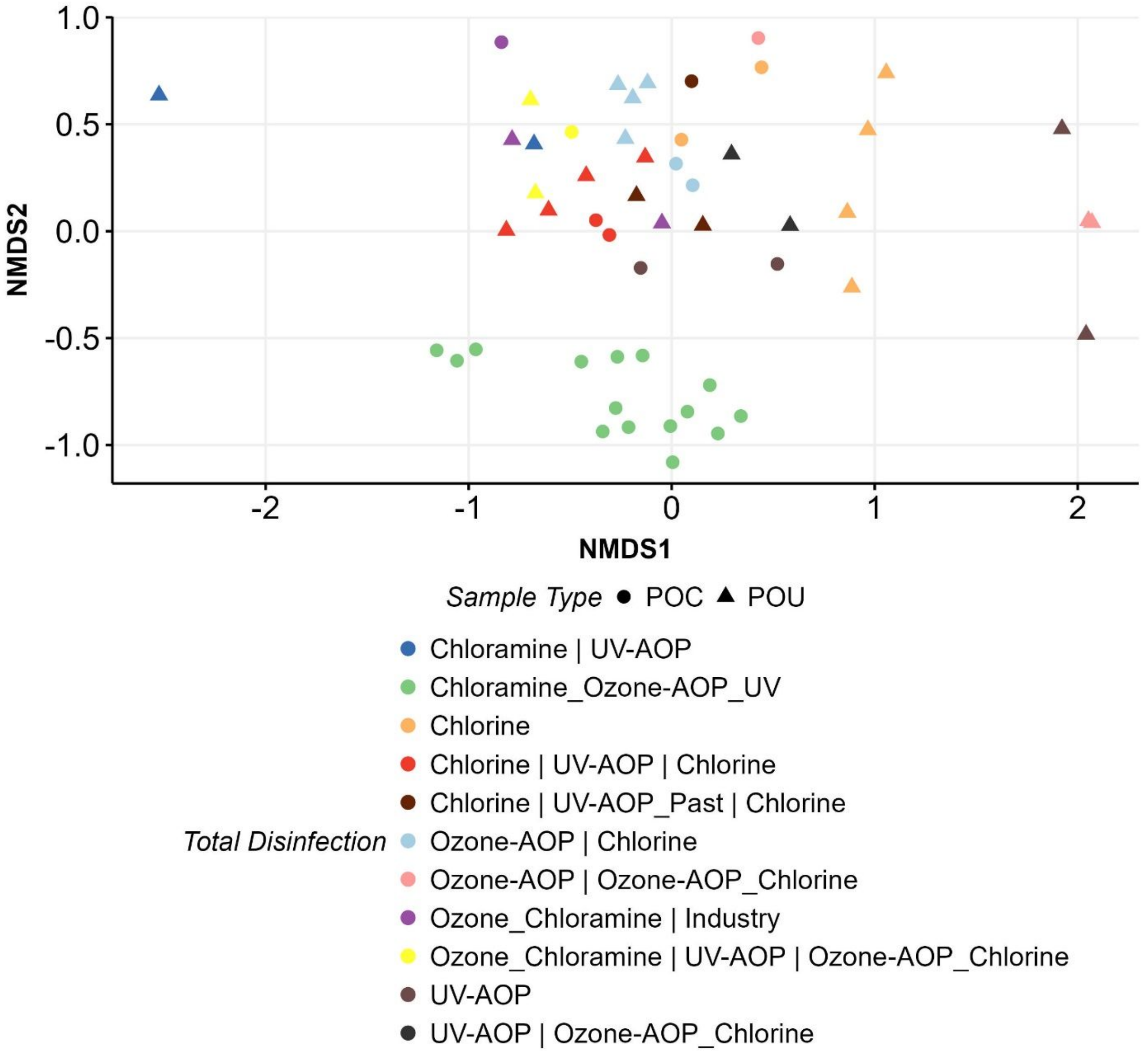

SI Figure 30. Bray-Curtis beta diversity plot for all potable reuse bulk water samples, classified by employed disinfection processes.

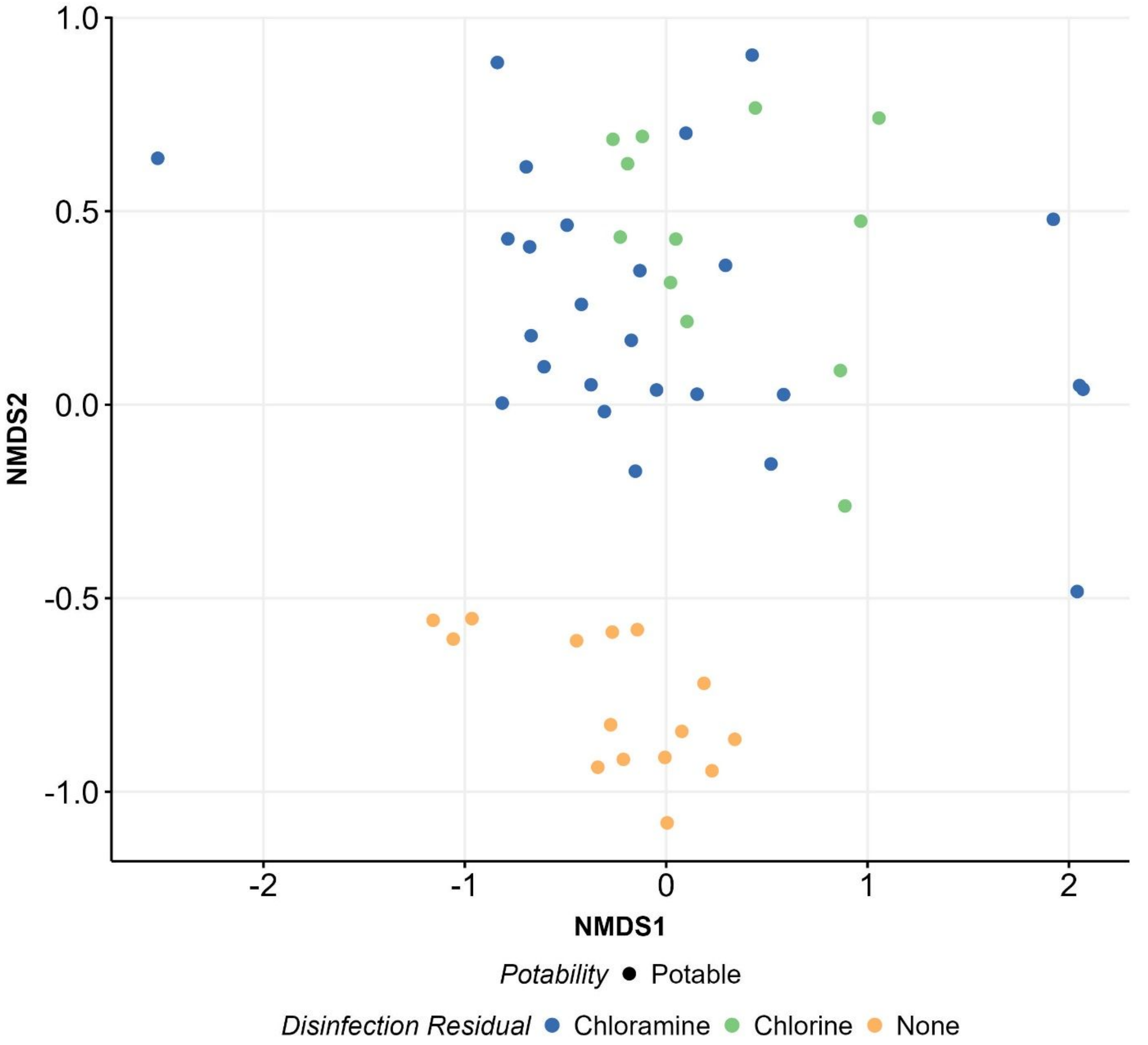

SI Figure 31. Bray-Curtis beta diversity plot for all potable reuse bulk water samples, classified by residual disinfectant.

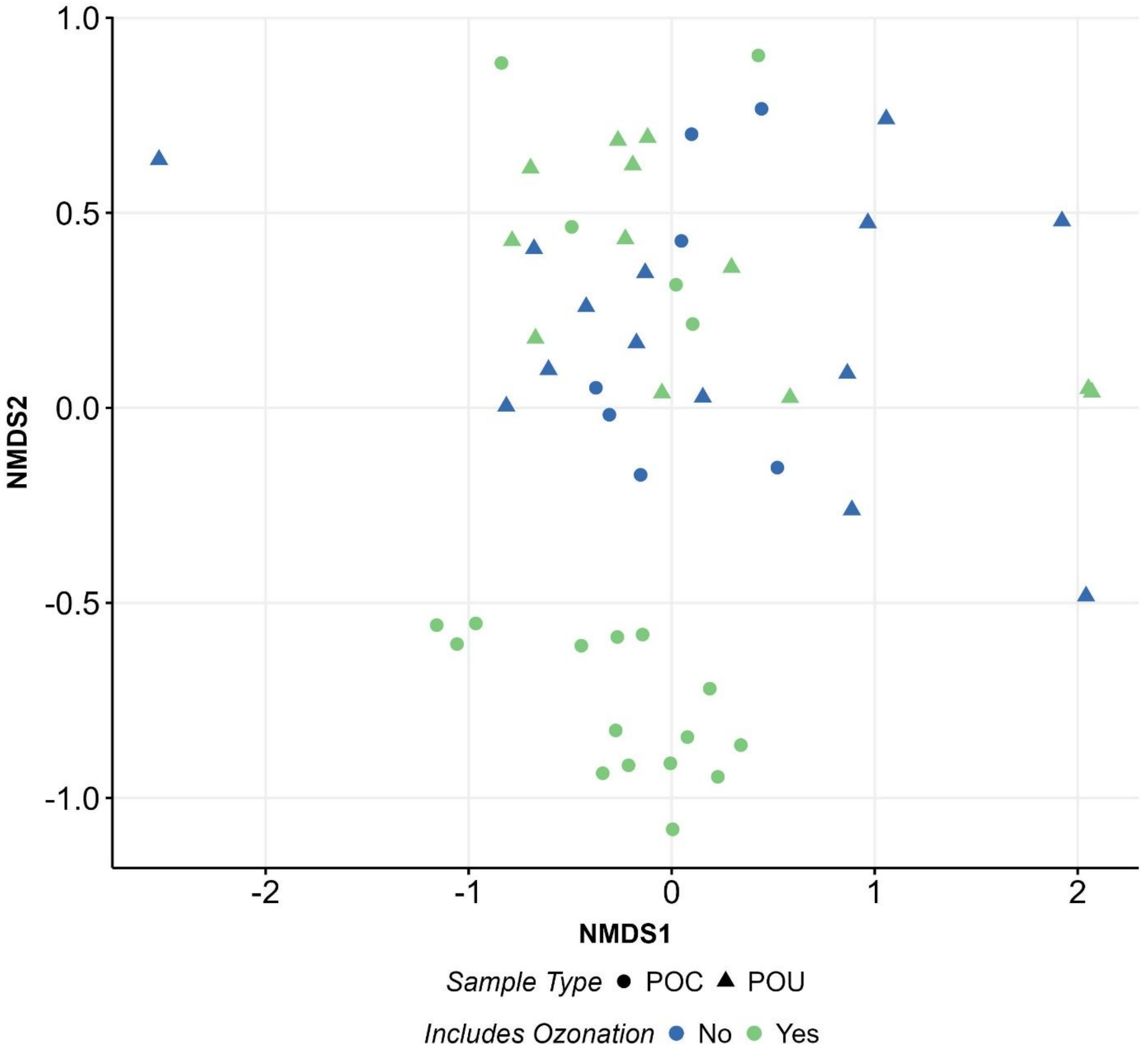

SI Figure 32. Bray-Curtis beta diversity plot for all potable reuse bulk water samples, classified by the inclusion of an ozone treatment process.

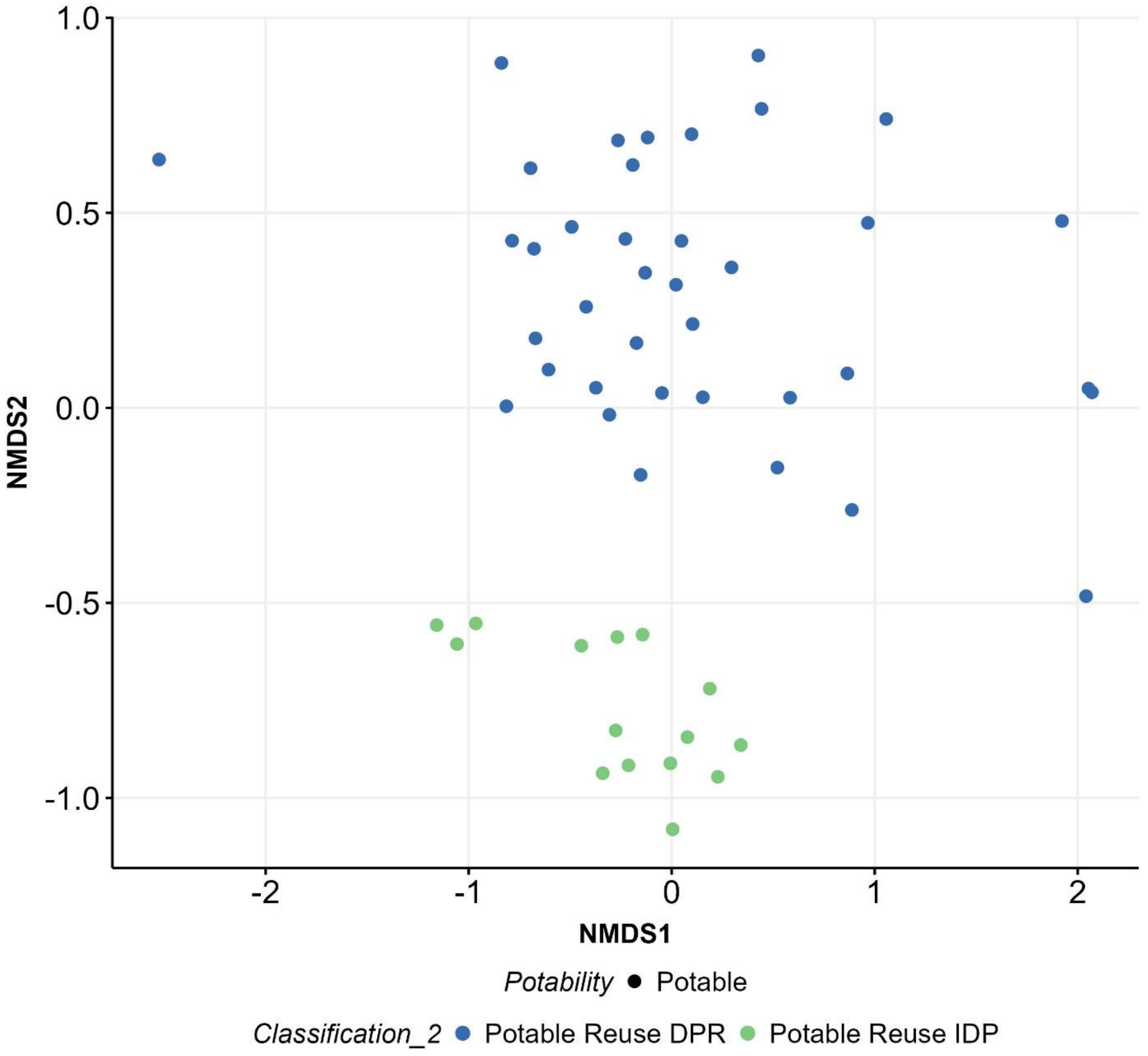

SI Figure 33. Bray-Curtis beta diversity plot for all potable reuse bulk water samples, classified by the distinction between DPR and IDR systems.

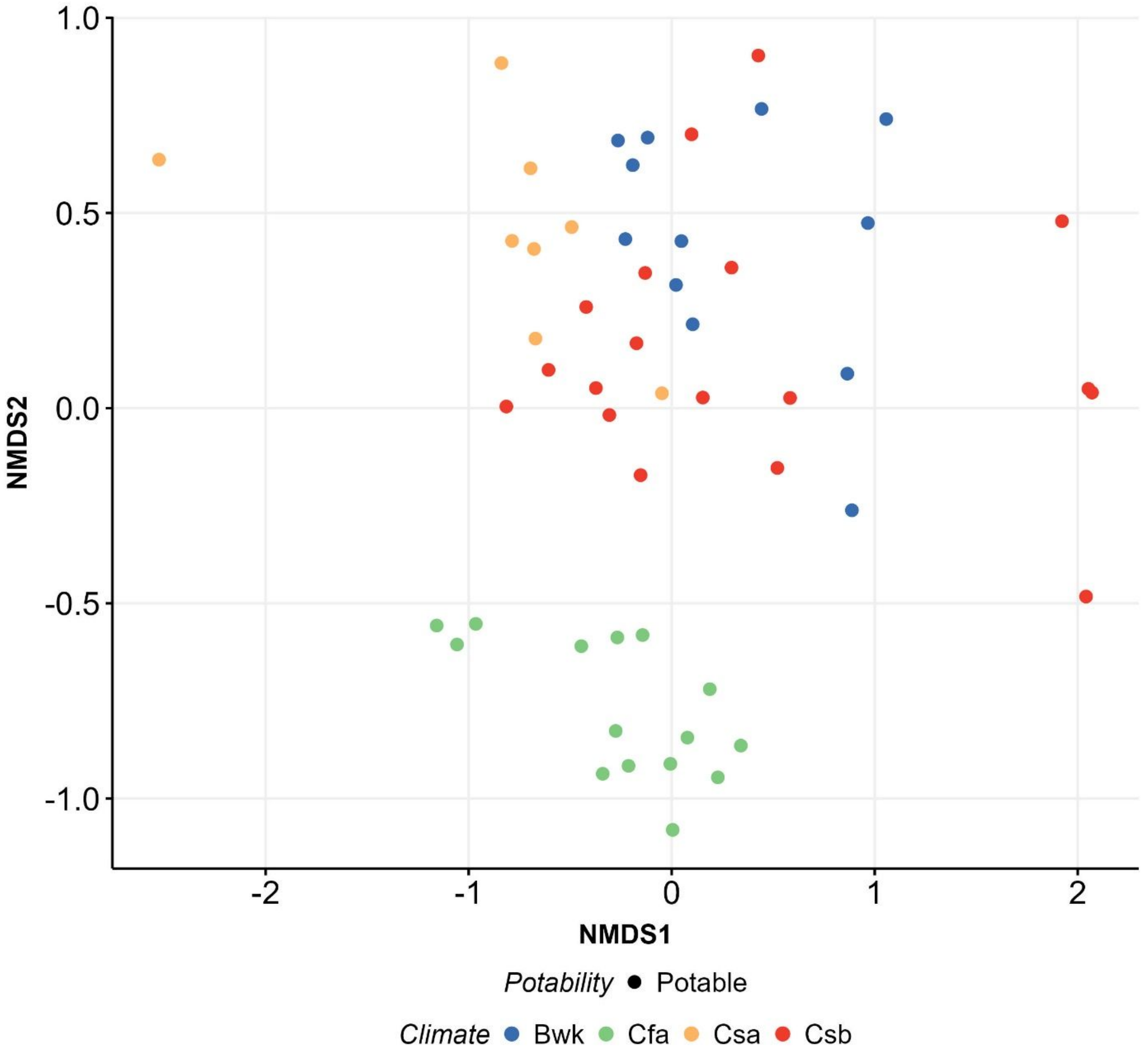

SI Figure 34. Bray-Curtis beta diversity plot for all potable reuse bulk water samples, classified by climate designators.

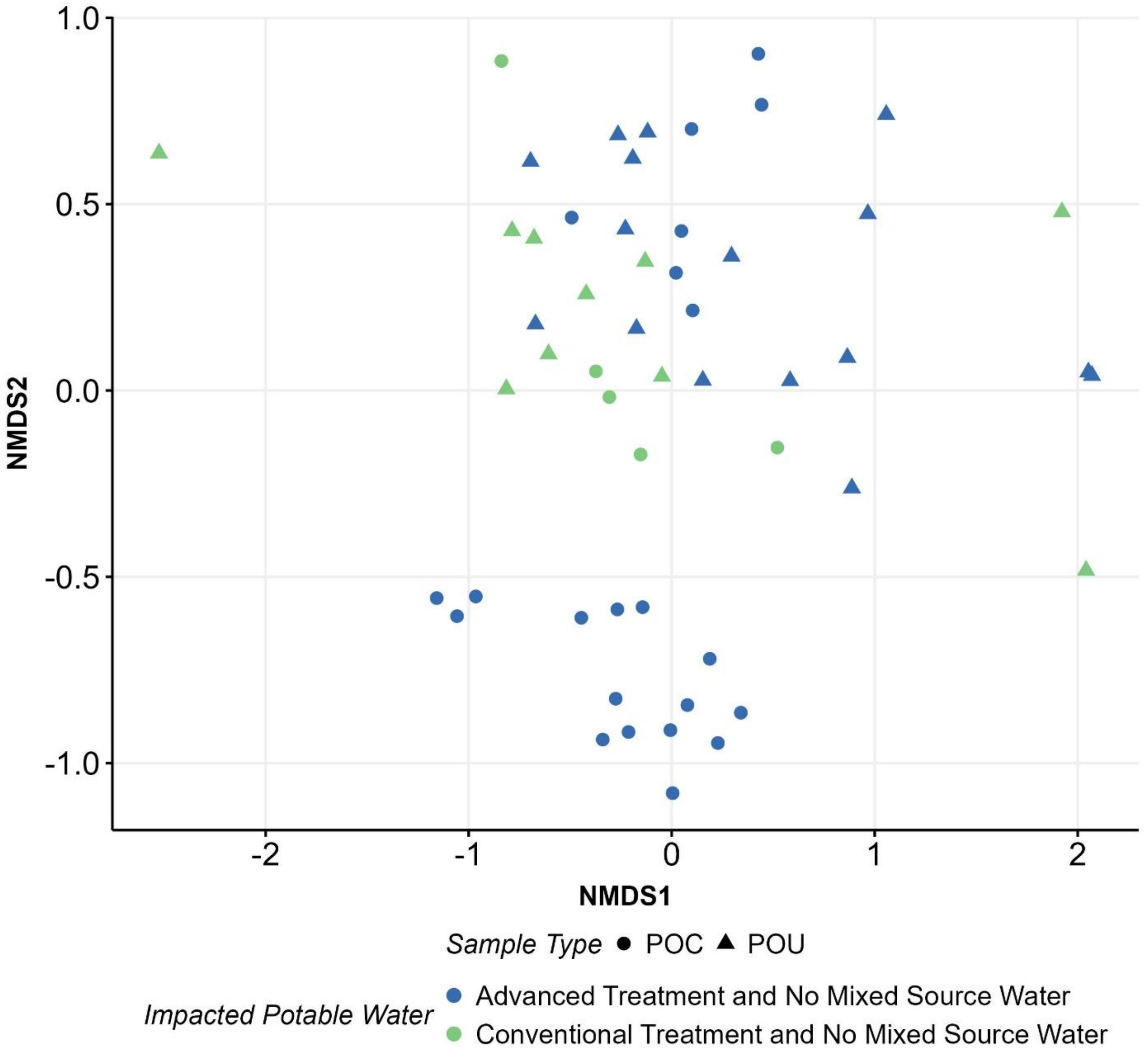

SI Figure 35. Bray-Curtis beta diversity plot for all potable reuse bulk water samples, classified by categorical classification of treatment.

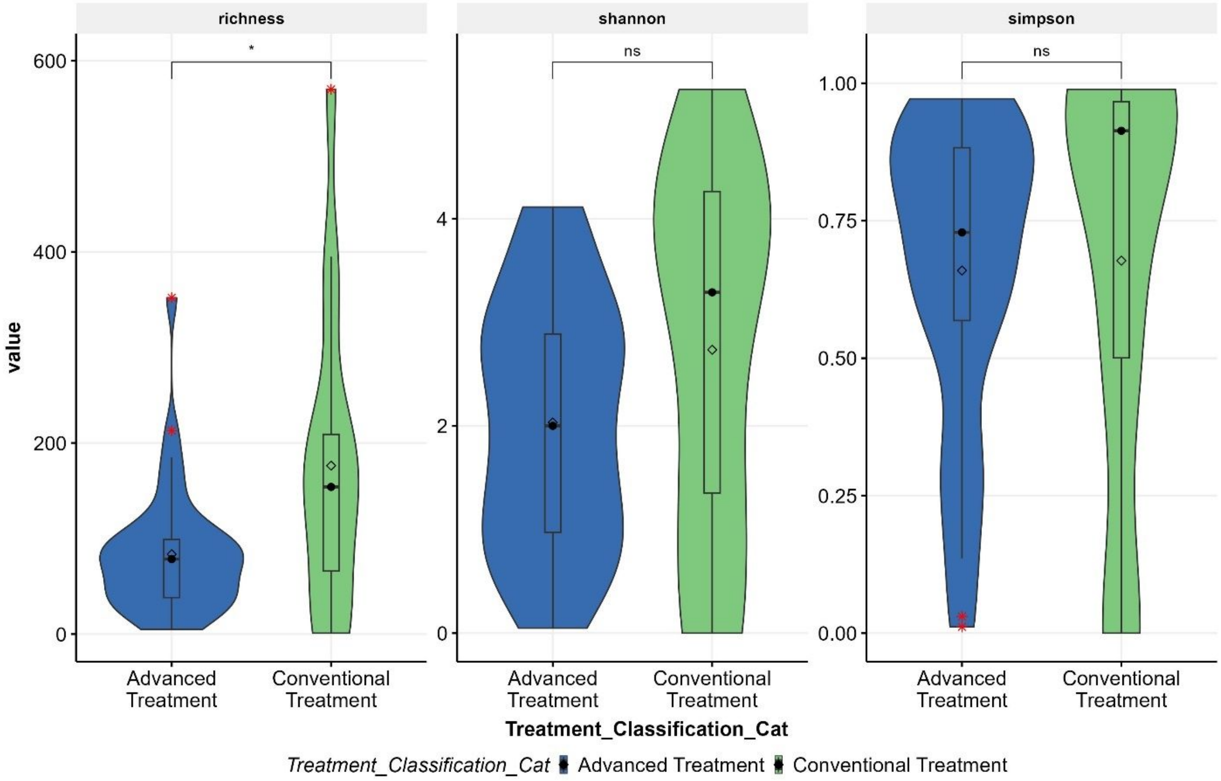

Figure 36. Violin plots of selected alpha diversity metrics for potable reuse samples grouped by categorized treatment. Wilcoxon rank sum tests, with p-value adjustment, were used to test significant differences between comparisons.

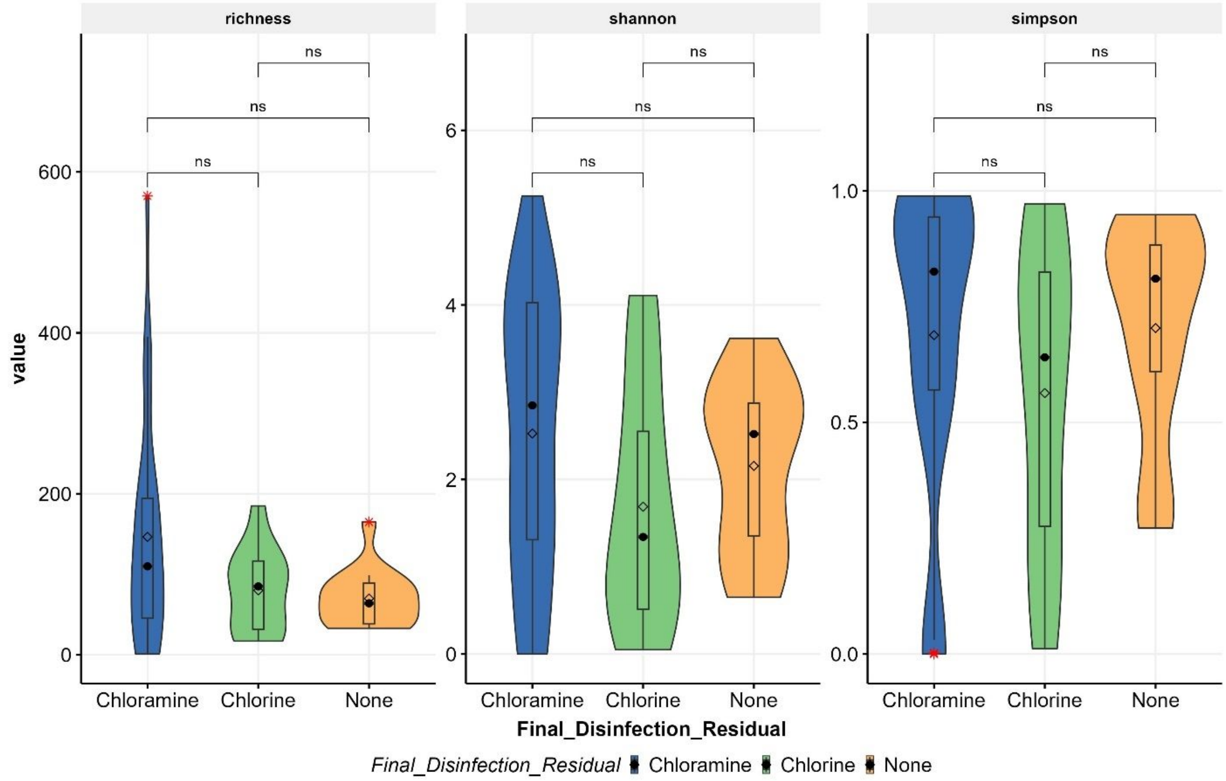

Figure 37. Violin plots of selected alpha diversity metrics for potable reuse samples grouped by disinfection residual. Wilcoxon rank sum tests, with p-value adjustment, were used to test significant differences between comparisons.

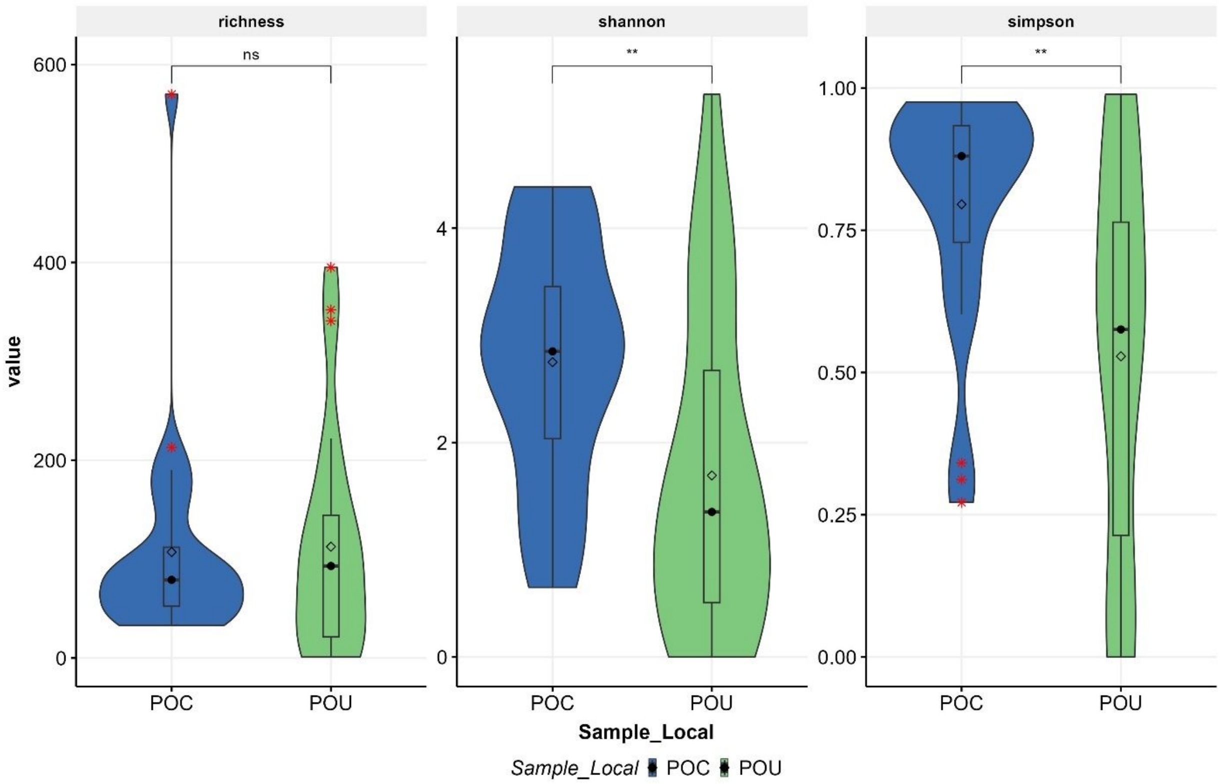

Figure 38. Violin plots of selected alpha diversity metrics for potable reuse samples grouped by sample location. Wilcoxon rank sum tests, with p-value adjustment, were used to test significant differences between comparisons.

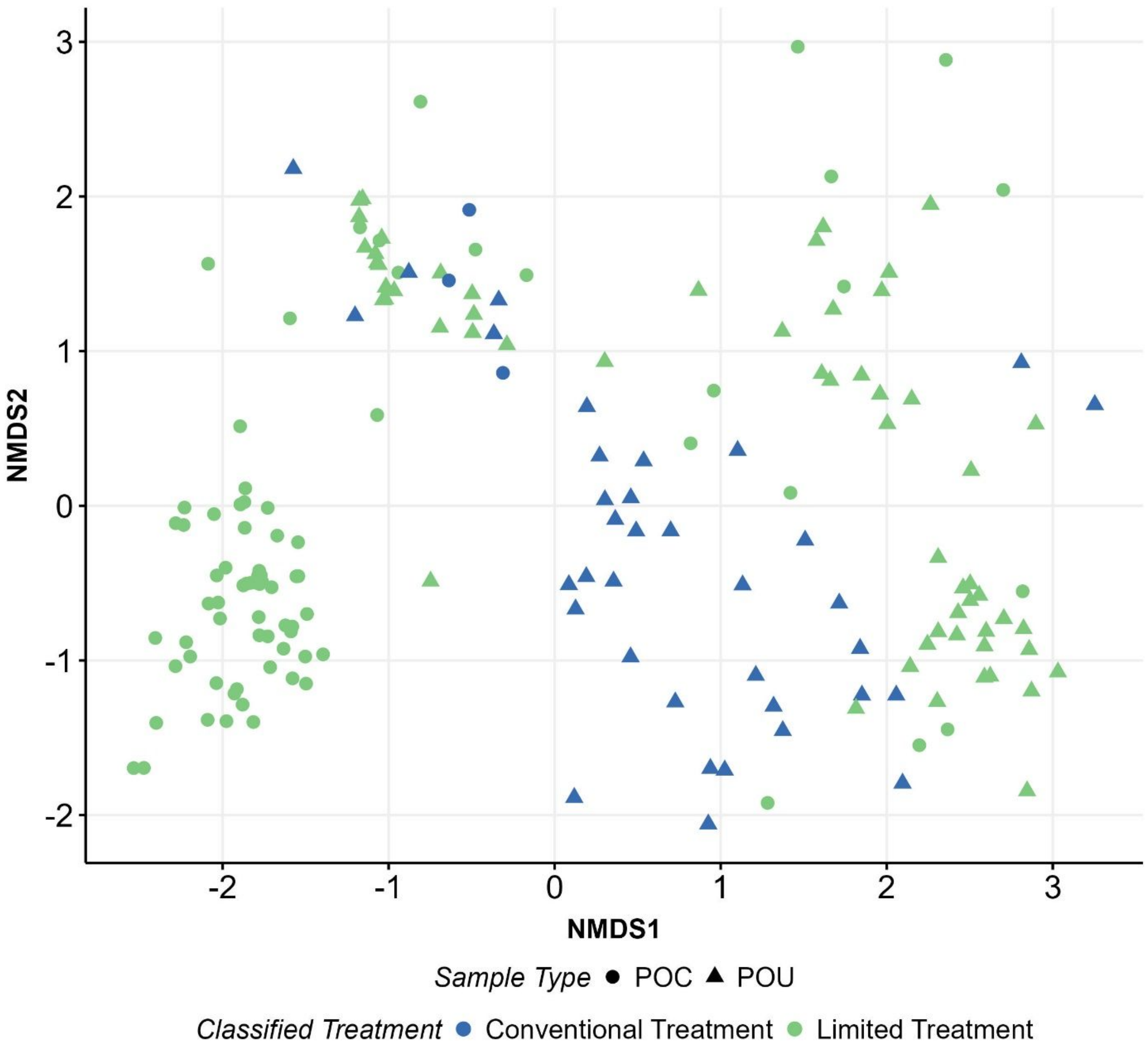

SI Figure 39. Bray-Curtis beta diversity plot for all non-potable reuse bulk water samples, classified by categorical classification of treatment.

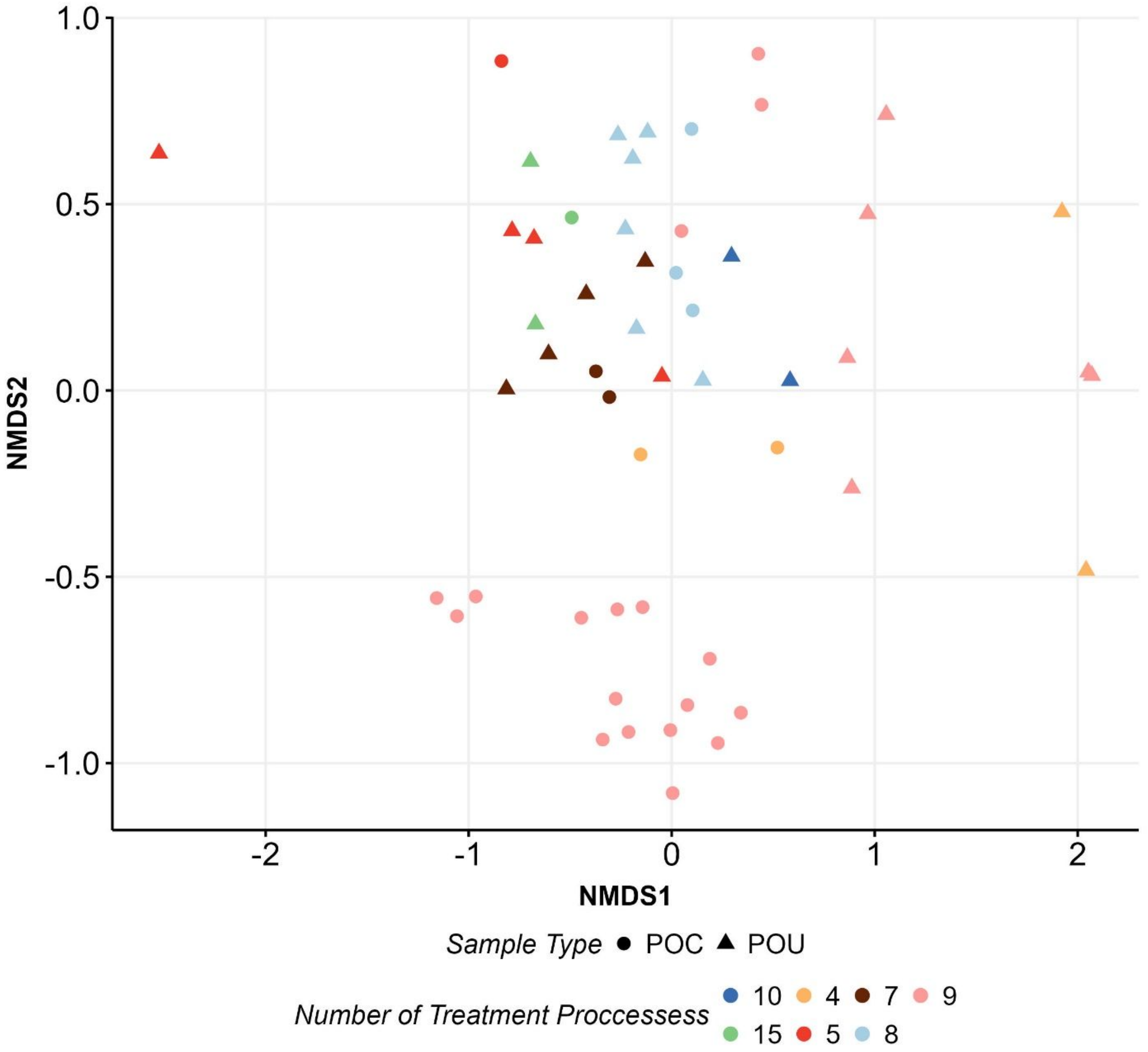

SI Figure 40. Bray-Curtis beta diversity plot for all potable reuse bulk water samples, classified by numerical classification of treatment.

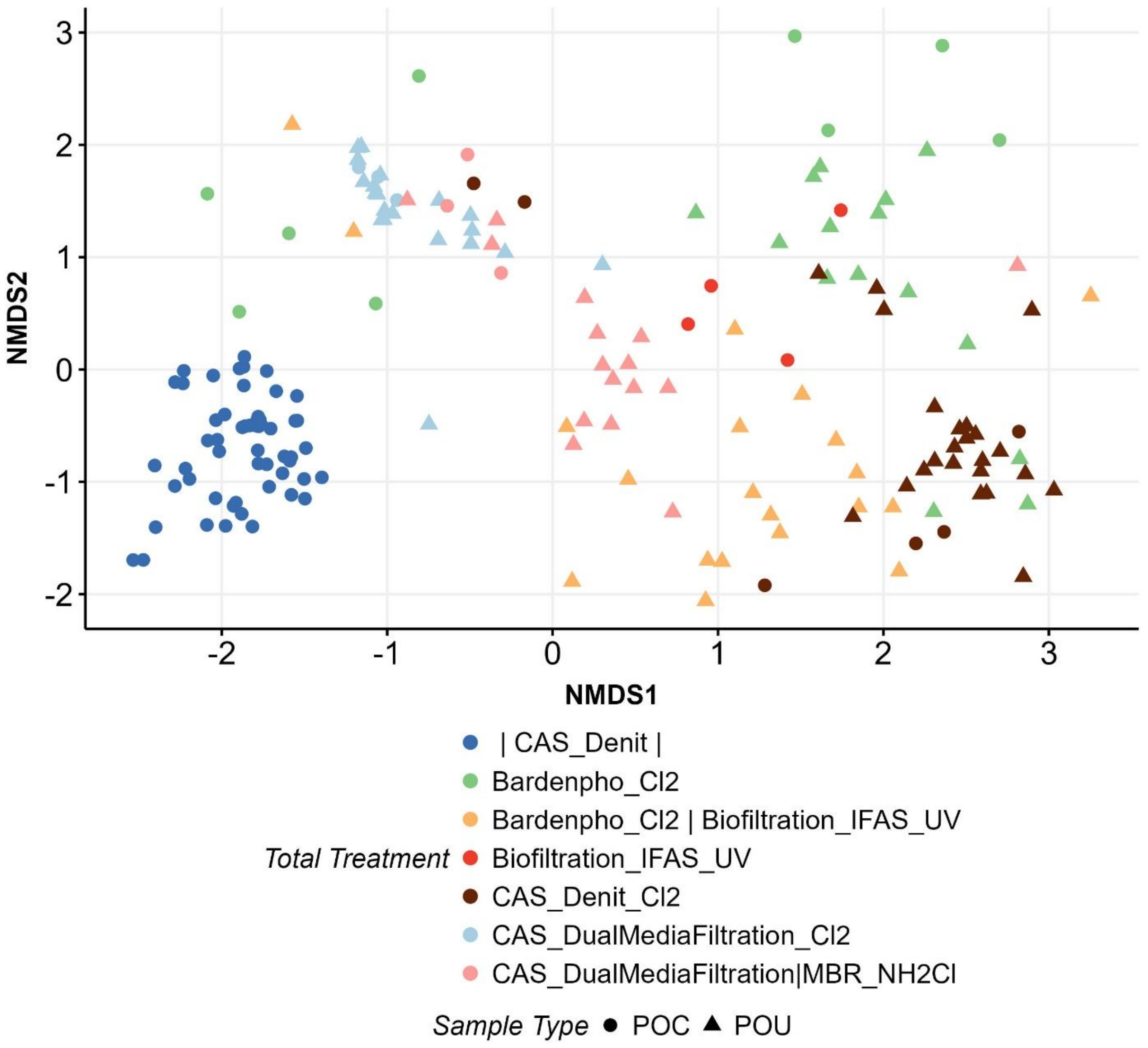

SI Figure 41. Bray-Curtis beta diversity plot for all non-potable reuse bulk water samples, classified by employed treatment processes.

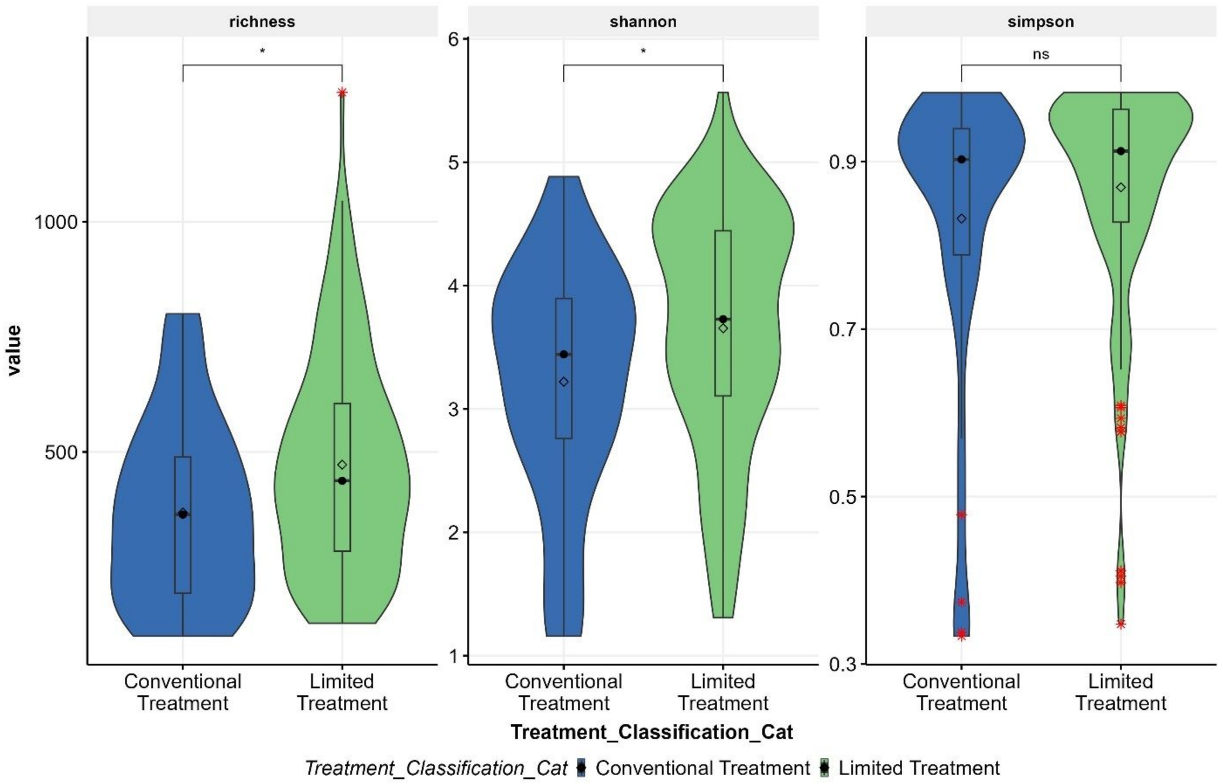

Figure 42. Violin plots of selected alpha diversity metrics for non-potable reuse samples grouped by categorized treatment. Wilcoxon rank sum tests, with p-value adjustment, were used to test significant differences between comparisons.

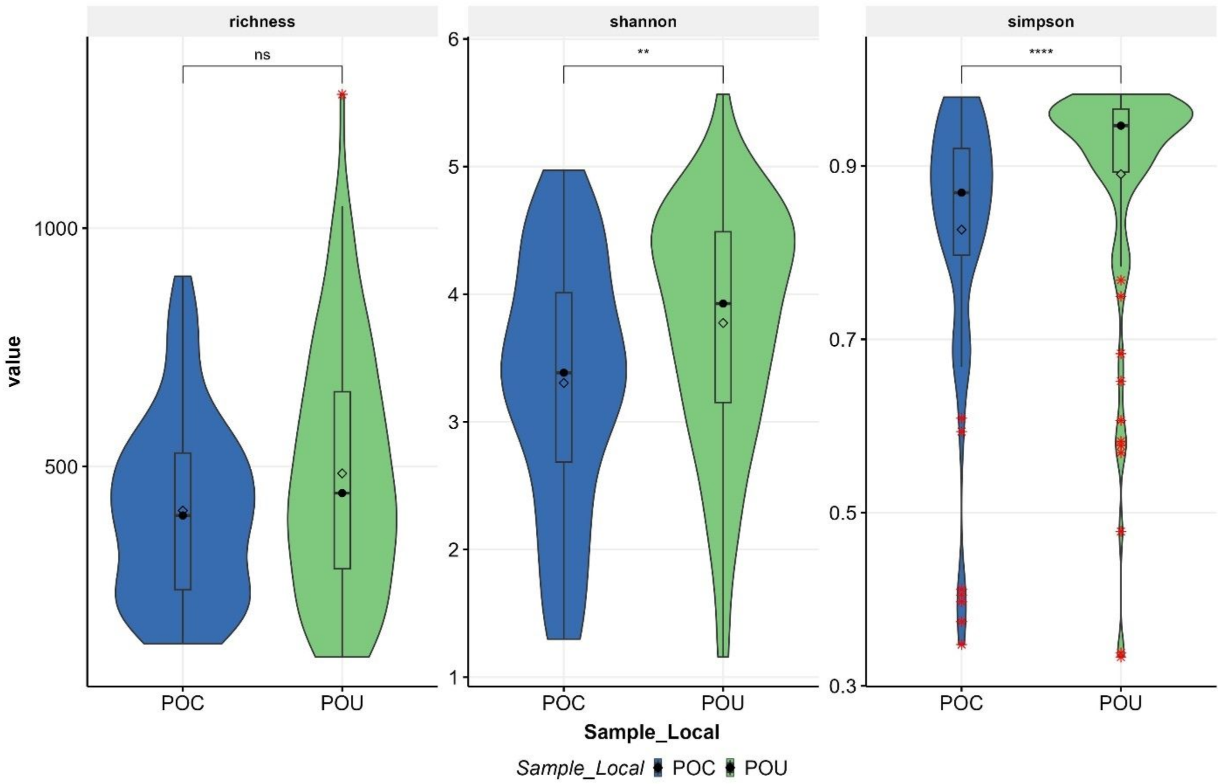

Figure 43. Violin plots of selected alpha diversity metrics for non-potable reuse samples grouped by sample location. Wilcoxon rank sum tests, with p-value adjustment, were used to test significant differences between comparisons.

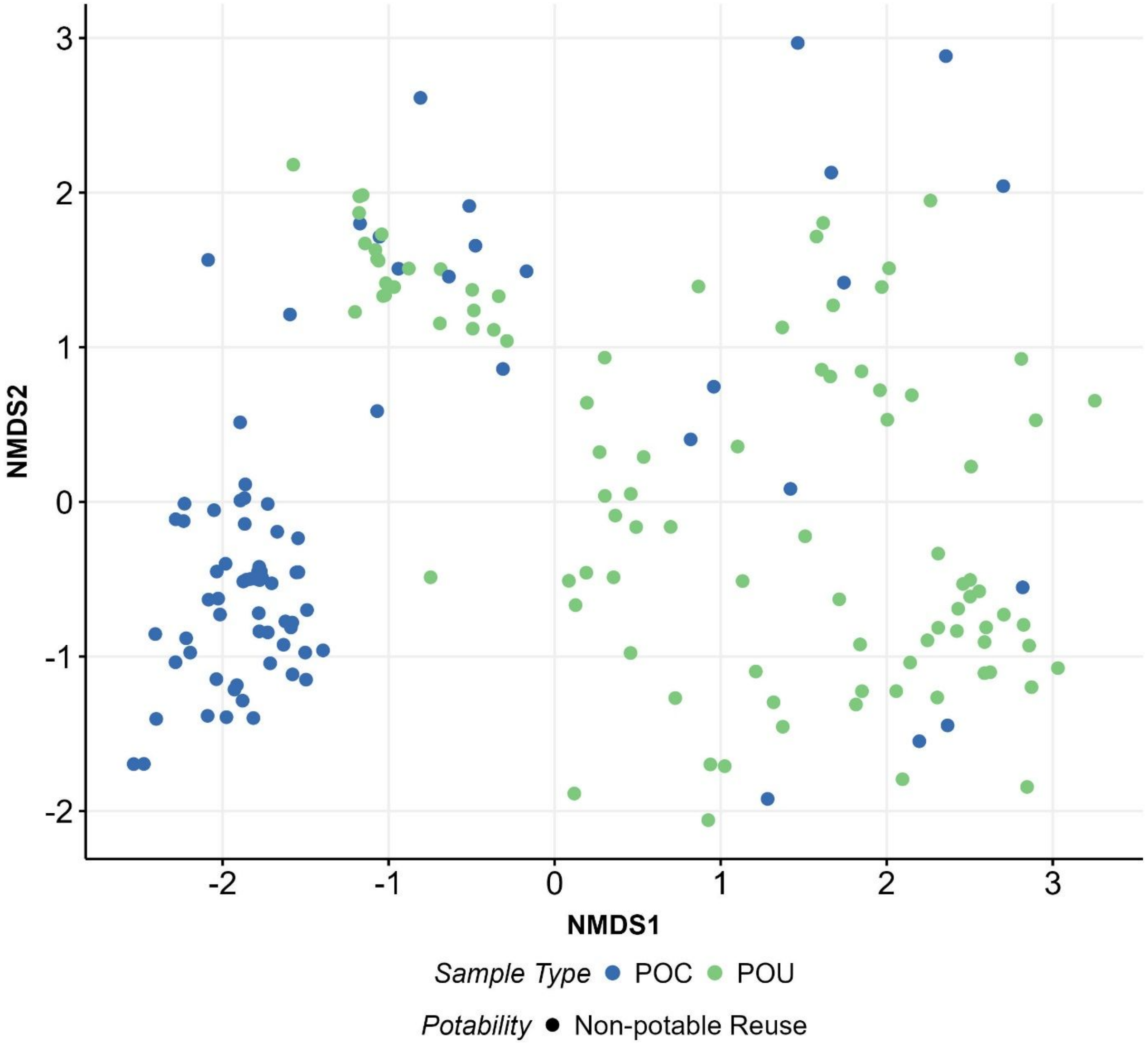

SI Figure 44. Bray-Curtis beta diversity plot for all non-potable reuse bulk water samples, classified by POC or POU.

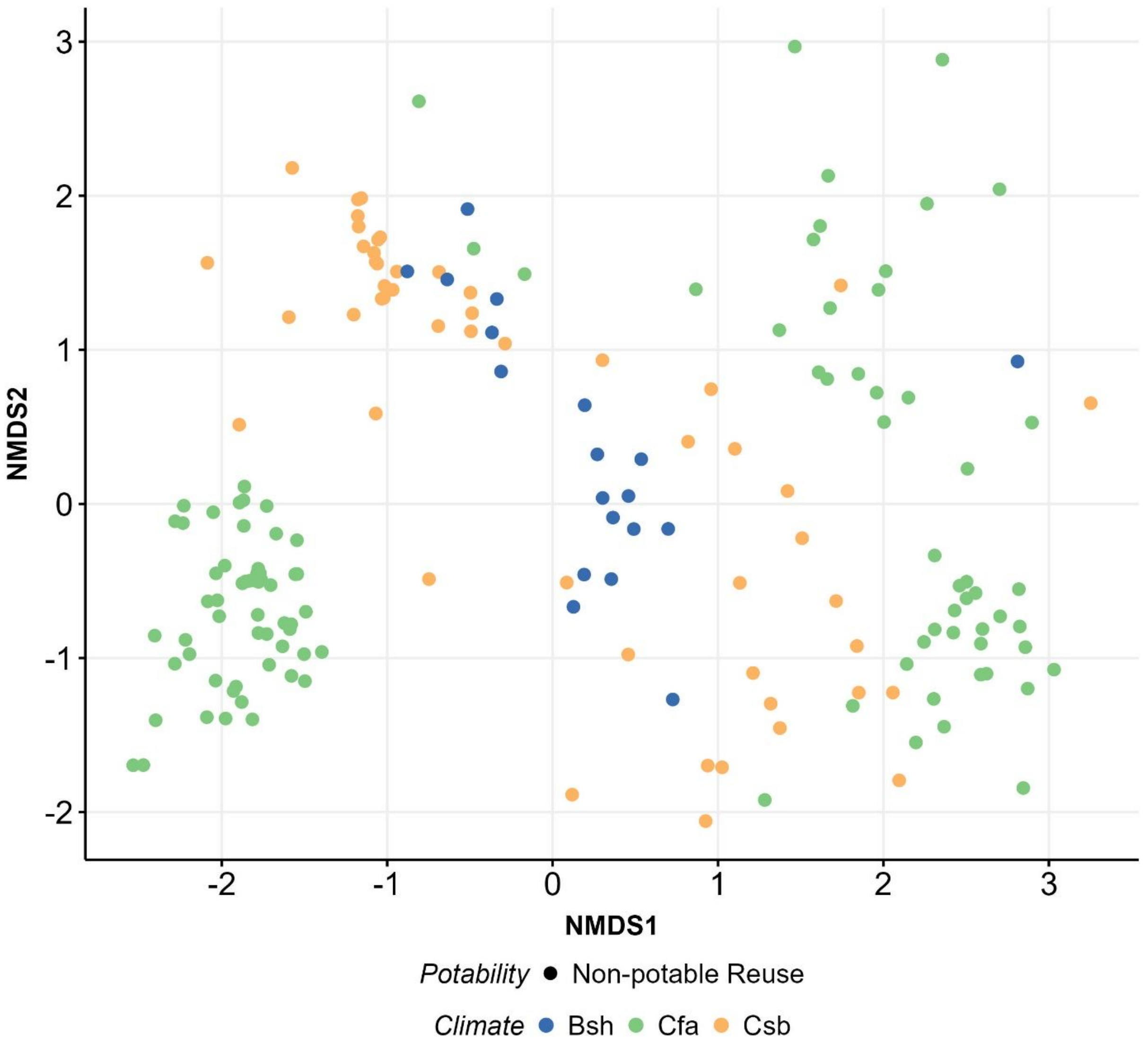

SI Figure 45. Bray-Curtis beta diversity plot for all non-potable reuse bulk water samples, classified by climate designator.

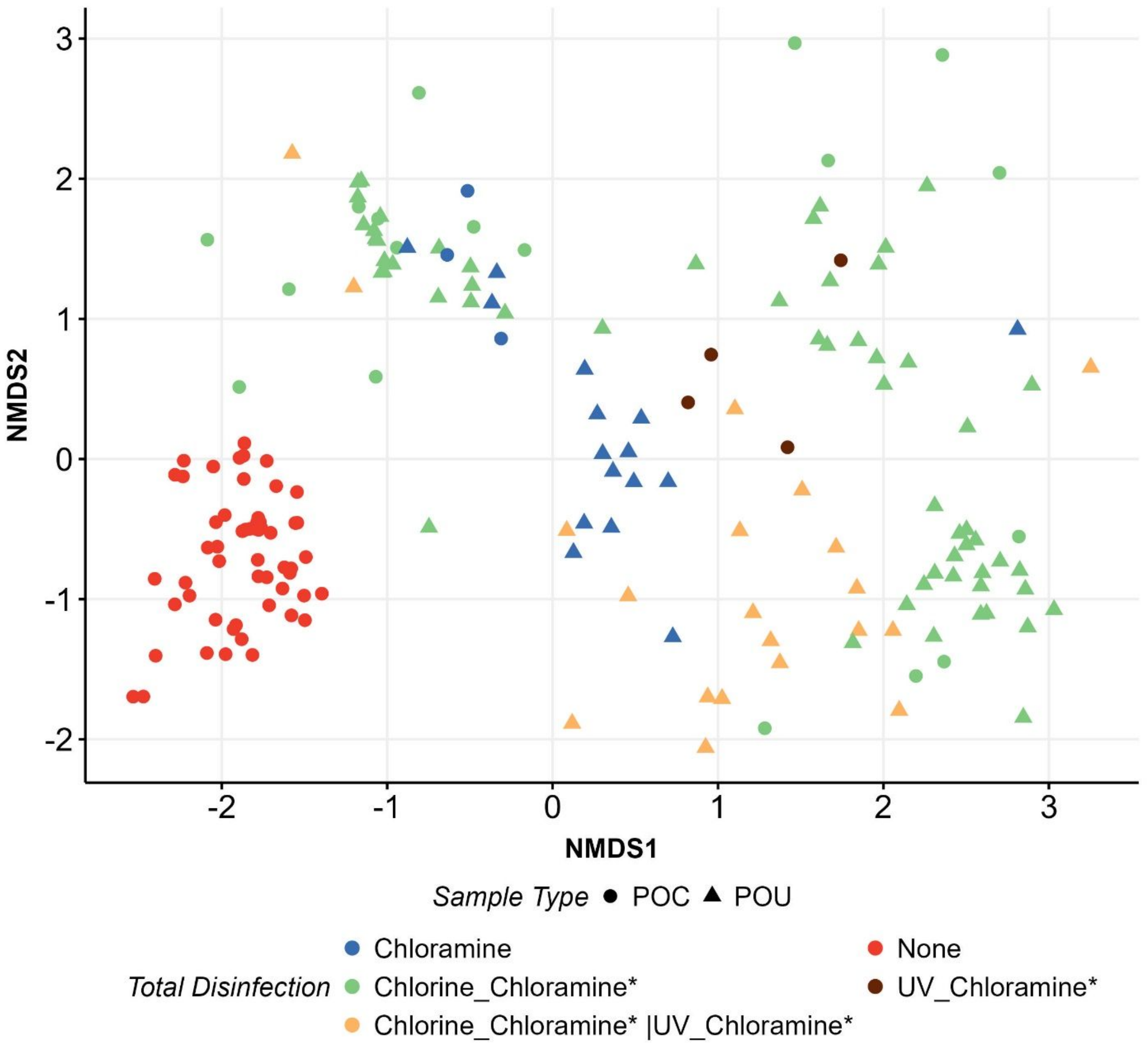

SI Figure 46. Bray-Curtis beta diversity plot for all non-potable reuse bulk water samples, classified by employed disinfectant.
